# Non-antiarrhythmic pharmacotherapy in cardio-renal-metabolic disease and incident atrial fibrillation: a trial meta-analysis

**DOI:** 10.1093/eurheartj/ehag021

**Published:** 2026-01-28

**Authors:** Keerthenan Raveendra, Ramesh Nadarajah, Harriet Larvin, Maryum Farooq, Mohammad Haris, Uther Cutting, Jing Kang, Chris Wilkinson, Mayank Dalakoti, Dipak Kotecha, Gregory Y H Lip, Giuseppe Boriani, Alan John Camm, Isabelle C Van Gelder, Jianhua Wu, Chris P Gale

**Affiliations:** Faculty of Medicine and Health, University of Leeds, Leeds, UK; Leeds Institute for Cardiovascular and Metabolic Medicine, University of Leeds, Leeds, UK; Leeds Institute of Data Analytics, University of Leeds, Leeds, UK; Department of Cardiology, Leeds Teaching Hospitals NHS Trust, Leeds, UK; Wolfson Institute of Population Health, Queen Mary University of London, London, UK; Department of Cardiology, Calderdale and Huddersfield NHS Trust, Huddersfield, UK; Leeds Institute for Cardiovascular and Metabolic Medicine, University of Leeds, Leeds, UK; Leeds Institute of Data Analytics, University of Leeds, Leeds, UK; Faculty of Medicine and Health, University of Leeds, Leeds, UK; Oral Clinical Research Unit, Faculty of Dentistry Oral Craniofacial Sciences, King’s College London, London, UK; Hull York Medical School, University of York, York, UK; Academic Cardiovascular Unit, South Tees NHS Foundation Trust, James Cook University Hospital, Middlesbrough, UK; National University Heart Centre, National University Hospital, Singapore, Singapore; Cardiovascular Metabolic Disease Translational Research Programme, National University of Singapore, Singapore, Singapore; Institute of Cardiovascular Sciences, University of Birmingham, Edgbaston, Birmingham, UK; NIHR Birmingham Biomedical Research Centre, University Hospitals Birmingham NHS Trust, Birmingham, UK; Liverpool Centre for Cardiovascular Science at University of Liverpool, Liverpool John Moores University and Liverpool Heart & Chest Hospital, Liverpool, UK; Cardiovascular and Metabolic Medicine, Institute of Life Course and Medical Sciences, University of Liverpool, Liverpool, UK; Danish Centre for Health Services Research, Department of Clinical Medicine, Aalborg University, Aalborg, Denmark; Cardiology Division, Department of Biomedical, Metabolic and Neural Sciences, University of Modena and Reggio Emilia, Polyclinic of Modena, Modena, Italy; Molecular and Clinical Sciences Research Institute, St George’s University of London, London, UK; Department of Cardiology, University of Groningen, University Medical Center Groningen, Groningen, The Netherlands; Wolfson Institute of Population Health, Queen Mary University of London, London, UK; Faculty of Medicine and Health, University of Leeds, Leeds, UK; Leeds Institute for Cardiovascular and Metabolic Medicine, University of Leeds, Leeds, UK; Leeds Institute of Data Analytics, University of Leeds, Leeds, UK

**Keywords:** Atrial fibrillation, Primary prevention, Cardio-renal-metabolic, Pharmacotherapy, Meta-analysis

## Abstract

**Background and Aims:**

Atrial fibrillation (AF) disease burden is increasing. Pharmacotherapy of cardio-renal-metabolic diseases may prevent incident AF. This meta-analysis estimates the effect of different pharmacotherapies on risk of incident AF across cardio-renal-metabolic diseases.

**Methods:**

The Medline, Embase, and Cochrane Central databases were searched to 7 October 2025 for randomized clinical trials (RCTs) comparing the effect of a non-antiarrhythmic cardio-renal-metabolic medication with control or another agent for incident AF. Random-effects meta-analysis using the Mantel–Haenszel method, with between-study variance estimated using the DerSimonian–Laird method, was performed to synthesize risk ratios (RR) with 95% confidence intervals (CI).

**Results:**

Two hundred and forty-nine RCTs involving 745 041 patients were included, of which 207 identified AF through adverse event reports, 161 were placebo-controlled, and 15 had AF as a pre-specified endpoint. In placebo-controlled trials, significant differences in incident AF were observed with treatment of heart failure with reduced ejection fraction with angiotensin-converting enzyme inhibitors and angiotensin receptor blockers (RR 0.69, 95% CI 0.60–0.80), mineralocorticoid receptor antagonists (RR 0.62, 95% CI 0.43–0.90), and sodium-glucose co-transporter 2 (SGLT2) inhibitors (RR 0.62, 95% CI 0.44–0.87); treatment of chronic kidney disease with SGLT2 inhibitors (RR 0.53, 95% CI 0.33–0.85); and treatment of obesity with glucagon-like peptide-1 receptor agonists (RR 0.79, 95% CI 0.63–0.99). However, the number of AF events per trial was low and none were adequately powered for incident AF.

**Conclusions:**

Prospective RCTs with AF as a pre-specified outcome should be integrated into the design of future trials of cardio-renal-metabolic medications to determine whether they reduce incident AF.

## Structured Graphical Abstract

**Structured Graphical Abstract:**
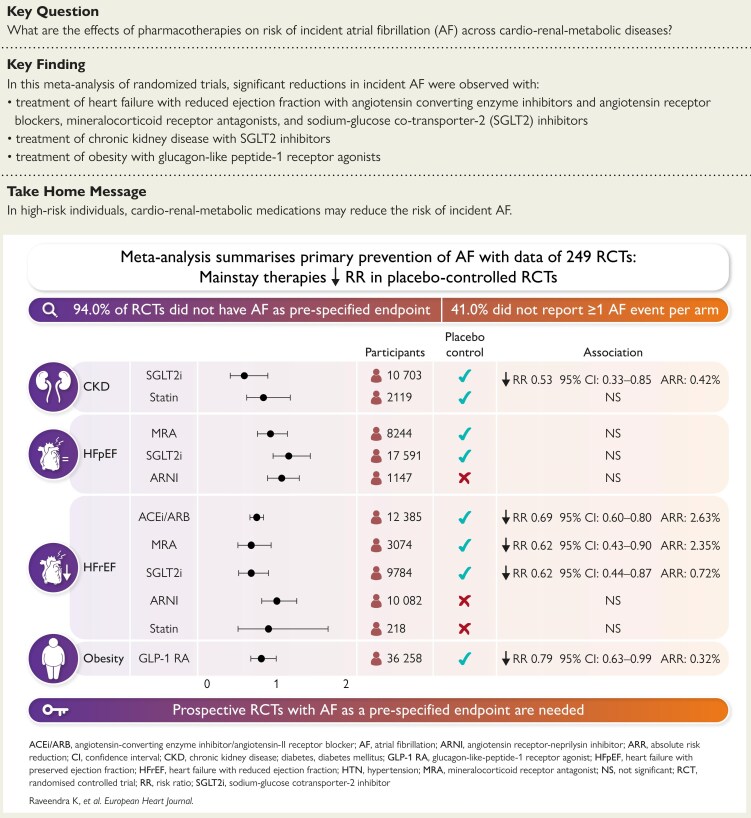

**See the editorial comment for this article ‘Cardio-renal–metabolic management and incident atrial fibrillation: charting the way’, by J.Y. Chyou *et al*., https://doi.org/10.1093/eurheartj/ehag143.**

## Introduction

Atrial fibrillation (AF) confers an increased risk of stroke, heart failure, cognitive decline and death,^1^ and the incidence of AF is rising,^2^ such that by 2060 almost 18 million people may have this condition in Europe.^3^ Preventing the onset of AF before clinical manifestation has clear potential to improve the lives of the general population and reduce the considerable health and social care costs associated with development of AF.^4^

Cardio-renal-metabolic factors and comorbidities—such as hypertension, heart failure, diabetes, chronic kidney disease (CKD), and obesity—substantially increase the risk of developing AF.^5^ Upstream therapy of these cardio-renal-metabolic comorbidities with non-antiarrhythmic medicines may prevent the occurrence of the arrhythmia,^1^ and is recommended in the most recent European Society of Cardiology (ESC) guidelines, albeit with level of evidence B.^4^ Previous systematic reviews and meta-analyses of randomized clinical trials (RCTs) have limited utility to inform patient-centred strategies to prevent AF because they only focus on a pharmacological class rather than the condition being treated,^6–12^ incorporate data for the effect of medicines on recurrence of AF in patients with a known history of AF—rather than first presentation of AF,^6,12^ and include studies in post-operative settings.^8,13^ Furthermore, the most recent guidelines make no mention of the potential for treatment with glucagon-like peptide-1 receptor agonists (GLP-1 RAs) to reduce incident AF in patients with obesity, despite their proven effectiveness for bringing about sustained, clinically relevant reduction in body weight.^14^

To address this knowledge gap, we conducted a systematic review and meta-analysis of the effect of non-antiarrhythmic pharmacotherapies in patients with cardio-renal-metabolic disease on incident AF as reported in published RCTs to inform strategies to reduce the incidence of AF and identify future research priorities.

## Methods

This study was registered on PROSPERO (CRD42024481598) and was reported following the PRISMA statement.^15^

### Data sources and searches

We conducted a systematic search of the Medline, Embase, and Cochrane Central databases from inception to 7 October 2025. A qualified research librarian contributed to the development of the search strategy. We used a combination of keywords and subject headings related to AF and its prevention based on previous literature (see Supplementary data online, *Tables S1*–*S3*).^6,11–13,16–18^ We completed forward and backward citation searching for included studies and previous systematic reviews. Duplicates were removed using Endnote, and then checked manually.

### Study selection criteria

We included RCTs that evaluated the relationship between a pharmacological therapy and the development of incident AF. Included RCTs had to (i) be in adults (aged ≥ 18 years) with a parallel design, (ii) compare a pharmacological agent with a placebo control or another medicine, and (iii) evaluate incident AF as an outcome or adverse event. Studies were excluded if: (i) recurrent AF was included in the outcome definition,^7,18^ (ii) reports included participants who were hospitalized with an acute illness such as acute coronary syndrome or for a procedure (e.g. cardiac surgery) at the point of recruitment given the distinct pathophysiological mechanisms of AF in this acute inflammatory context,^19,20^ or (iii) reports investigated the medicines that are recommended for rate or rhythm control of AF (e.g. amiodarone, beta-blockers, non-dihydropyridine calcium channel blockers).^4^

We focused on the following cardio-renal-metabolic conditions: hypertension, heart failure [both reduced (HFrEF) and preserved ejection fraction (HFpEF)], diabetes mellitus (with and without target organ damage), CKD, and obesity.^5^ Within these conditions, we considered the following non-antiarrhythmic cardio-renal-metabolic medications, which have been referenced in ESC guidelines^1,4^: angiotensin-converting enzyme inhibitor (ACEi), angiotensin receptor blocker (ARB), angiotensin receptor-neprilysin inhibitor (ARNI), GLP-1 RA, mineralocorticoid receptor antagonist (MRA), sodium-glucose co-transporter 2 (SGLT2) inhibitor, dipeptidyl peptidase-4 (DPP-4) inhibitor, statin, and omega-3 fatty acid. Other anti-hypertensives such as dihydropyridine calcium channel blockers and thiazide diuretics have been shown to have no effect on incident AF in previous meta-analyses,^21,22^ and are not referenced in the contemporary ESC guidelines for primary prevention of AF,^4^ and so were not included here.

### Data extraction and quality assessment

Two investigators (K.R., M.F.) independently screened articles for inclusion by title, abstract, full text, and Supplementary data online, materials. Disagreements were resolved by consultation with a third investigator (R.N.). Three investigators (K.R., U.C., and R.N.) independently extracted information from the included studies, including that pertaining to the intervention and outcome, and study and participant characteristics. We extracted the number of cases with AF occurrence in the intervention and control groups, as well as whether AF incidence was a pre-specified efficacy or safety endpoint. Disagreements about data were discussed with two other investigators (H.L. and J.W.). Two investigators (K.R. and M.F.) assessed risk of bias using the Cochrane collaboration’s Risk of Bias 2 (RoB 2) tool.^23^

### Statistical analysis

We calculated unadjusted risk ratios (RR) with 95% confidence intervals (CI) for each study to report effect estimates for AF events. We synthesized the overall RR by indication and class of pharmacotherapies, and by subgroup (indication: diabetes mellitus with and without end-target organ damage, heart failure with reduced or preserved ejection fraction) using a random-effects Mantel–Haenszel model. The between-study variance was estimated using the DerSimonian–Laird method. Statistical heterogeneity was assessed using Cochran’s *Q* and *I*². For meta-analyses with fewer than five studies (*k* < 5), we applied the Hartung–Knapp–Sidik–Jonkman adjustment to derive CI. Trials with zero events in either arm were excluded from the primary RR meta-analysis and do not inform relative effects. We quantified their prevalence and conducted sensitivity analyses including them via Mantel–Haenszel risk difference (finding that the results were directionally consistent with the primary analyses). We present forest plots for the (i) indication for therapy (e.g. HFrEF) separately for placebo and non-placebo controlled trials. Where a class of pharmacotherapy in placebo-controlled trials was found to be associated with a significant difference in relative risk we then present forest plots by individual agents within that class. As individual participant-level data were not available and follow-up durations varied across trials, precise person-time data were lacking. Therefore, we could not derive robust estimates of incidence rates or event rates per year. Instead, we performed a random-effects meta-analysis of absolute risk differences using study-level event counts and sample sizes in treatment and control groups, employing the risk difference measure in the ‘metafor’ package.^24^ We estimated absolute risk reduction (ARR) by pooling the RR across studies and then converting.

Heterogeneity was evaluated using the *I*^2^ statistic and regarded as high if the value was >50%. An *I*^2^ value of 0%–40% would indicate that heterogeneity might not be important, 30%–60% may represent moderate heterogeneity, 50%–90% may represent substantial heterogeneity, and 75%–100% represents considerable heterogeneity. Publication bias was examined by funnel plots and Egger’s test when there were 10 or more trials, with statistical significance set at *P* < .05. We conducted sensitivity analyses to (i) exclude studies at high risk of bias and (ii) exclude studies where AF was not a pre-specified endpoint. Network meta-analyses were not conducted because of the insufficient numbers of events reported in eligible trials. Meta-regression was not conducted as there were too few trials per subgroup for meaningful results. All analyses were conducted using R software (version 3.6.3).^25^

## Results

We reviewed 9371 unique records and included 249 RCTs comprising 745 041 patients (see Supplementary data online, *Figure S1*). Across all trials, the number of patients ranged from 46 to 25 620, the mean age from 41.2 to 76.0 years, the proportion of women from 0.0% to 79.0%.

Of the included RCTs, 15 (6.0%, *n* = 81 338 participants) reported AF as a pre-specified endpoint, whereas 234 (94.0%) reported AF in *post hoc* analysis (*Table 1*). Incident AF was a pre-specified primary endpoint in only one trial.^26^ While AF was a pre-specified endpoint for 5 of 27 RCTs (18.5%) for HFrEF, it was not a pre-specified endpoint for any of the RCTs with obesity indication (*Table 1*). AF ascertainment was by electrocardiogram (ECG) in 5 of 13 (38.4%) included RCTs for vascular disease, 4 of 12 (33.3%) RCTs for hypertension, and 7 of 27 (25.9%) RCTs for HFrEF, but for only 5 of 146 (3.4%) diabetes trials, and 1 of 6 (16.7%) CKD trials, and none of the included obesity trials (*Table 1*). For RCTs utilizing adverse event reports for AF ascertainment, it was not possible to determine if the events were hospitalization for AF or a new AF diagnosis, irrespective of setting (see Supplementary data online, *Tables S5*, *S6*, *S8*, *S10*, *S12*, *S14*, *S16*, *S18*, *S20*, and *S22*).

**Table 1:** Trial characteristics by pharmacological indication

	Indication	CKD	DM	HFpEF	HFrEF	HTN	Obesity	Vasc. disease	Other	Total
RCTs	Total (*n*)	6	146	15	27	12	18	13	12	249
	AF as pre-specified Endpoint	1	1	2	5	1		2	3	15
AF ascertainment	ECG	1	5	2	7	4		5	4	28
	Adverse event	5	140	11	20	6	18	3	4	207
	Medical records							3	1	4
	Not reported		1	2		2		2	3	10
Cohort	Participants (*n*)	13 567	309 054	37 201	39 999	85 687	37 009	90 290	132 030	745 041
	Age (years)	60.65	62.20	70.49	66.05	65.32	54.09	64.90	64.45	63.51
	Male (%)	65.89	60.86	51.54	74.59	55.68	51.23	72.55	62.92	61.91
	HTN (%)	28.04	78.35	96.08	63.24	100.00	63.47	52.38	59.26	72.69
	DM (%)	85.39	100.00	39.47	34.19	24.09	2.63	23.33	25.01	56.78
	HF (%)	12.94	12.41	100.00	100.00	0.21	25.87	3.81	0	18.04

AF, atrial fibrillation; CKD, chronic kidney disease; DM, diabetic mellitus; ECG, electrocardiogram; HFpEF, heart failure with preserved ejection fraction; HFrEF, heart failure with reduced ejection fraction; HTN, hypertension; RCT, randomized clinical trial.

### Hypertension

#### Placebo-controlled RCTs

For patients with hypertension, two placebo-controlled trials evaluated statins including 1887 participants. Treatment with statins (RR 1.01, 95% CI 0.75–1.36, *I*^2^ = 0.0%) (see Supplementary data online, *Figure S2*) was associated with no significant difference with regard to incident AF.

#### Non-placebo-controlled RCTs

For patients with hypertension, three non-placebo-controlled trials evaluated ACEis including 33 800 participants, two evaluated ARBs including 23 329 participants, four evaluated ARNIs including 3641 participants, and one evaluated MRAs including 118 participants. Treatment with ACEis/ARBs was associated with a 11% relative risk reduction (RR 0.89, 95% CI 0.81–0.97, *I*^2^ = 76.0%) (see Supplementary data online, *Figure S3*) and 0.42% ARR (95% CI 0.12%–0.72%). Treatment with ARBs was associated with a 23% relative risk reduction (RR 0.77, 95% CI 0.68–0.87, *I*^2^ = 71.5%) and ARR of 1.08% (95% CI 0.57%–1.59%) (see Supplementary data online, *Figure S3*).

#### Risk of bias, publication bias, and heterogeneity

Risk of bias was moderate (25.0% high, 50.0% moderate, 25.0% low) (see Supplementary data online, *Table S37*). Publication bias was not apparent (see Supplementary data online, *Figure S16*). Heterogeneity was significant for pooled trials of ACEis and ARBs (*I*^2^ = 76.0%), including for trials of ARBs (*I*^2^ = 71.5%).

### Heart failure

#### Heart failure with reduced ejection fraction

##### Placebo-controlled RCTs

**Figure 1:**
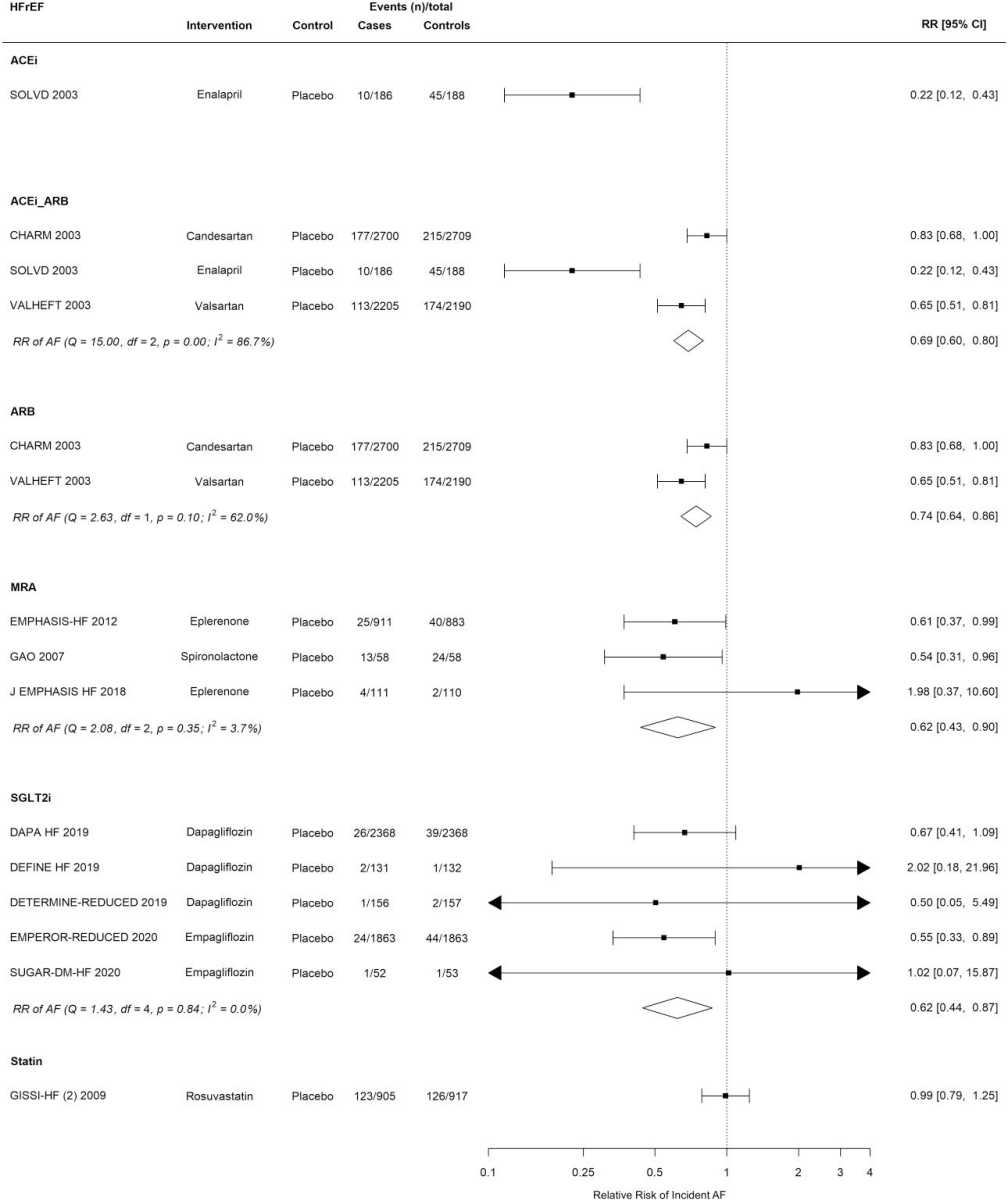
Association between pharmacotherapy for heart failure with reduced ejection fraction indication and incident atrial fibrillation in placebo-controlled trials

For patients with HFrEF, one placebo-controlled trial evaluated ACEi including 391 participants, two evaluated ARBs including 11 994 participants, three evaluated MRAs including 3074 participants, eight evaluated SGLT2 inhibitors including 9784 participants, and one evaluated statins including 1822 participants. Treatment with ACEis/ARBs was associated with a 31% relative risk reduction in incident AF (RR 0.69, 95% CI 0.60–0.80, *I*^2^ = 86.7%; *Figure 1*) and 2.63% ARR (95% CI 1.63%–3.64%). Treatment with MRAs was associated with a 38% relative risk reduction for incident AF (RR 0.62, 95% CI 0.43–0.90, *I*^2^ = 3.7%; *Figure 1*) and 2.35% ARR (95% CI 0.56%–4.14%). Treatment with SGLT2 inhibitors was associated with a 38% relative risk reduction for incident AF (RR 0.62, 95% CI 0.44–0.87, *I*^2^ = 0.0%; *Figure 1*) and 0.72% ARR (95% CI 0.22%–1.23%).

##### Non-placebo-controlled RCTs

For patients with HFrEF, six non-placebo-controlled trials evaluated ARNIs including 10 028 participants, and two evaluated statins including 218 participants. In trials of ARNIs, the control arm was treated with ACEi/ARBs, and no significant difference was observed for AF incidence (RR 0.99, 95% CI 0.77–1.27, *I*^2^ = 0.0%) (see Supplementary data online, *Figure S4*). Trials of statin were controlled with usual care, and showed no significant difference between additional statin intervention and usual care (RR 0.87, 95% CI 0.43–1.73, *I*^2^ = 0.0%) (see Supplementary data online, *Figure S4*).

##### Risk of bias, publication bias, and heterogeneity

Risk of bias was moderate (25.9% high, 37.0% moderate, 37.0% low) (see Supplementary data online, *Table S36*). Publication bias was not apparent (see Supplementary data online, *Figure S15*). There may have been substantial heterogeneity in trials of ACEi/ARB (*I*^2^ = 86.7%), including in trials investigating ARBs (*I*^2^ = 62.0%).

#### Heart failure with preserved ejection fraction

##### Placebo-controlled RCTs

**Figure 2:**
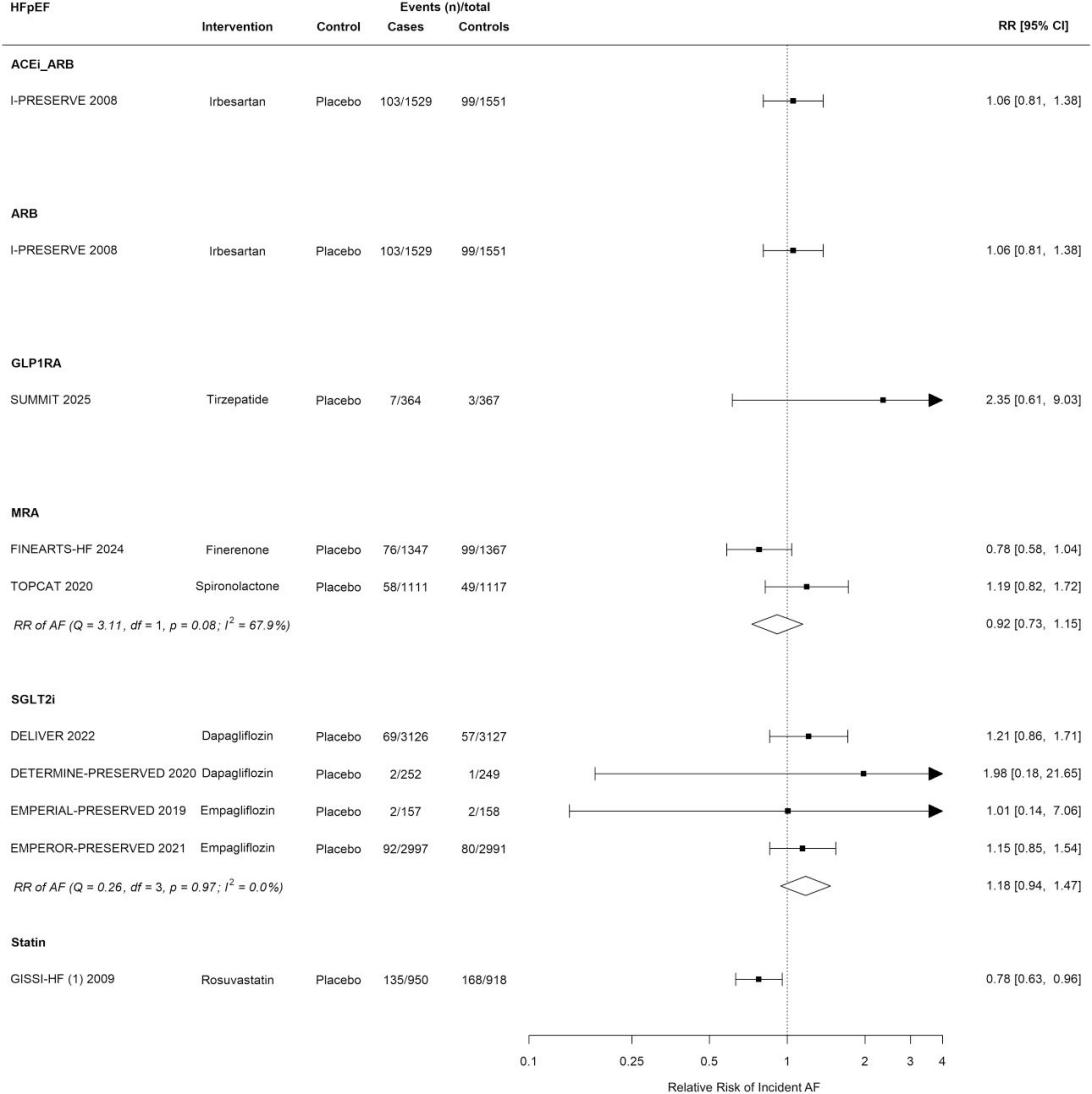
Association between pharmacotherapy for heart failure with preserved ejection fraction indication and incident atrial fibrillation in placebo-controlled trials

For patients with HFpEF, two placebo-controlled trials evaluated MRAs including 8244 participants, six evaluated SGLT2 inhibitors including 17 591 participants, one evaluated ARBs including 3080 participants, one evaluated GLP-1 RAs including 731 participants, and one evaluated statins including 1868 participants. Treatment with neither MRA nor SGLT2 inhibitor was associated with a significant difference in incident AF (MRA: RR 0.92, 95% CI 0.73–1.15, *I*^2^ = 67.9%; SGLT2 inhibitor: RR 1.18, 95% CI 0.94–1.47, *I*^2^ = 0.0%) (*Figure 2*).

##### Non-placebo-controlled RCTs

For patients with HFpEF, three trials evaluated ARNIs compared with ACEi/ARB including 7695 participants, and one evaluated ARBs with 1147 participants. Treatment with ARNIs did not show a significant difference for risk of incident AF (RR 1.07, 95% CI 0.87–1.32, *I*^2^ = 0.0%) (see Supplementary data online, *Figure S5*).

##### Risk of bias, publication bias, and heterogeneity

Risk of bias was moderate (33.3% high, 13.3% moderate, 53.3% low) (see Supplementary data online, *Table S35*). Publication bias was not apparent in pooled heart failure indications (see Supplementary data online, *Figure S15*). There may have been substantial heterogeneity in trials of MRA (*I*^2^ = 67.9%).

### Diabetes mellitus

#### Placebo-controlled RCTs

For patients with diabetes, two placebo-controlled trials evaluated ACEis including 14 247 participants, two trials evaluated ARBs including 15 232 participants, 29 evaluated SGLT2 inhibitors including 71 279 participants, 42 evaluated GLP-1RAs including 88 395 participants, four evaluated MRAs including 16 387 participants, three evaluated DPP-4 inhibitors including 26 074 participants and two evaluated statins including 5248 participants. Treatment with none of ACEis/ARBs (RR 0.98, 95% CI 0.86–1.12, *I*^2^ = 0.0%), MRAs (RR 0.86, 95% CI 0.72–1.03, *I*^2^ = 68.3%), statins (RR 0.83, 95% CI 0.60–1.16, *I*^2^ = 0.0%), SGLT2 inhibitors (RR 0.88, 95% CI 0.76–1.04, *I*^2^ = 0.0%), and GLP-1 RAs (RR 0.96, 95% CI 0.86–1.06, *I*^2^ = 14.4%) showed a significant difference in risk of AF (see Supplementary data online, *Figure S6A*–*C*). This was consistent when subcategorizing trials into those inclusive of patients with and without target organ damage (see Supplementary data online, *Figures S7* and *S8*).

#### Non-placebo-controlled RCTs

For patients with diabetes, 18 non-placebo-controlled RCTs evaluated SGLT2 inhibitors including 16 829 participants, 40 evaluated GLP-1 RA including 32 200 participants, one evaluated MRA including 818 participants, one evaluated DPP-4 inhibitor including 6033 participants, and one evaluated ARB including 16 514 participants. Treatment with neither GLP-1 RAs (RR 1.17, 95% CI 0.60–2.29, *I*^2^ = 0.0%) nor SGLT2 inhibitors (RR 1.01, 95% CI 0.30–3.46, *I*^2^ = 49.5%) showed a significant difference in risk of AF (see Supplementary data online, *Figure S9A*–*C*). This was consistent when subcategorizing trials into those inclusive of patients with and without target organ damage (see Supplementary data online, *Figures S10* and *S11*).

#### Risk of bias, publication bias, and heterogeneity

Risk of bias was moderate (17.8% high, 41.7% moderate, 40.5% low) (see Supplementary data online, *Table S34*). Publication bias was not apparent across all diabetic indications (see Supplementary data online, *Figure S14*), and heterogeneity may not have been important except in ACEi trials (*I*^2^ = 51.9%) and MRA trials (*I*^2^ = 68.3%).

### Chronic kidney disease

#### Placebo-controlled RCTs

**Figure 3:**
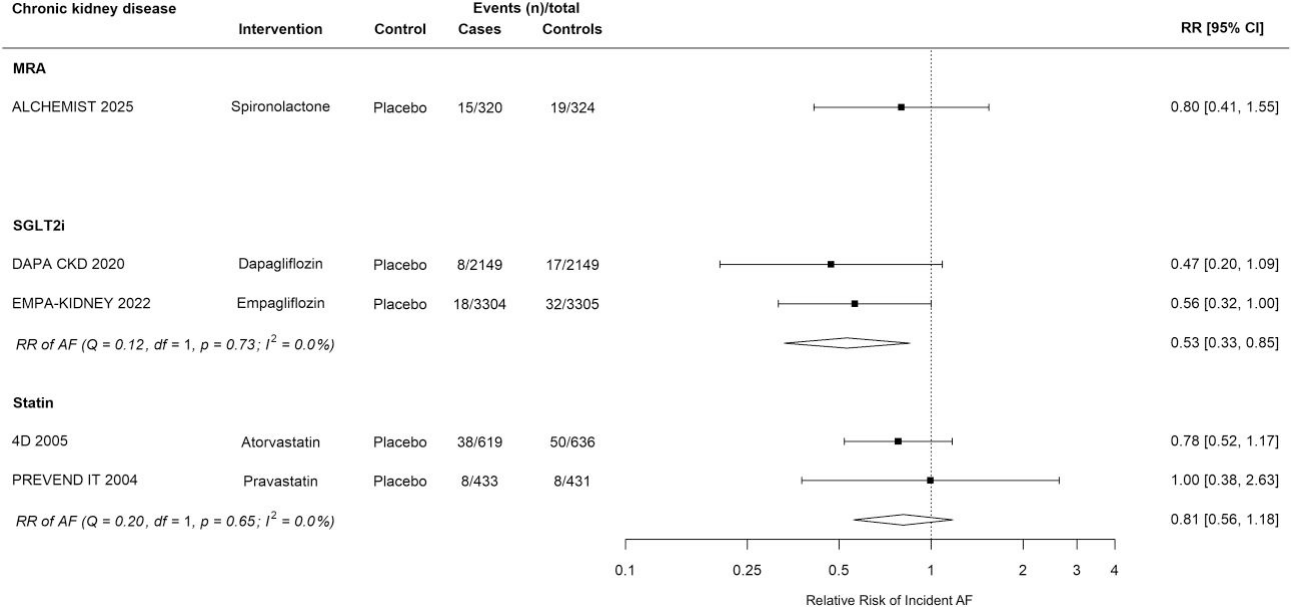
Association between pharmacotherapy for chronic kidney disease indication and incident atrial fibrillation in placebo-controlled trials

For patients with CKD, two placebo-controlled trials evaluated statins including 2119 participants, two evaluated SGLT2 inhibitors including 10 703 participants, one evaluated GLP-1 RA including 101 participants, and one evaluated MRA including 644 participants. Treatment with SGLT2 inhibitors was associated with a 47% relative risk reduction (RR 0.53, 95% CI 0.33–0.85, *I*^2^ = 0.0%) (*Figure 3*) and 0.42% ARR (95% CI 0.11%–0.73%).

#### Risk of bias, publication bias, and heterogeneity

Risk of bias was moderate (16.7% high, 50.0% moderate, 33.3% low) (see Supplementary data online, *Table S33*). Heterogeneity may not have been important here (SGLT2 inhibitor: *I*^2^ = 0.0%; statin: *I*^2^ = 0.0%).

### Obesity

#### Placebo-controlled RCTs

**Figure 4:**
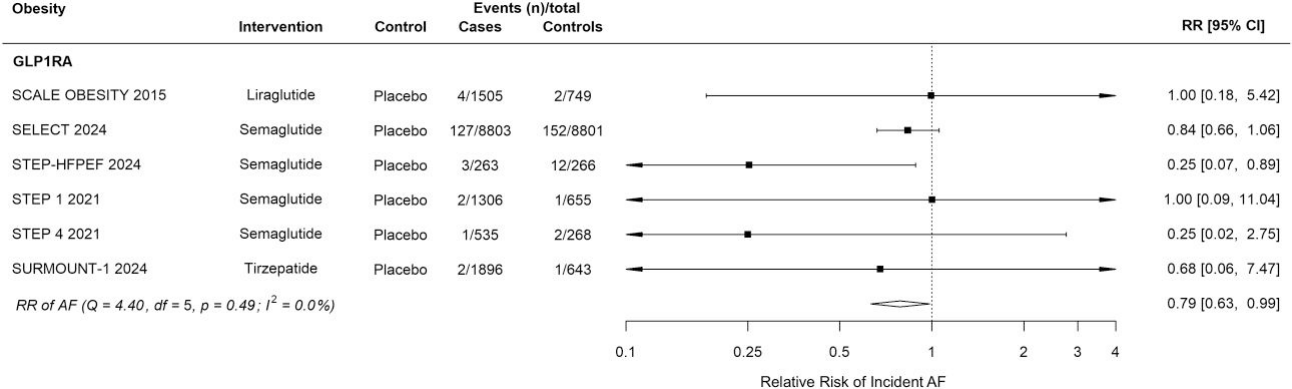
Association between pharmacotherapy for obesity indication and incident atrial fibrillation in placebo-controlled trials

For patients with obesity, 17 placebo-controlled trials evaluated GLP-1 RAs including 36 258 participants. Treatment with GLP-1 RA therapy led to a 21% relative risk reduction of incident AF (RR 0.79, 95% CI 0.63–0.99, *I*^2^ = 0.0%) (*Figure 4*) and 0.32% ARR (95% CI 0.02%–0.62%).

#### Non-placebo-controlled RCTs

For patients with obesity, one non-placebo-controlled trial evaluated GLP-1 RA including 751 participants, and thus meta-analysis was not conducted.

#### Risk of bias, publication bias, and heterogeneity

Risk of bias was low (16.7% moderate and 83.3% low) (see Supplementary data online, *Table S38*). Publication bias was not apparent (see Supplementary data online, *Figure S17*). Heterogeneity may not have been important here (GLP-1 RA: *I*^2^ = 0.0%).

### Vascular disease

#### Placebo-controlled RCTs

For patients with vascular disease (as defined in Supplementary data online, *Table S4*), one placebo-controlled trial evaluated ARB including 20 332 participants, two evaluated omega-3 fatty acid including 13 519 participants, one evaluated SGLT2 inhibitor including 105 participants, and six evaluated statins including 50 961 participants. Treatment with a statin was associated with, on average, a 11% increased risk of AF (RR 1.12, 95% CI 1.01–1.24, *I*^2^ = 0.0%), and treatment with omega-3 fatty acid with a 32% increased risk of AF (RR 1.32, 95% CI 1.03–1.70, *I*^2^ = 33.9%) (see Supplementary data online, *Figure S12*).

#### Non-placebo-controlled RCTs

For patients with vascular disease, one non-placebo-controlled trial evaluated omega-3 fatty acid including 2460 participants, and one trial evaluated statin including 2442 participants. Therefore, meta-analysis was not conducted (see Supplementary data online, *Figure S13*).

#### Risk of bias, publication bias, and heterogeneity

Risk of bias was moderate (23.1% high, 46.1% moderate, 30.8% low) (see Supplementary data online, *Table S39*). Publication bias was not apparent (see Supplementary data online, *Figure S18*). Heterogeneity may not have been important (omega-3 fatty acid: *I*^2^ = 33.9%; statin: *I*^2^ = 0.0%).

### Medicines within pharmacological classes

#### ACEi/ARB for heart failure with reduced ejection fraction

Amongst three placebo-controlled trials of ACEis and ARBs in HFrEF, no single agent was trialled more than once, and therefore meta-analysis of individual agents could not be conducted.

#### MRA for heart failure with reduced ejection fraction

Two of the three placebo-controlled trials of MRAs for HFrEF indication trialled eplerenone and included 2015 participants. Meta-analysis did not find a significant relative risk difference (RR 0.67, 95% CI 0.42–1.07, *I*^2^ = 43.5%) (see Supplementary data online, *Figure S19*). Risk of bias was high in both trials (see Supplementary data online, *Table S29*).

#### SGLT2 inhibitors for heart failure with reduced ejection fraction

Amongst five placebo-controlled trials of SGLT2 inhibitors in HFrEF indication, three trialled dapagliflozin and included 5312 participants. Meta-analysis did not find a significant relative risk difference (RR 0.69, 95% CI 0.43–1.11, *I*^2^ = 0.0%) (see Supplementary data online, *Figure S20*). Two of the five placebo-controlled trials trialled empagliflozin and included 3831 participants. Meta-analysis found a 44% decreased relative risk (RR 0.56, 95% CI 0.34–0.90, *I*^2^ = 0.0%) (see Supplementary data online, *Figure S20*).

Heterogeneity may not have been important in the empagliflozin finding (*I*^2^ = 0.0%). Risk of bias was high in trials of dapagliflozin (66.7% high, 33.3% moderate) (see Supplementary data online, *Table S31*), and moderate in those of empagliflozin (50% moderate, 50% low) (see Supplementary data online, *Table S31*).

#### GLP-1 RA for obesity

Amongst four placebo-controlled trials of GLP-1 RAs in obesity indication including 20 897 participants, treatment with semaglutide was associated with decreased risk of AF (RR 0.79, 95% CI 0.63–0.99) (see Supplementary data online, *Figure S21*). Heterogeneity may not have been important here (*I*^2^ = 30.5%). Risk of bias in these trials was low (75% low, 25% moderate) (see Supplementary data online, *Table S28*).

### Sensitivity analysis

#### Risk of bias

When RCTs at high risk of bias were excluded from meta-analysis, the relative risk reduction for incident AF in placebo-controlled trials of treatment with SGLT2 inhibitor in patients with CKD, treatment with SGLT2 inhibitor in HFrEF, and treatment with GLP-1 RA in obesity was preserved. Four of eight trials testing ACEi/ARB in hypertension and HFrEF were at high risk of bias, as were two of three trials testing MRA in HFrEF, and thus the reduced incidence of AF with these treatments could not be confirmed on this sensitivity analysis.

#### AF as a pre-specified endpoint

AF was a pre-specified endpoint in 15 trials, with one evaluating ACEi, three evaluating ARBs, one evaluating ARNI, six evaluating MRAs, three evaluating omega-3 fatty acid, and one evaluating statin. Omega-3 fatty acid was associated with an increase in the relative risk of AF (RR 1.26, 95% CI 1.11–1.42). No significant difference was seen for AF incidence with the other trialled pharmacological classes (see Supplementary data online, *Figure S22*).

## Discussion

This contemporary systematic review and meta-analysis of 249 RCTs provides hypothesis-generating data for the association of commonly used pharmacotherapies in patients with cardio-renal-metabolic diseases and incident AF. AF was a pre-specified endpoint in only 6% of trials and ascertained by adverse event reports in 83% of trials. Thus, the number of AF events per trial was low, and none of the trials were adequately powered to inform whether these pharmacological classes are protective of AF. With the available data from placebo-controlled trials a reduction in incident AF was observed for treatment of HFrEF with ACEi/ARB, MRA, and SGLT2 inhibitor, for treatment of CKD with SGLT2 inhibitors, and the treatment of obesity with GLP-1 RAs, including semaglutide specifically (*Structured Graphical Abstract*). However, prospective RCTs with AF as a primary outcome and with systematic AF detection are required to determine if cardio-renal-metabolic medicines are protective for incident AF.

### Comparison with previous individual studies

The key strength of our study is its sample size and breadth and relevance to clinical practice. Previous studies have focussed on the effect of a single pharmacological class,^6,8,11–13,16,17,27^ but the effect of a cardio-renal-metabolic pharmacotherapy on AF will depend on the underlying substrate, and the relationship of that disease to AF. By contrast, by considering the effect of agents in a specific cardio-renal-metabolic disease, we provide an analysis that is more relevant to patient care.

Our results concord with treatment recommended in current ESC guidelines for prevention of AF. ACEis and ARBs are recommended first line for the management of hypertension to reduce the risk of AF.^4^ Furthermore, we add to data from existing meta-analyses that guideline-directed treatment for HFrEF with ACEis/ARBs, MRAs, and SGLT2 inhibitors may be associated with reduction in incident AF.^6^ We extend beyond previous analyses by investigating trials for patients with HFpEF, though we did not observe a significant difference in incident AF from any medicine.^18^

In contrast to previous studies, we did not find that SGLT2 inhibitor treatment was associated with a reduction in incidence of AF in patients with diabetes.^11,27^ These differences may reflect the choice of inclusion criteria for RCTs. Our study sought to specifically assess incident, rather than recurrent AF, and for the results to be generalizable to the general population, rather than a specific hospitalized sub-cohort.^28,29^ However, low event rates, as AF was ascertained by adverse event reporting, in trials of SGLT2 inhibitors across diabetes and CKD make it challenging to determine the true nature of the association between SGLT2 inhibitors and incident AF in diabetes and CKD.

### Implications for clinicians, policy makers and future research

First, importantly, we did not find evidence of excess risk of AF with currently recommended guideline-directed treatment of cardio-renal-metabolic conditions, including hypertension, HFrEF, diabetes, and CKD.

Second, while the most recent ESC guidelines emphasized the importance of weight management for prevention of AF, they did not specifically mention GLP-1 RAs. Our results provide hypothesis-generating evidence that GLP-1 RA treatment in obesity may be associated with a reduction in incident AF, but trials included a low number of AF events, only detected as adverse event reports (*Table 1*). In patients with obesity, it is logical that the weight reduction benefit of GLP-1 RA therapy may confer a reduction in incident AF given the wealth of observational evidence that body weight is associated with AF genesis.^30,31^ It is possible that there are direct effects, as the GLP-1 receptor is highly expressed in the atria of the heart,^32^ and in type 2 diabetic murine models, chronic treatment with GLP-1 RA reduced the susceptibility to AF in association with lower levels of atrial fibrosis and improvements in atrial conduction.^33^ However, given the limitations of the available data, prospective randomized evidence is required to determine whether GLP-1 RA therapy has an effect on incident AF in patients with obesity.

Third, the main finding from this study is that RCTs across hypertension, diabetes, heart failure, obesity, and CKD—all high risk disease states for AF—have frequently failed to systematically collect standardized AF information, thereby failing to provide data for the effect of medicines on incident AF. The low event rates attest to likely under-detection of AF in these trials. Novel RCT evidence for these pharmacological classes with the primary outcome of incident AF are required to truly determine if these medicines have an effect on incident AF. Conduct of primary prevention trials for AF has historically been limited by difficulties in identifying groups at sufficiently high risk, the prolonged timescale necessary to achieve an adequate number of relevant endpoints,^34^ and the inability to efficiently and inexpensively diagnose AF as a study end point. These barriers need no longer limit research into primary prevention of AF. There are now unparalleled opportunities to comprehensively estimate AF risk by considering multiple risk factors,^35,36^ and there is now a plethora of inexpensive and non-invasive methods for identifying and characterizing incident AF and AF burden to make robust ascertainment of AF in primary prevention trials more feasible.^37^

### Limitations

Despite this being a meta-analysis of RCTs, the quality of evidence overall must be considered carefully when interpreting these findings. First, only one RCT had incident AF as a primary endpoint and therefore these data may be prone to multiple-testing error and data-derived emphasis biases. Second, there was risk of bias in outcome measurement. That is, the detection of AF is largely dependent on the methods applied, with the frequency of ECG checks being a key determinant for AF detection when symptoms are absent or not specific.^37^ Accordingly, as the included studies were not primarily targeted to enhance AF detection and assess AF burden, it is inevitable that asymptomatic AF may be under-detected,^38^ and the relatively low rate of AF events compared to population incidence estimates furthermore suggests that AF event rates were underestimated.^2^ This may lead to an overestimation of the alterations to relative risk of AF that we observed with some pharmacological classes and a false assessment that there is an effect on AF as a type I error. Third, we used aggregated study-level data rather than individual participant data, which precluded estimates of event rates over time and means that different follow-up periods are included in meta-analysis estimates. Fourth, the exact inclusion criteria and definitions and ascertainment methods for endpoints varied among the included RCTs. Fifth, we were unable to conduct a network meta-analysis between pharmacological classes due to the low numbers of events in eligible RCTs. Sixth, included RCTs did not provide details for the burden and symptoms of AF. Seventh, while we subdivided RCTs by predominant indication for the trial, participants did not have that disease alone and commonly had multiple cardio-renal-metabolic diseases (e.g. diabetes and CKD), which we could not overcome in the absence of individual-patient level data.

## Conclusion

This meta-analysis of data from 249 RCTs is limited by the fact that data are predominantly *post hoc*, often from adverse events reports, and at risk of bias, overestimation, and underestimation with regard to treatment effects. Available data provide hypothesis-generating estimates that treatment of HFrEF with ACEi/ARB, MRA, and SGLT2 inhibitors, treatment of CKD with SGLT2 inhibitors, and treatment of obesity with GLP-1 RAs, may be associated with a relative risk reduction in incident AF. Prospective RCTs with AF as a pre-specified outcome should be integrated into the design of future trials of cardio-renal-metabolic medications to determine whether they reduce incident AF.

## Supplementary Material

ehag021_Supplementary_Data

## Declarations

### Data Availability

Data are available on reasonable request. Technical appendix, statistical code, and dataset are available from the corresponding author at keerthraveendra@gmail.com.

### Pre-registered Clinical Trial Number

CRD42024481598.
