# Supplementary material for: Non-antiarrhythmic pharmacotherapy in cardio-renal-metabolic disease and incident atrial fibrillation: a trial meta-analysis
Source: Eur Heart J. 2026 Jan 28;47(23):2922–33. doi: 10.1093/eurheartj/ehag021 (PMC13268693; doi:10.1093/eurheartj/ehag021)

**Supplementary Material: Forest Plots with Subgroup and Sensitivity Analyses and Funnel Plots**

Non-antiarrhythmic pharmacotherapy in cardio-renal-metabolic disease and incident atrial fibrillation: a trial meta-analysis

Table of Contents

[Summary of literature search 3](#_Toc211776900)

[Figure S1: PRISMA flowchart. 3](#_Toc211776901)

[Association between indication of pharmacotherapy and incident atrial fibrillation by class of pharmacotherapy. 4](#_Toc211776902)

[Figure S2: Association between pharmacotherapy and incident atrial fibrillation for hypertension indication in placebo-controlled trials only. 4](#_Toc211776903)

[Figure S3: Association between pharmacotherapy and incident atrial fibrillation for hypertension indication in non-placebo-controlled trials only. 5](#_Toc211776904)

[Figure S4: Association between pharmacotherapy and incident atrial fibrillation for heart failure with reduced ejection fraction indication in non-placebo-controlled trials only. 6](#_Toc211776905)

[Figure S5: Association between pharmacotherapy and incident atrial fibrillation for heart failure with preserved ejection fraction indication in non-placebo-controlled trials only 7](#_Toc211776906)

[Figure S6a: Association between pharmacotherapy and incident atrial fibrillation for any diabetes indication (ACEi, ARB, DPP4I), in placebo-controlled trials only. 8](#_Toc211776907)

[Figure S6b: Association between pharmacotherapy and incident atrial fibrillation for any diabetes indication (GLP-1 RA), in placebo-controlled trials only. 9](#_Toc211776908)

[Figure S6c: Association between pharmacotherapy and incident atrial fibrillation for any diabetes indication (MRA, SGLT2i, statin), in placebo-controlled trials only. 10](#_Toc211776909)

[Figure S7: Association between pharmacotherapy and incident atrial fibrillation for diabetes mellitus indication, in placebo-controlled trials only. 11](#_Toc211776910)

[Figure S8: Association between pharmacotherapy and incident atrial fibrillation for diabetes with end target organ damage indication, in placebo-controlled trials only. 12](#_Toc211776911)

[Figure S9a: Association between pharmacotherapy and incident atrial fibrillation for any diabetes indication (ARB, DPP4I), in non-placebo-controlled trials only. 13](#_Toc211776912)

[Figure S9b: Association between pharmacotherapy and incident atrial fibrillation for any diabetes indication (GLP-1 RA), in non-placebo-controlled trials only. 14](#_Toc211776913)

[Figure S9c: Association between pharmacotherapy and incident atrial fibrillation any diabetes indication (SGLT2i), in non-placebo-controlled trials only. 15](#_Toc211776914)

[Figure S10: Association between pharmacotherapy and incident atrial fibrillation for diabetes mellitus indication, in non-placebo-controlled trials only. 16](#_Toc211776915)

[Figure S11: Association between pharmacotherapy and incident atrial fibrillation for diabetes with end target organ damage indication, in non-placebo-controlled trials only. 17](#_Toc211776916)

[Figure S12: Association between pharmacotherapy and incident atrial fibrillation for vascular disease indication, in placebo-controlled trials only. 18](#_Toc211776917)

[Figure S13: Association between pharmacotherapy and incident atrial fibrillation for vascular disease indication, in non-placebo-controlled trials only. 19](#_Toc211776918)

[Funnel plots of trials by relevant cardio-renal-metabolic indication. 20](#_Toc211776919)

[Figure S14: Funnel plot of diabetes trials. 20](#_Toc211776920)

[Figure S15: Funnel plot of heart failure trials. 21](#_Toc211776921)

[Figure S16: Funnel plot of hypertension trials. 22](#_Toc211776922)

[Figure S17: Funnel plot of obesity trials. 23](#_Toc211776923)

[Figure S18: Funnel plot of vascular disease trials. 24](#_Toc211776924)

[Association between class of pharmacotherapy and incident atrial fibrillation in placebo-controlled trials of a single indication by individual agent. 25](#_Toc211776925)

[Figure S19: Association between MRAs and incident atrial fibrillation in placebo-controlled heart failure with reduced ejection fraction trials by agent. 25](#_Toc211776926)

[Figure S20: Association between SGLT2is and incident atrial fibrillation in placebo-controlled heart failure with reduced ejection fraction trials by agent. 26](#_Toc211776927)

[Figure S21: Association between GLP-1 RA and incident atrial fibrillation in placebo-controlled obesity trials by agent. 27](#_Toc211776928)

[Association between pharmacotherapy and incident atrial fibrillation in trials with prespecified endpoints. 28](#_Toc211776929)

[Figure S22: Association between pharmacotherapy in incident atrial fibrillation in trials with prespecified endpoints. 28](#_Toc211776930)

[Pharmacotherapies across cardio-renal-metabolic conditions 29](#_Toc211776931)

[Figure S23: Summary plot of overall risk of bias across all 249 trials. 29](#_Toc211776932)

# Summary of literature search

## Figure S1: PRISMA flowchart.

**Identification of studies via databases and registers**

Records identified from*:

Databases:

Medline (n=2412)

Embase (n=7052)

Cochrane (n=1179)

Total (n=10643)

Records removed *before screening*:

Duplicate records removed (n = 1272)

**Identification**

Records excluded

(n = 8922)

Records screened

(n = 9371)

Reports not retrieved

(n = 7)

Reports sought for retrieval

(n = 449)

Reports excluded:

Systematic Review/Review

(n = 98)

Secondary Prevention (n = 47)

Incorrect Setting (n = 38)

Guidelines/Recommendations (n = 14)

Comment/Editorial (n = 12)

Duplicates (n = 6)

Case Report/Case Series

(n = 5)

Non-English study (n = 2)

Reports assessed for eligibility

(n = 442)

**Screening**

Additional records identified through backward/forward citation and expert consultation

(n = 29)

Studies included in review

(n = 249)

Studies included in quantitative synthesis

(n = 147)

**Included**

# Association between indication of pharmacotherapy and incident atrial fibrillation by class of pharmacotherapy.

## Figure S2: Association between pharmacotherapy and incident atrial fibrillation for hypertension indication in placebo-controlled trials only.


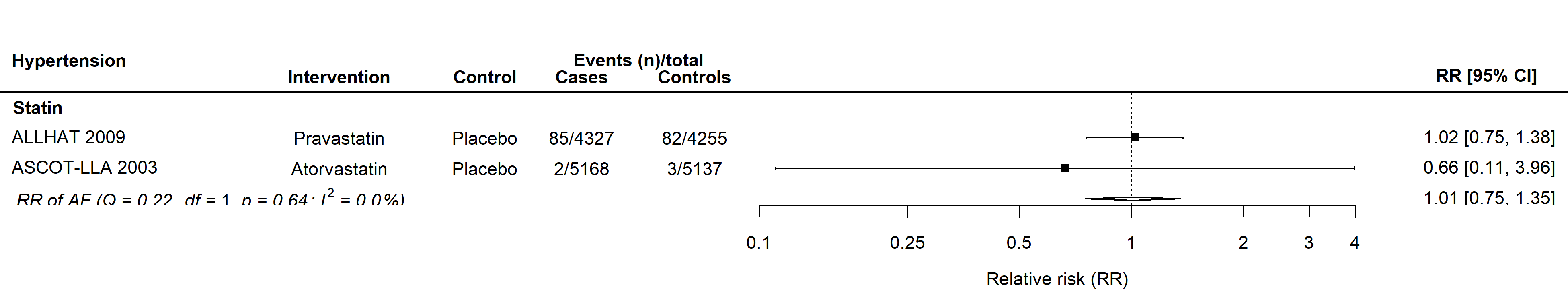


## Figure S3: Association between pharmacotherapy and incident atrial fibrillation for hypertension indication in non-placebo-controlled trials only.


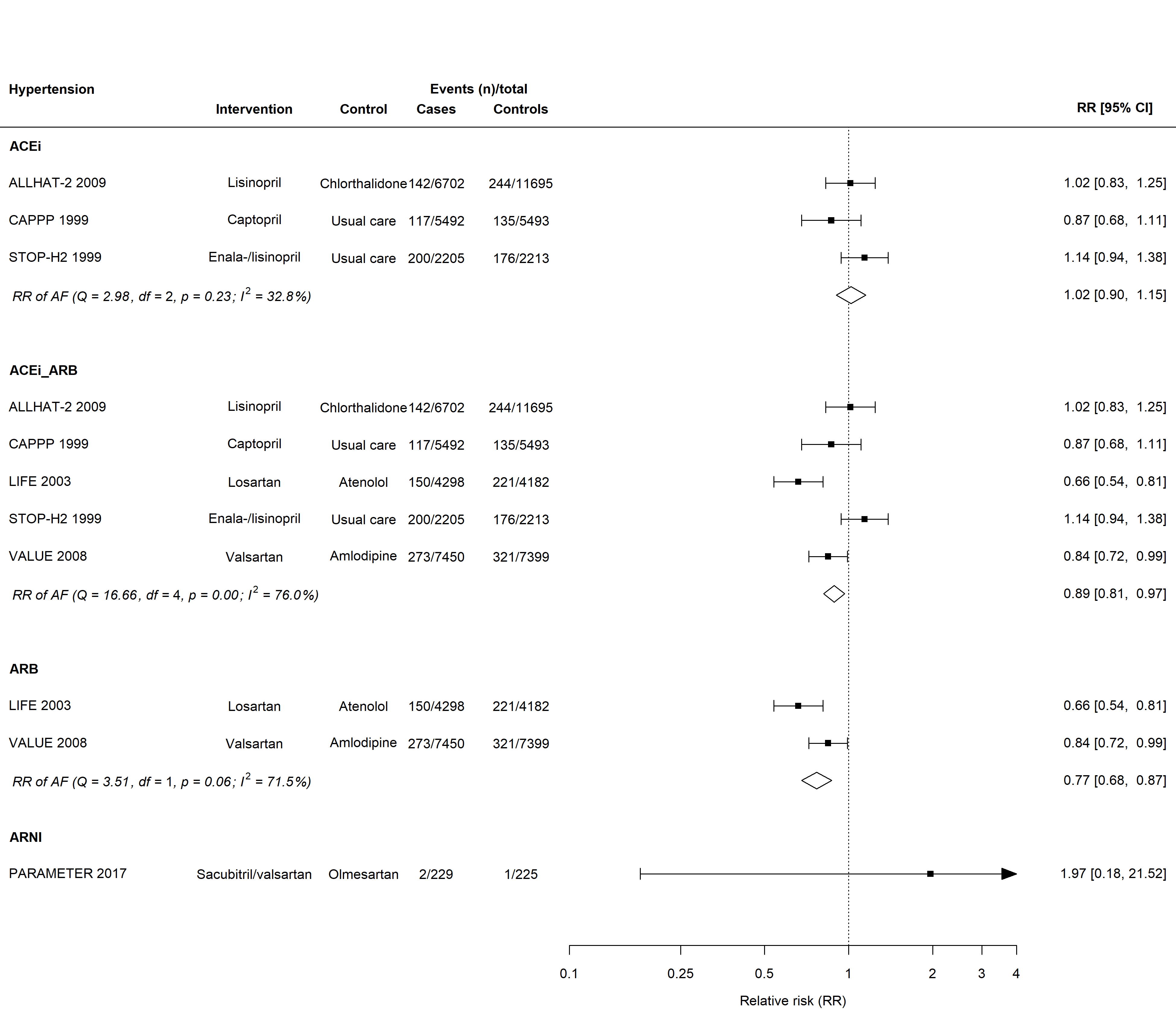


## Figure S4: Association between pharmacotherapy and incident atrial fibrillation for heart failure with reduced ejection fraction indication in non-placebo-controlled trials only.


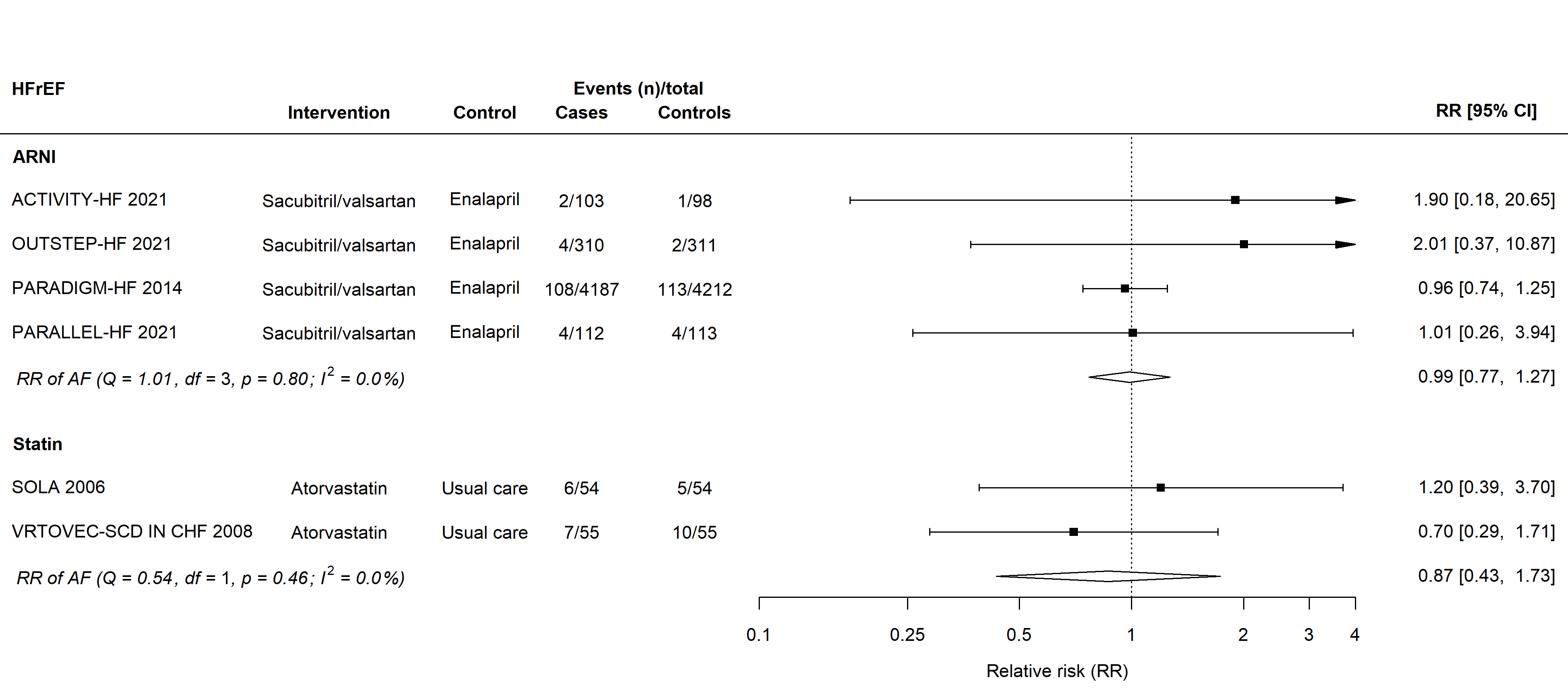


Figure S5: Association between pharmacotherapy and incident atrial fibrillation for heart failure with preserved ejection fraction indication in non-placebo-controlled trials only.
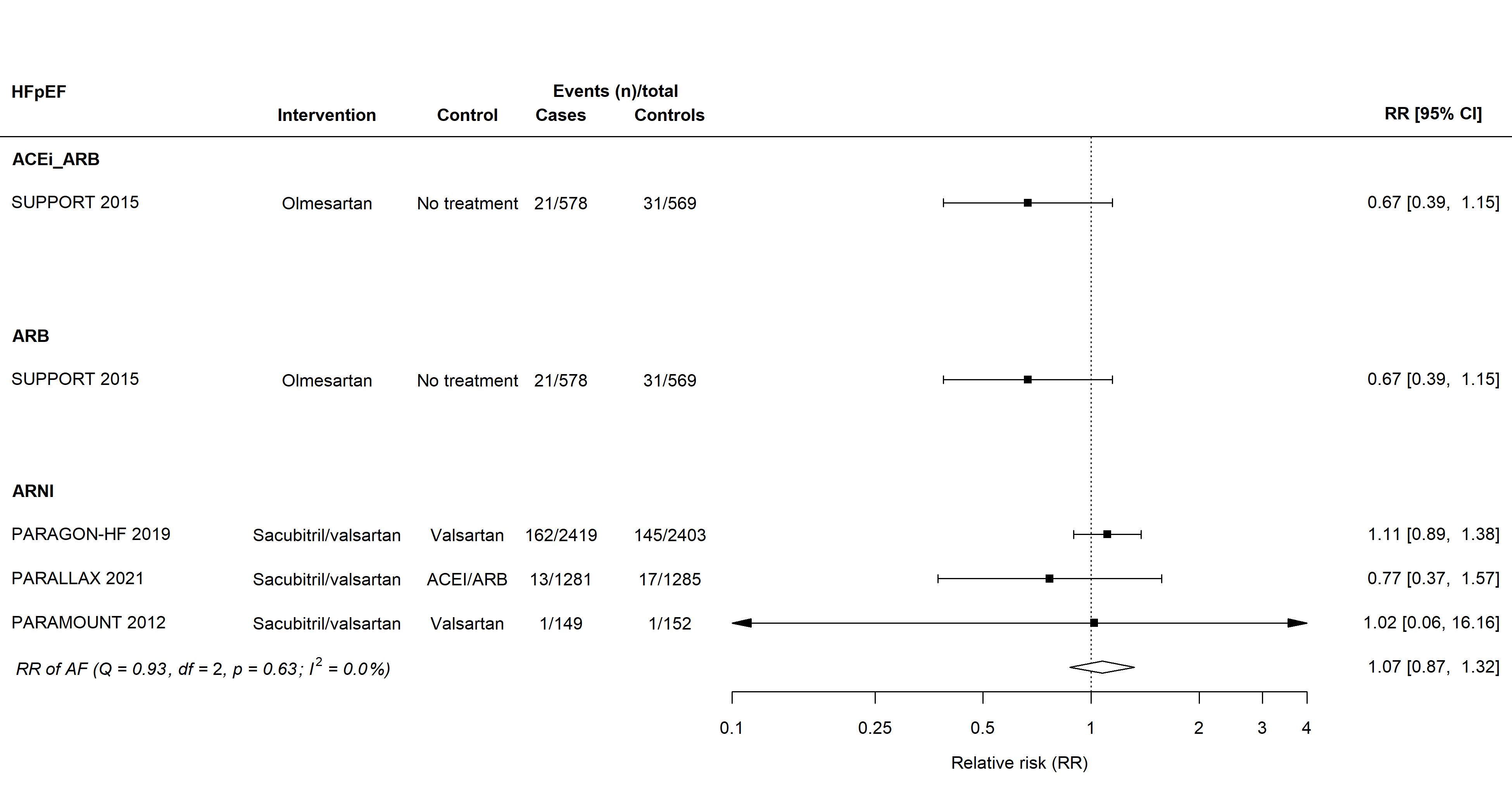


## Figure S6a: Association between pharmacotherapy and incident atrial fibrillation for any diabetes indication (ACEi, ARB, DPP4I), in placebo-controlled trials only.


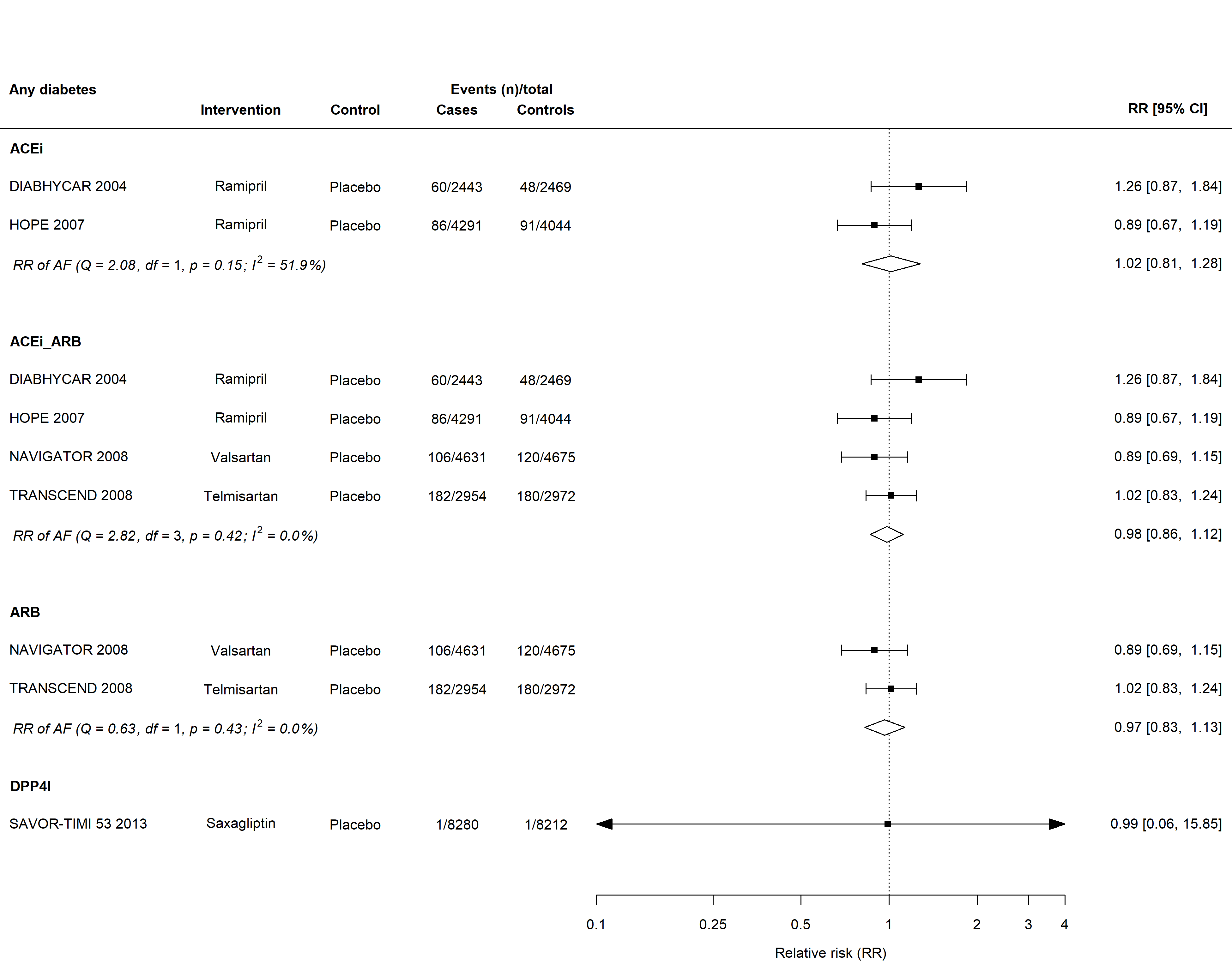


*Abbreviation: DPP4i, DPP-4 inhibitor*

## Figure S6b: Association between pharmacotherapy and incident atrial fibrillation for any diabetes indication (GLP-1 RA), in placebo-controlled trials only.


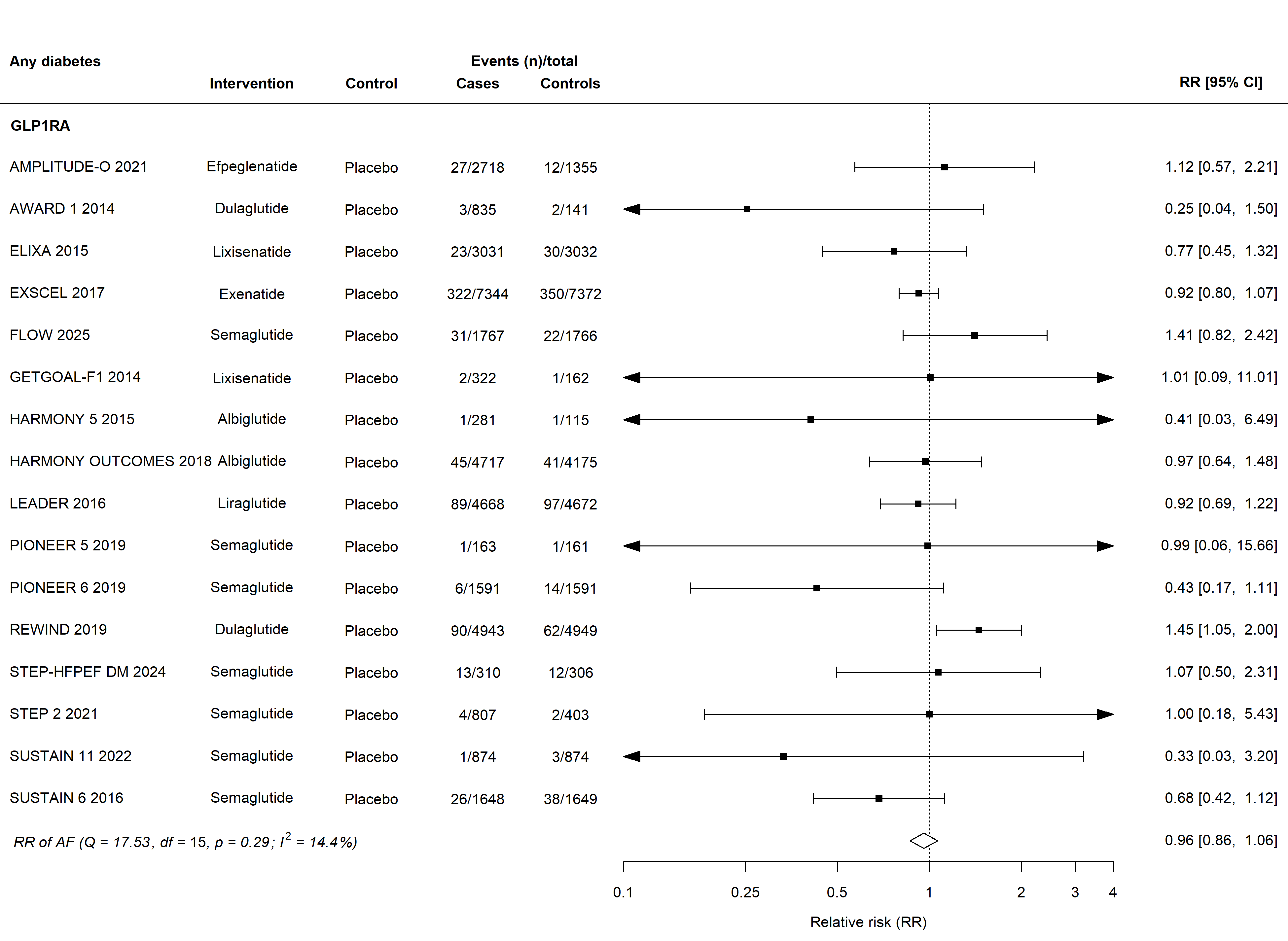


## Figure S6c: Association between pharmacotherapy and incident atrial fibrillation for any diabetes indication (MRA, SGLT2i, statin), in placebo-controlled trials only.


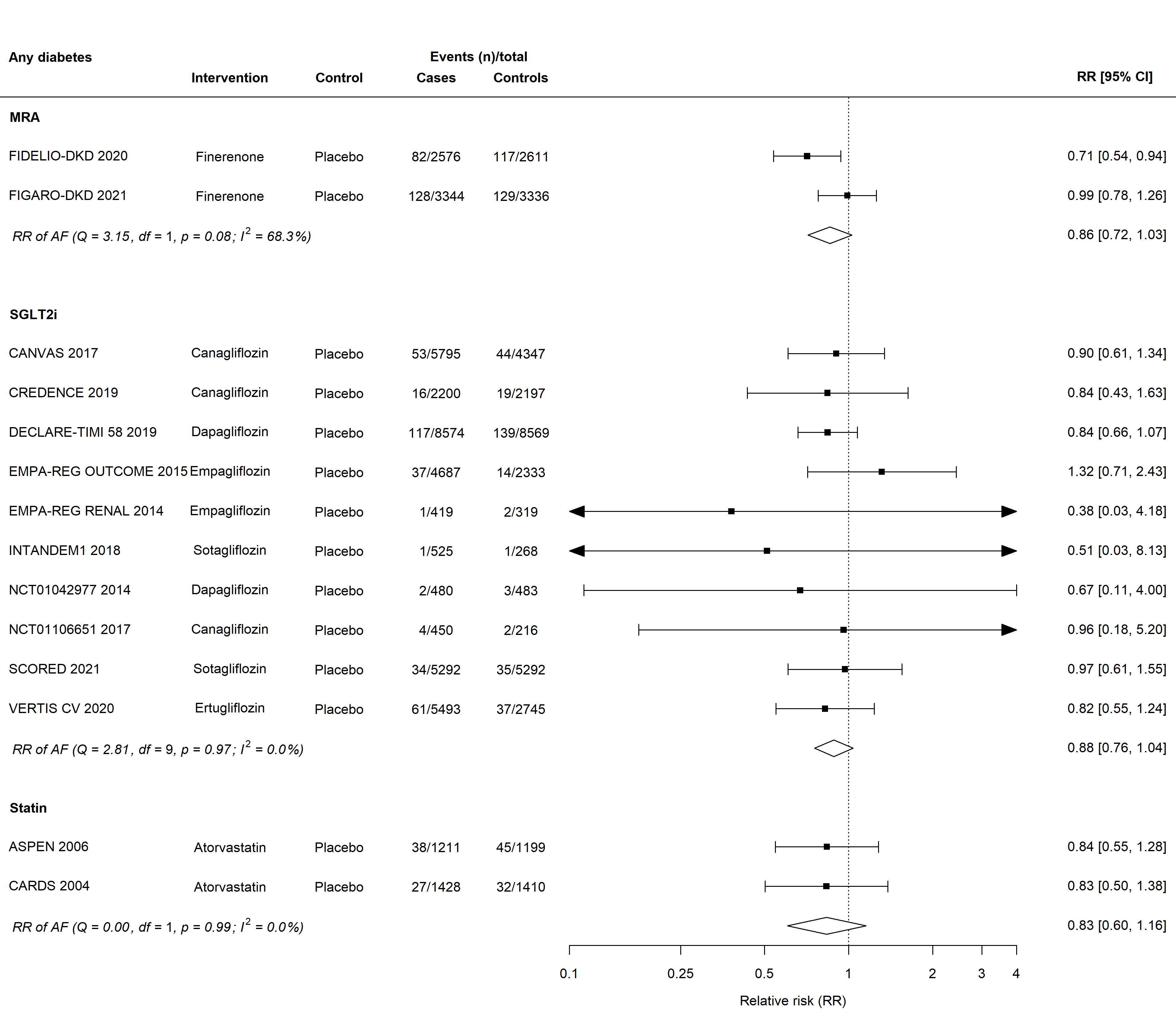


*Abbreviation: SGLT2i, SGLT2 Inhibitor*

## Figure S7: Association between pharmacotherapy and incident atrial fibrillation for diabetes mellitus indication, in placebo-controlled trials only.


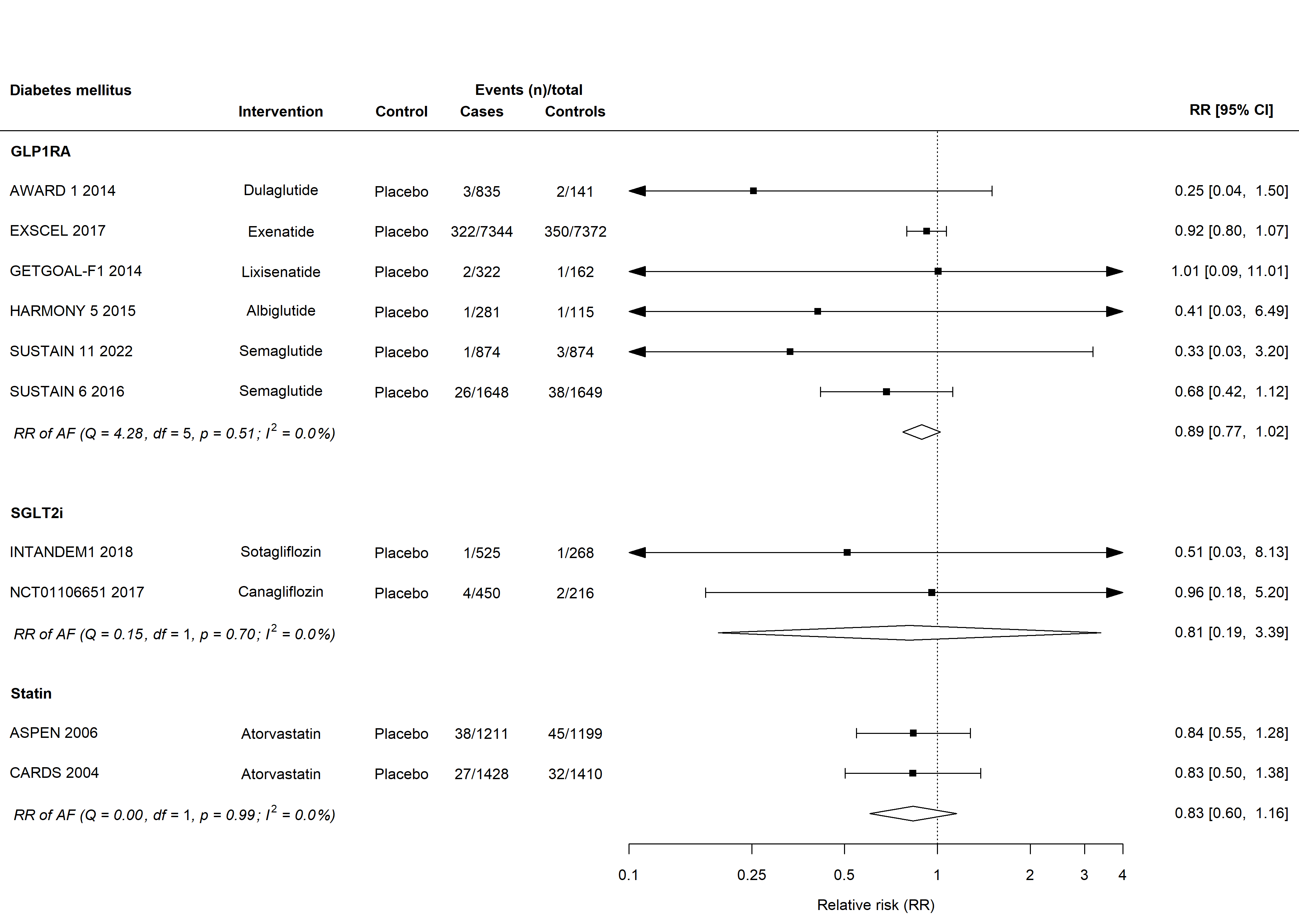


*Abbreviation: SGLT2i, SGLT2 Inhibitor*

## Figure S8: Association between pharmacotherapy and incident atrial fibrillation for diabetes with end target organ damage indication, in placebo-controlled trials only.


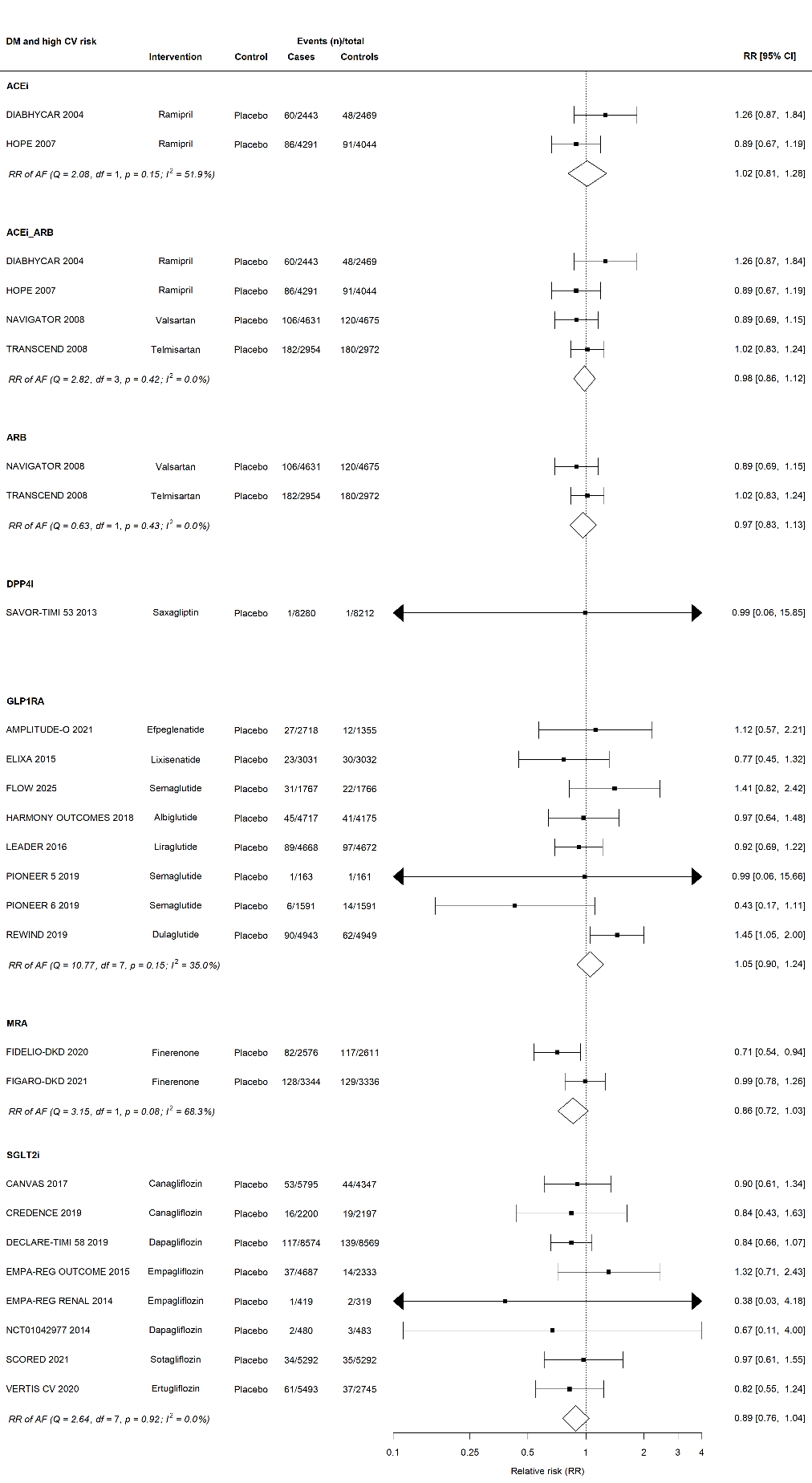


*Abbreviation: SGLT2i, SGLT2 Inhibitor*

## Figure S9a: Association between pharmacotherapy and incident atrial fibrillation for any diabetes indication (ARB, DPP4I), in non-placebo-controlled trials only.


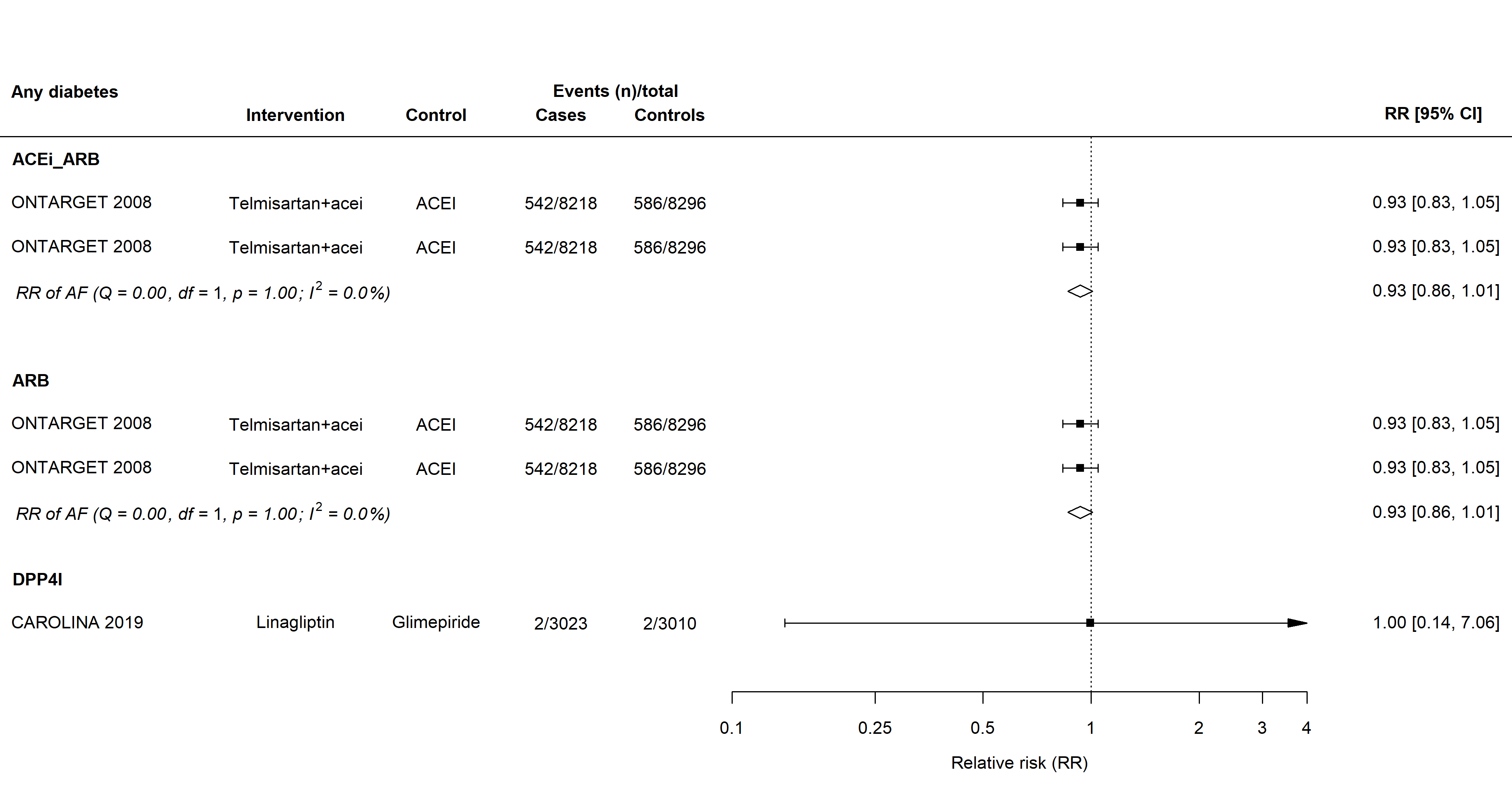

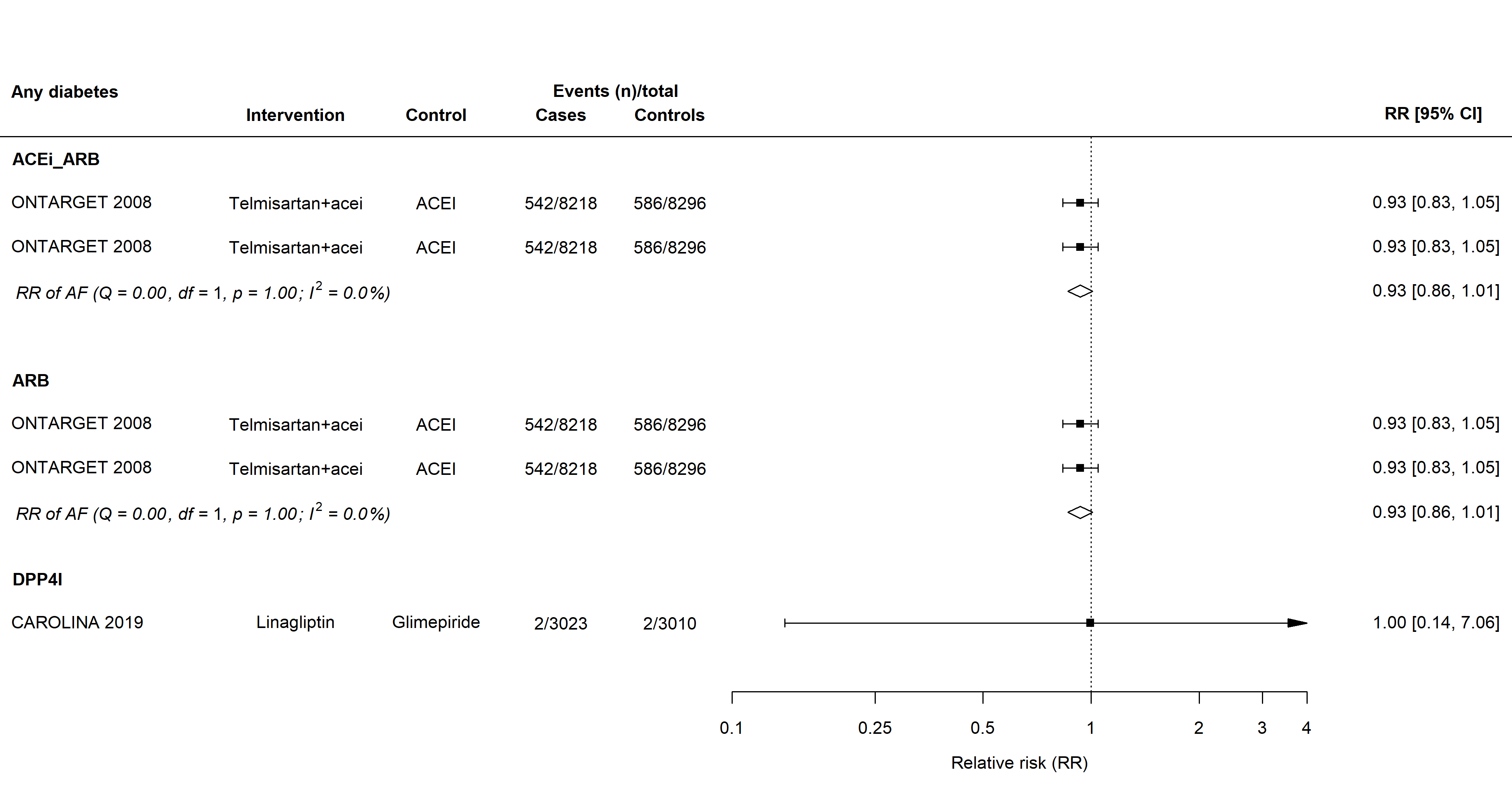

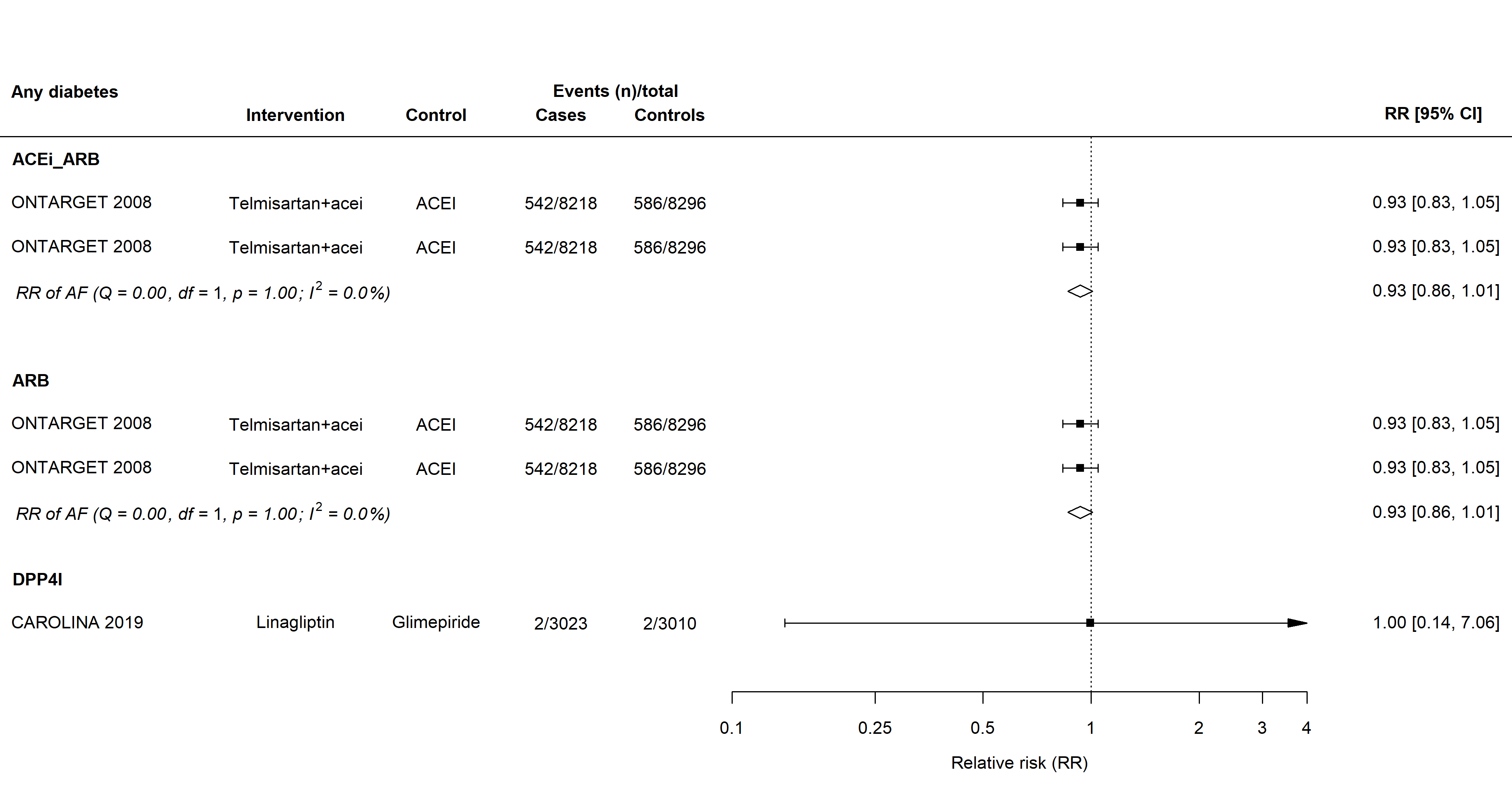


## Figure S9b: Association between pharmacotherapy and incident atrial fibrillation for any diabetes indication (GLP-1 RA), in non-placebo-controlled trials only.


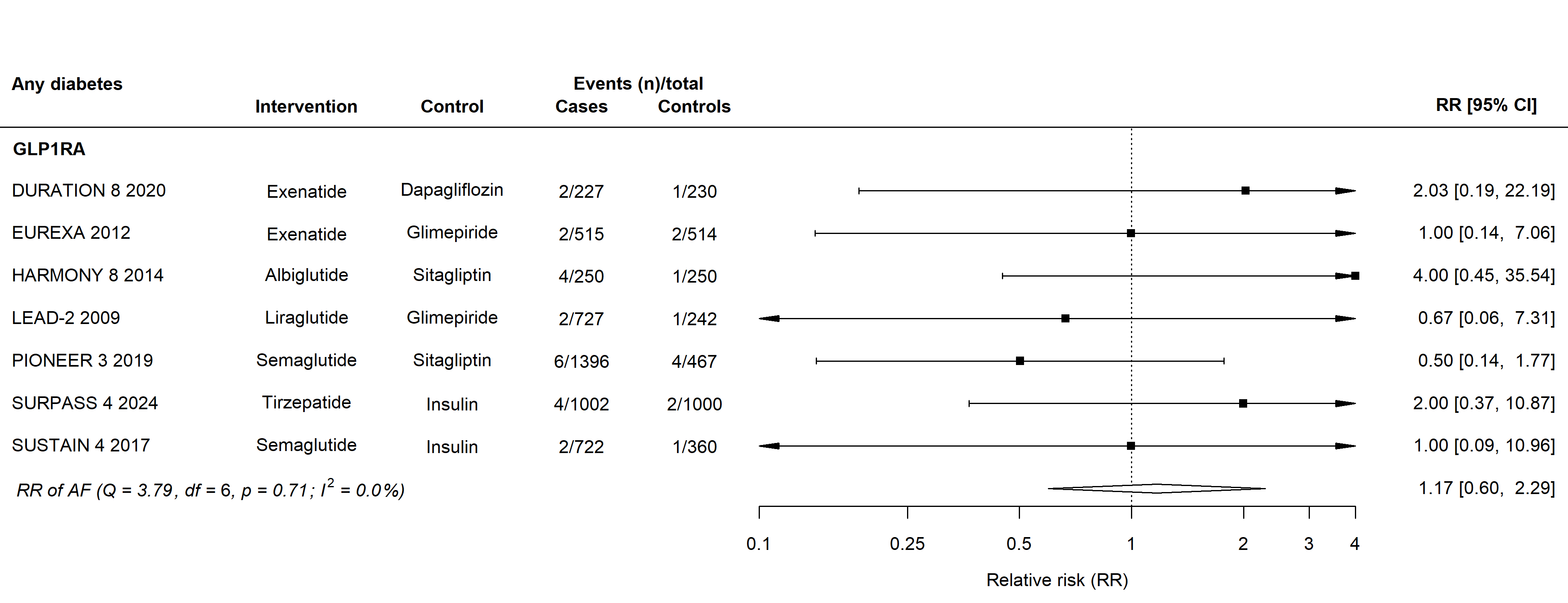


## Figure S9c: Association between pharmacotherapy and incident atrial fibrillation any diabetes indication (SGLT2i), in non-placebo-controlled trials only.


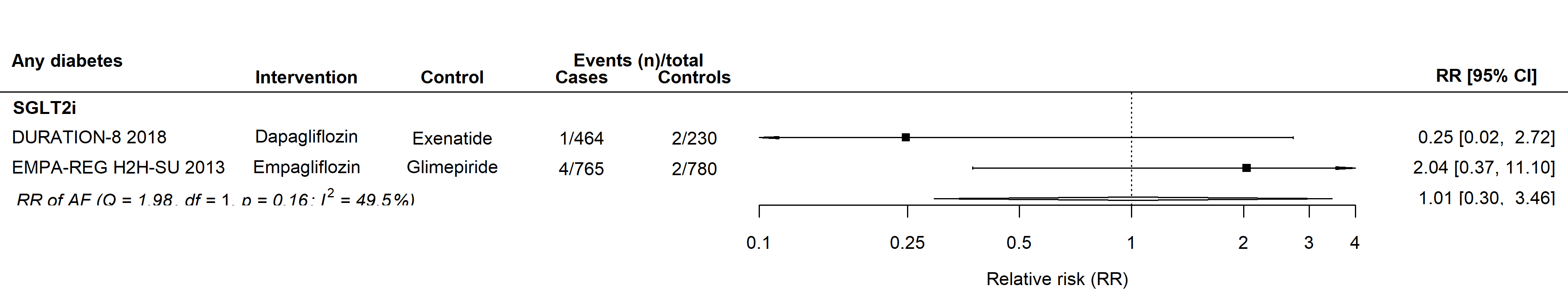


*Abbreviation: SGLT2i, SGLT2 Inhibitor*

## Figure S10: Association between pharmacotherapy and incident atrial fibrillation for diabetes mellitus indication, in non-placebo-controlled trials only.


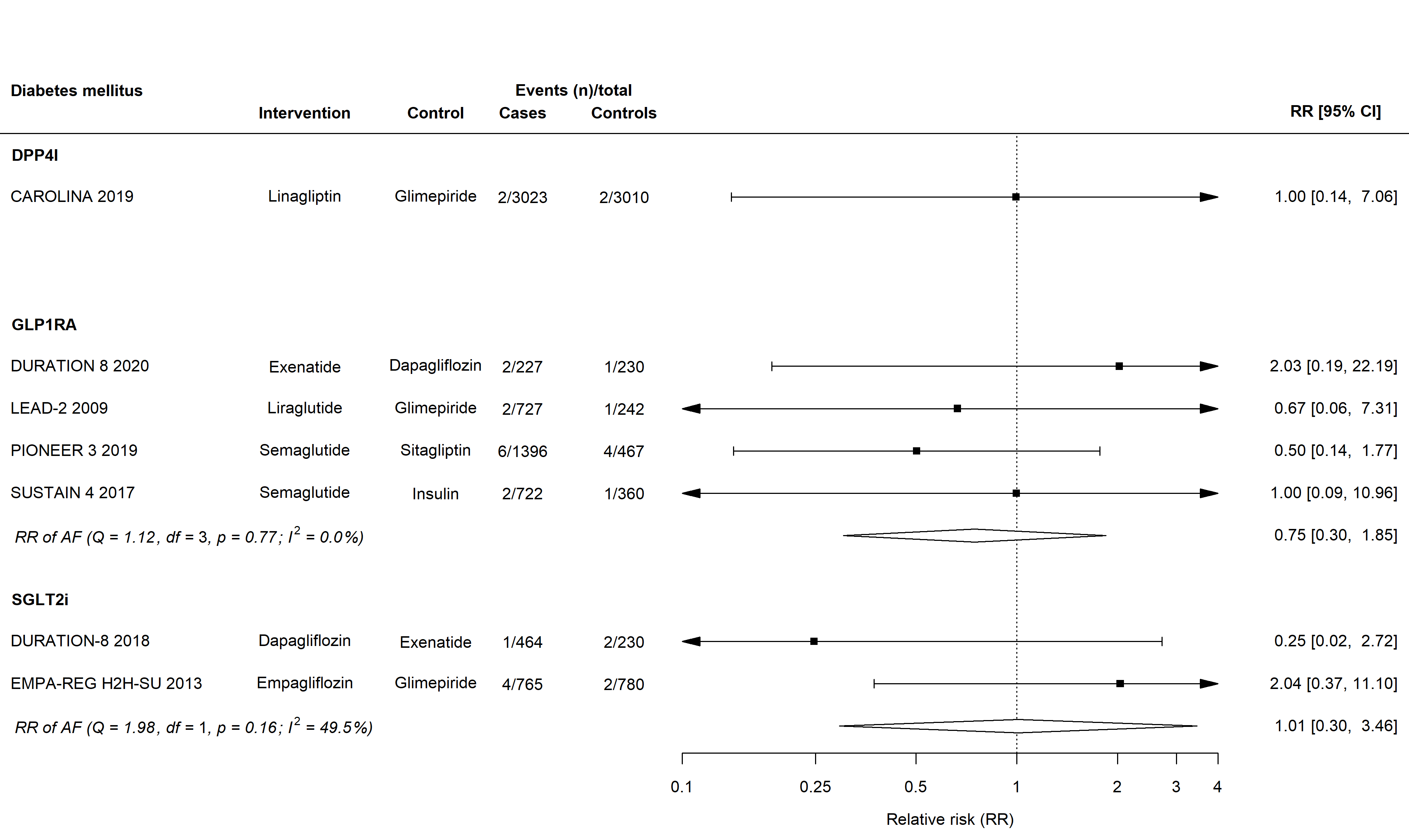


Figure S11: Association between pharmacotherapy and incident atrial fibrillation for diabetes with end target organ damage indication, in non-placebo-controlled trials only.


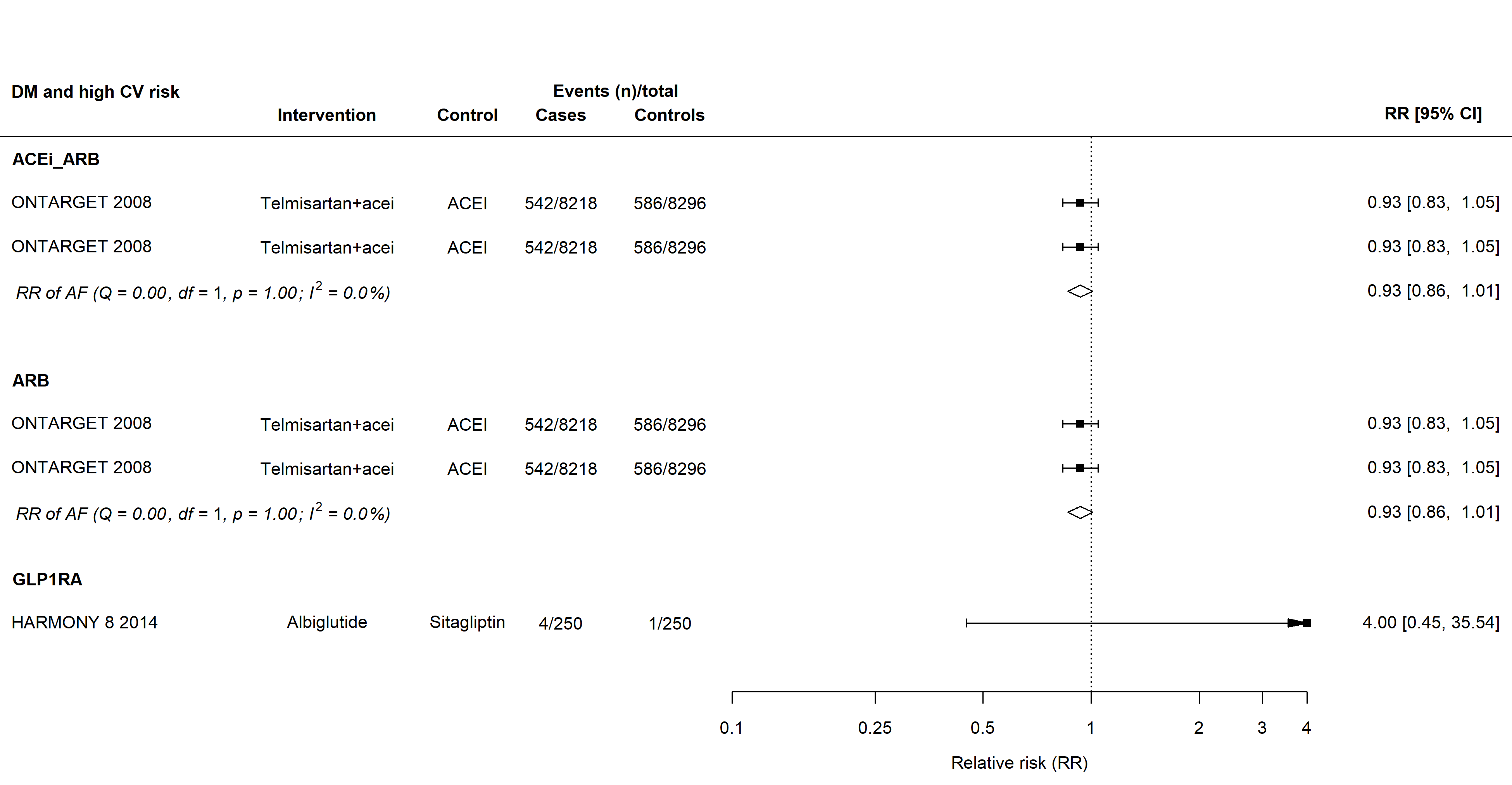

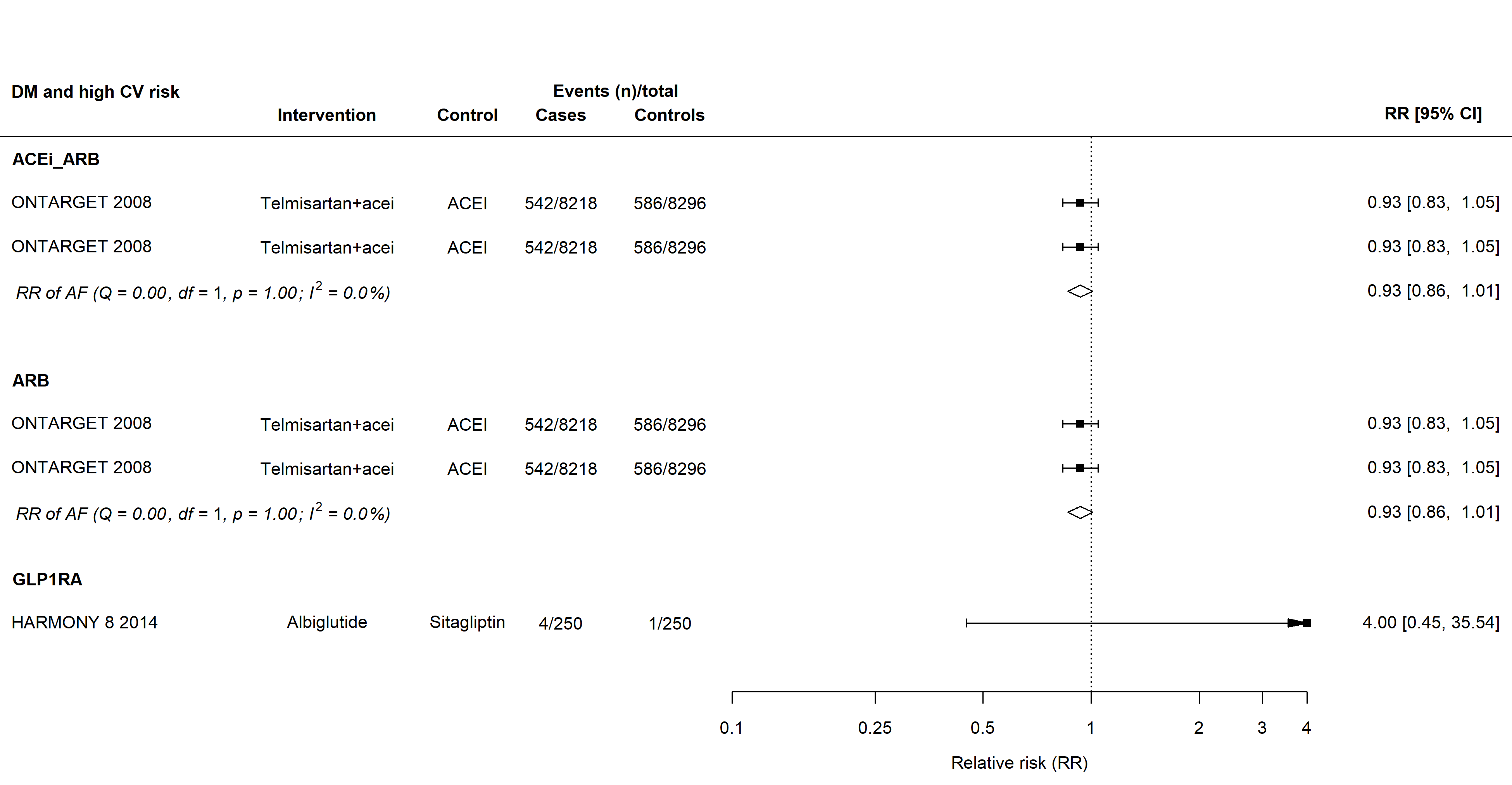

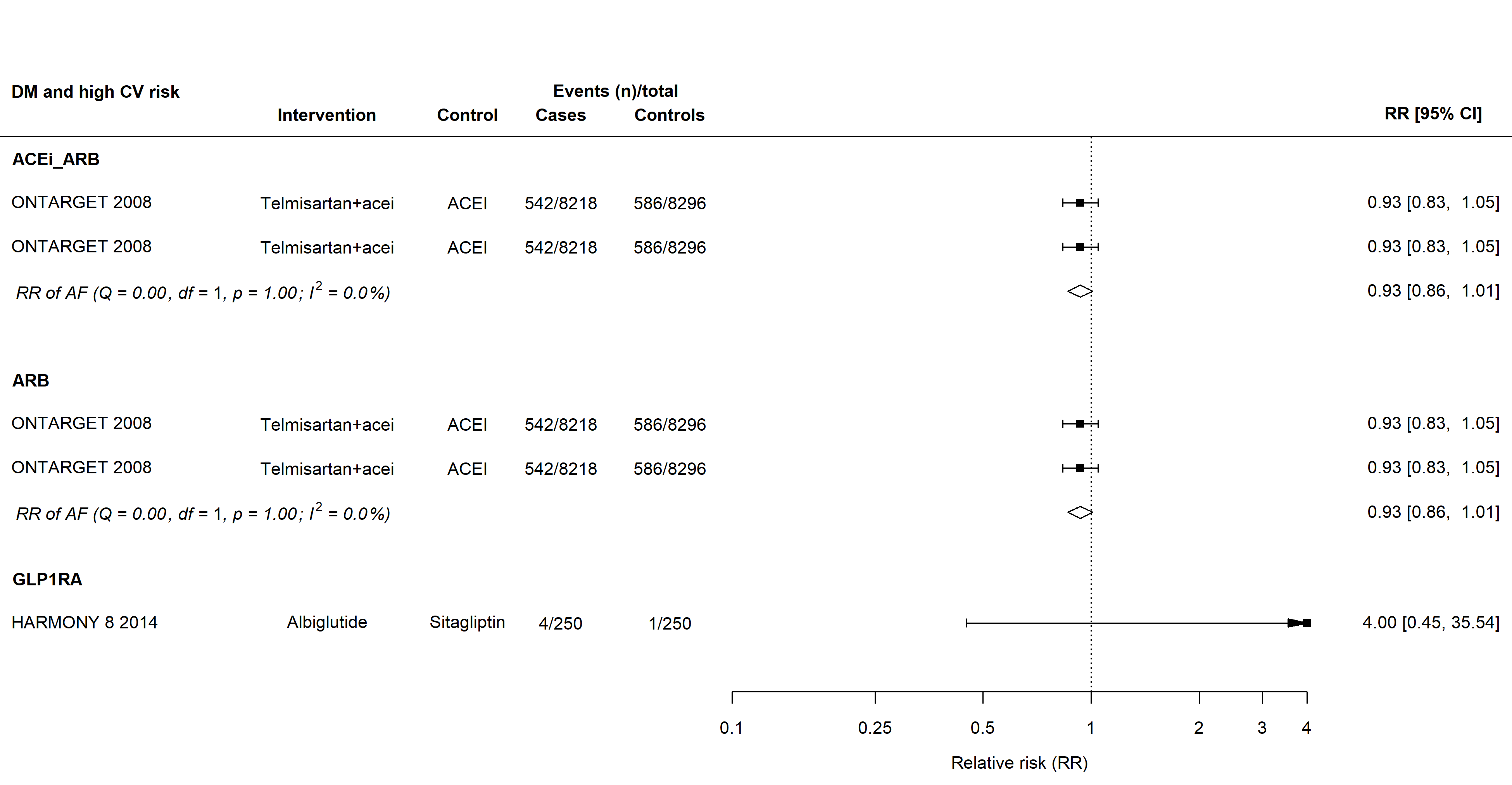


## Figure S12: Association between pharmacotherapy and incident atrial fibrillation for vascular disease indication, in placebo-controlled trials only.


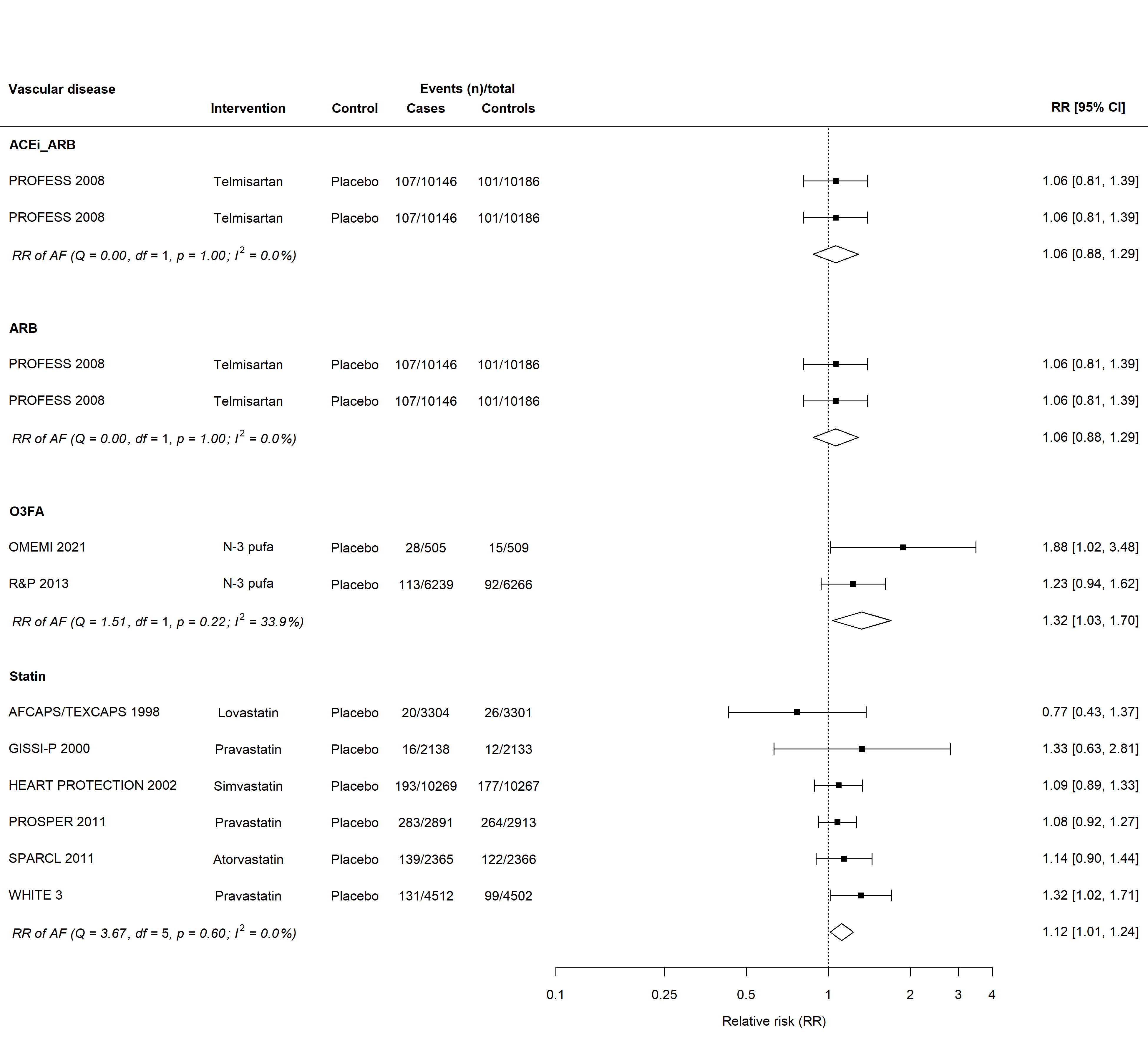

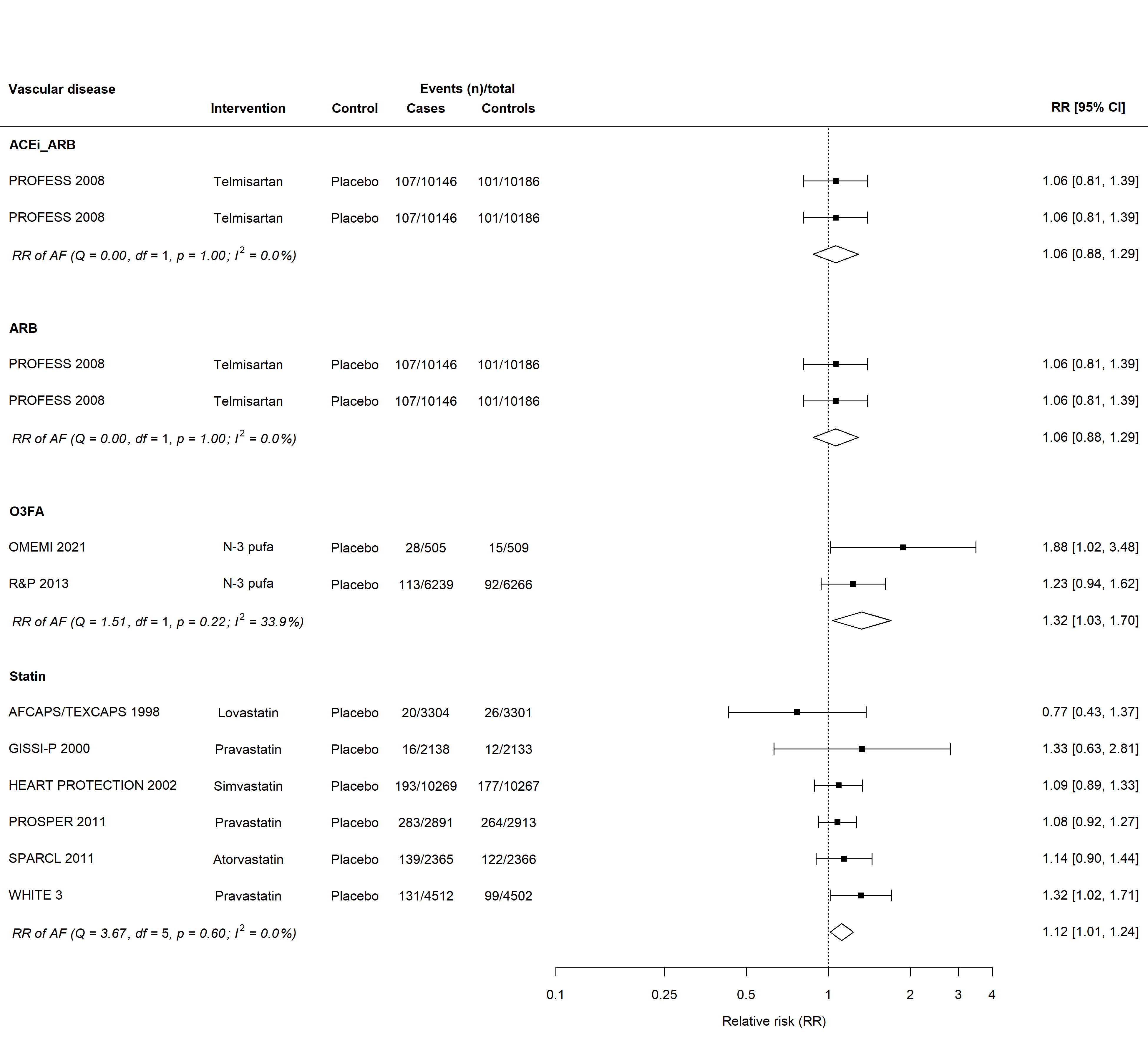

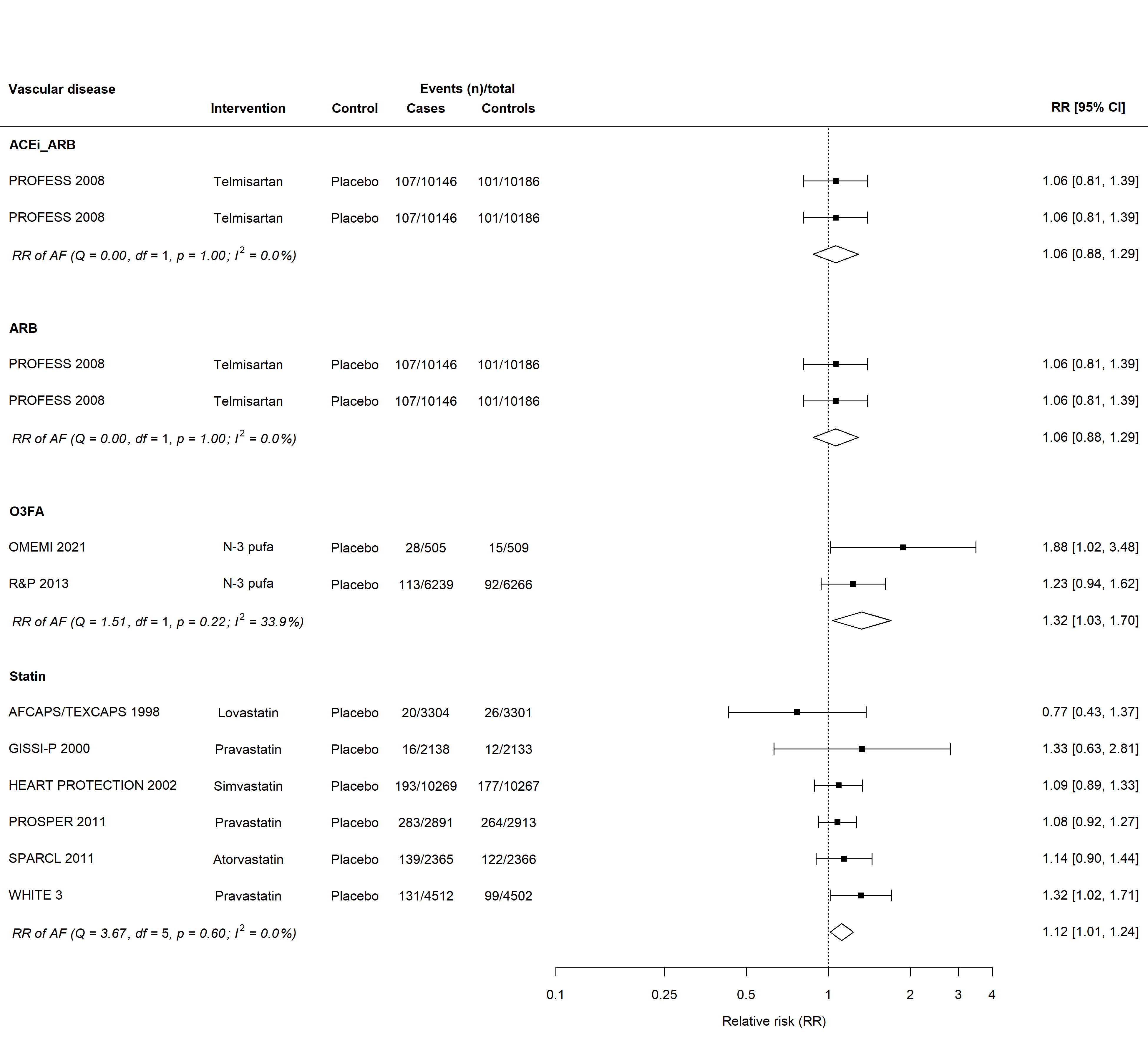


## Figure S13: Association between pharmacotherapy and incident atrial fibrillation for vascular disease indication, in non-placebo-controlled trials only.


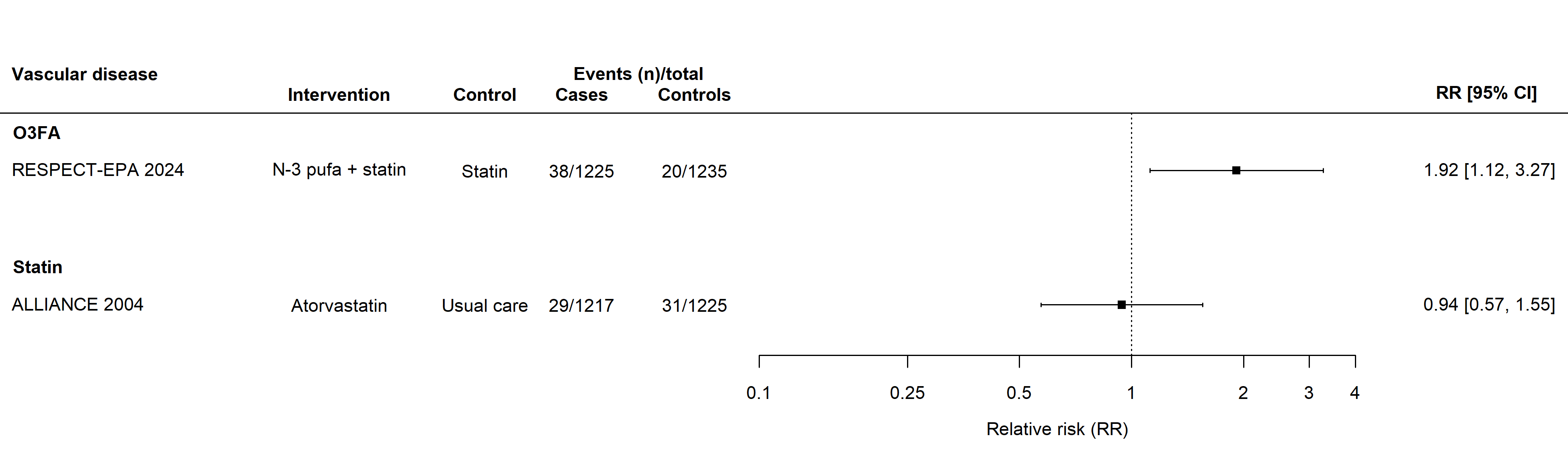


# Funnel plots of trials by relevant cardio-renal-metabolic indication.

## Figure S14: Funnel plot of diabetes trials.


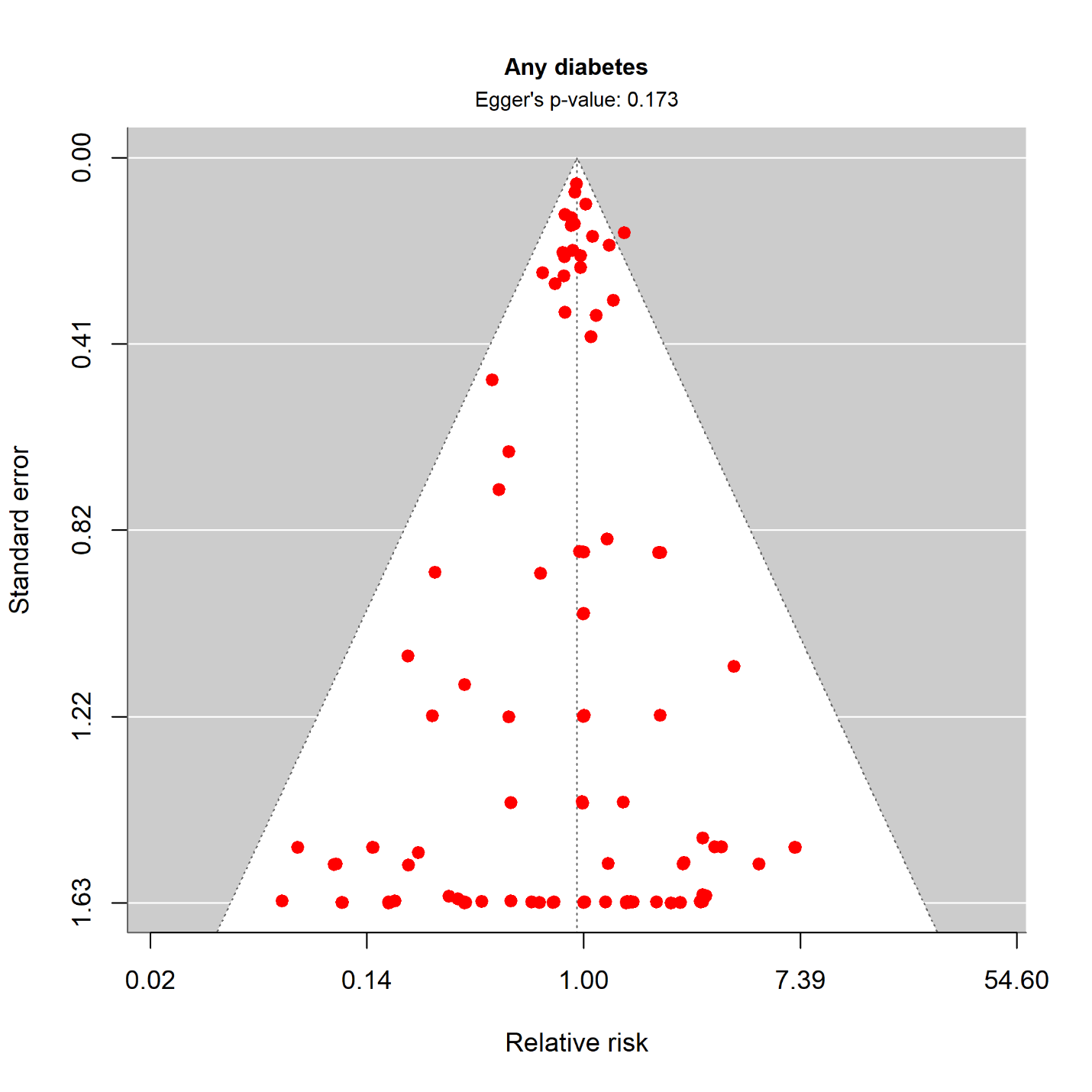


## Figure S15: Funnel plot of heart failure trials.


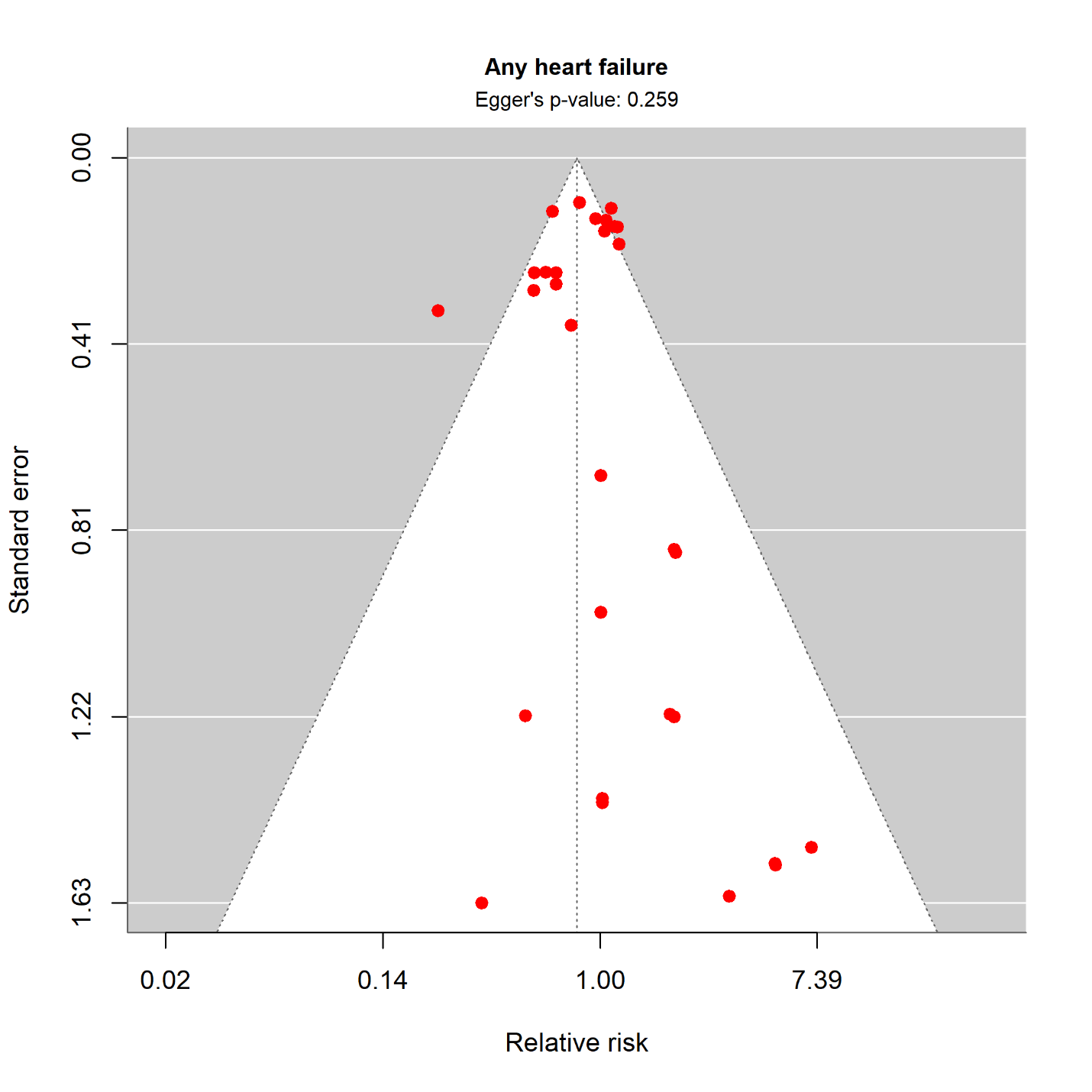


## Figure S16: Funnel plot of hypertension trials.


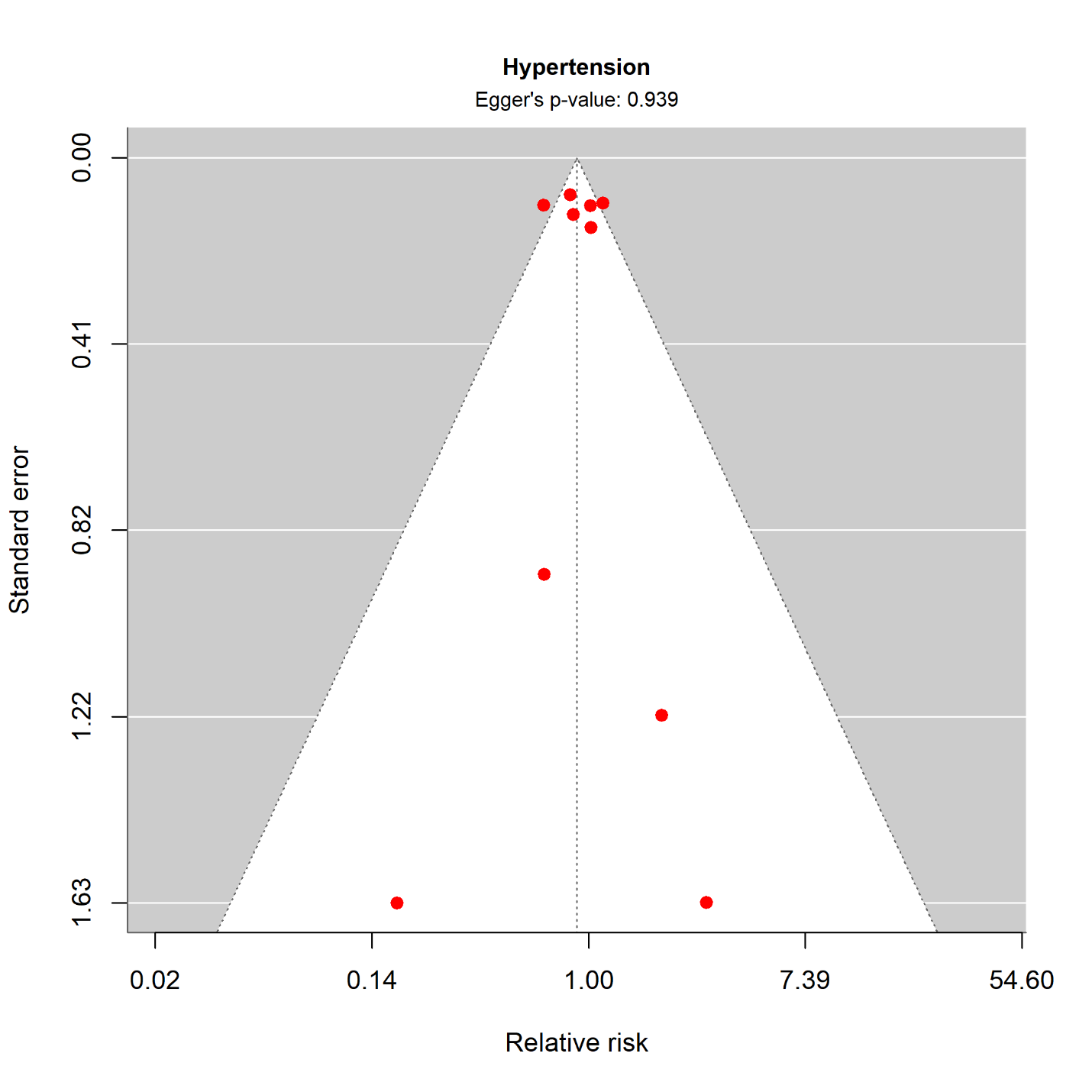


## Figure S17: Funnel plot of obesity trials.


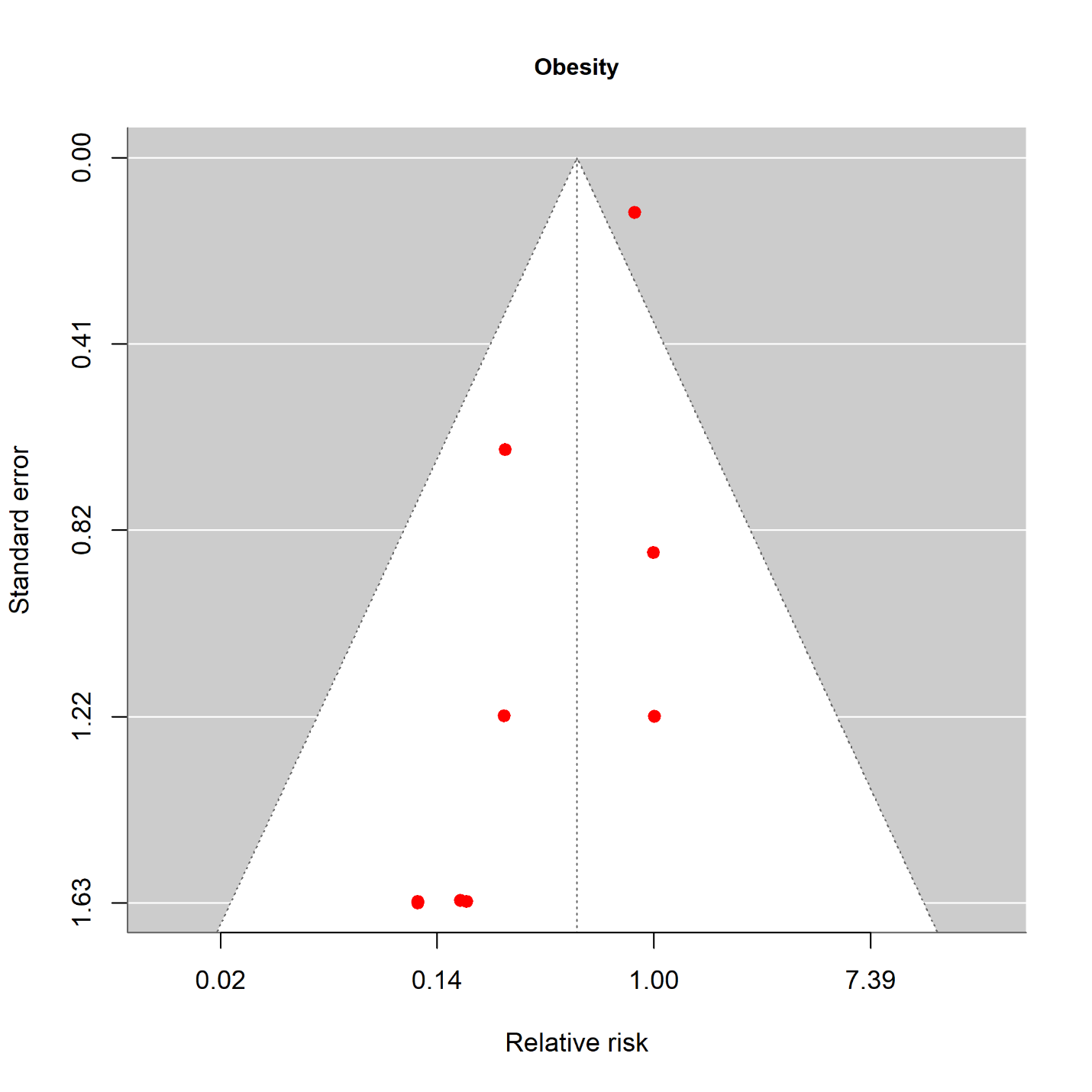


## Figure S18: Funnel plot of vascular disease trials.


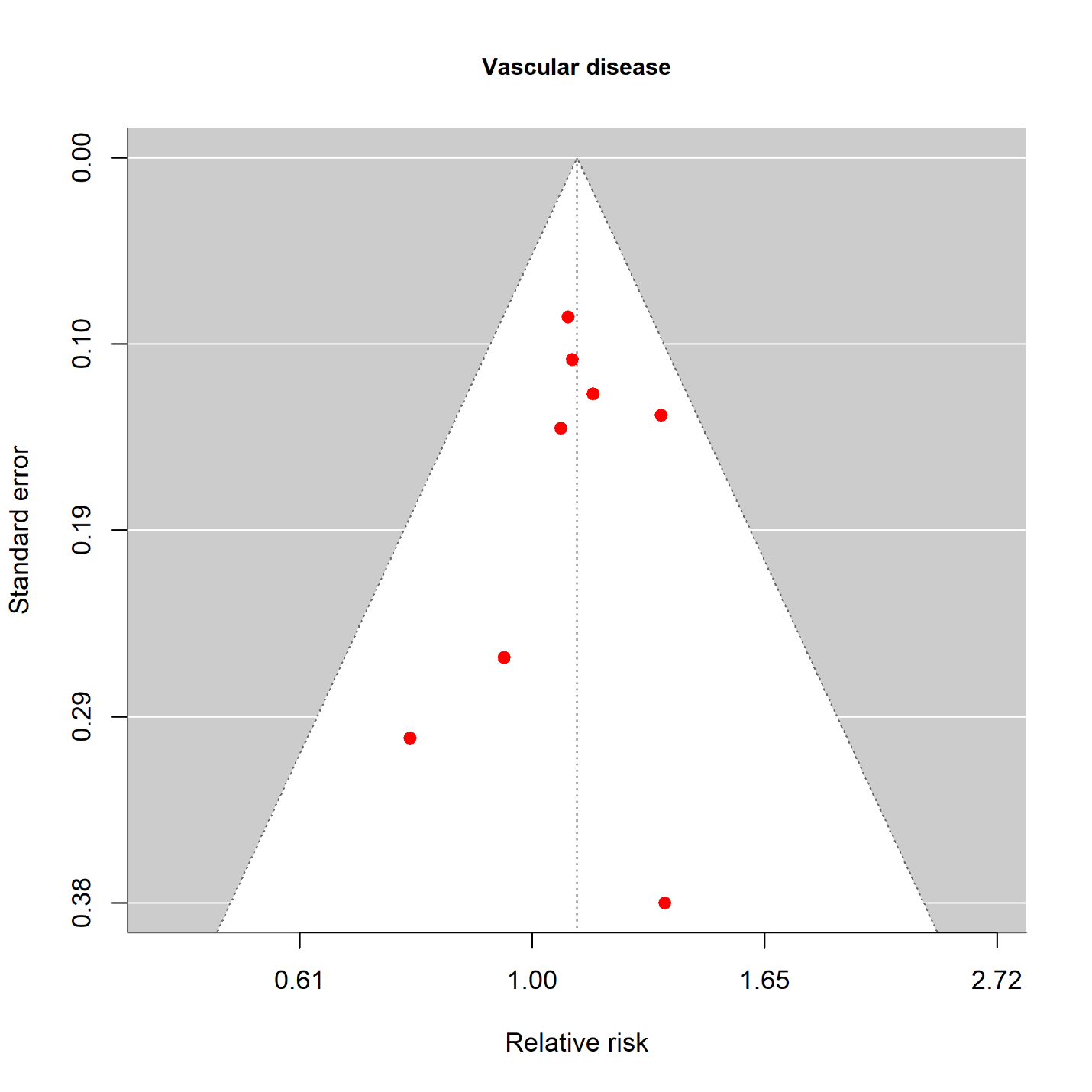


# Association between class of pharmacotherapy and incident atrial fibrillation in placebo-controlled trials of a single indication by individual agent.

## Figure S19: Association between MRAs and incident atrial fibrillation in placebo-controlled heart failure with reduced ejection fraction trials by agent.


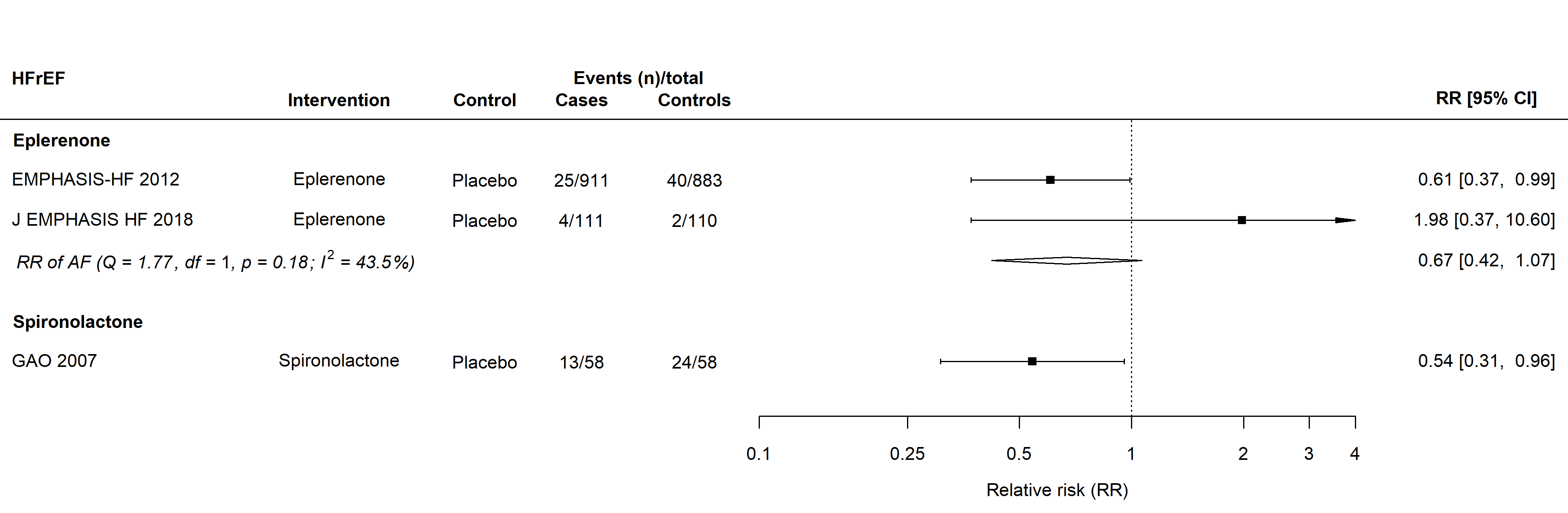


## Figure S20: Association between SGLT2is and incident atrial fibrillation in placebo-controlled heart failure with reduced ejection fraction trials by agent.


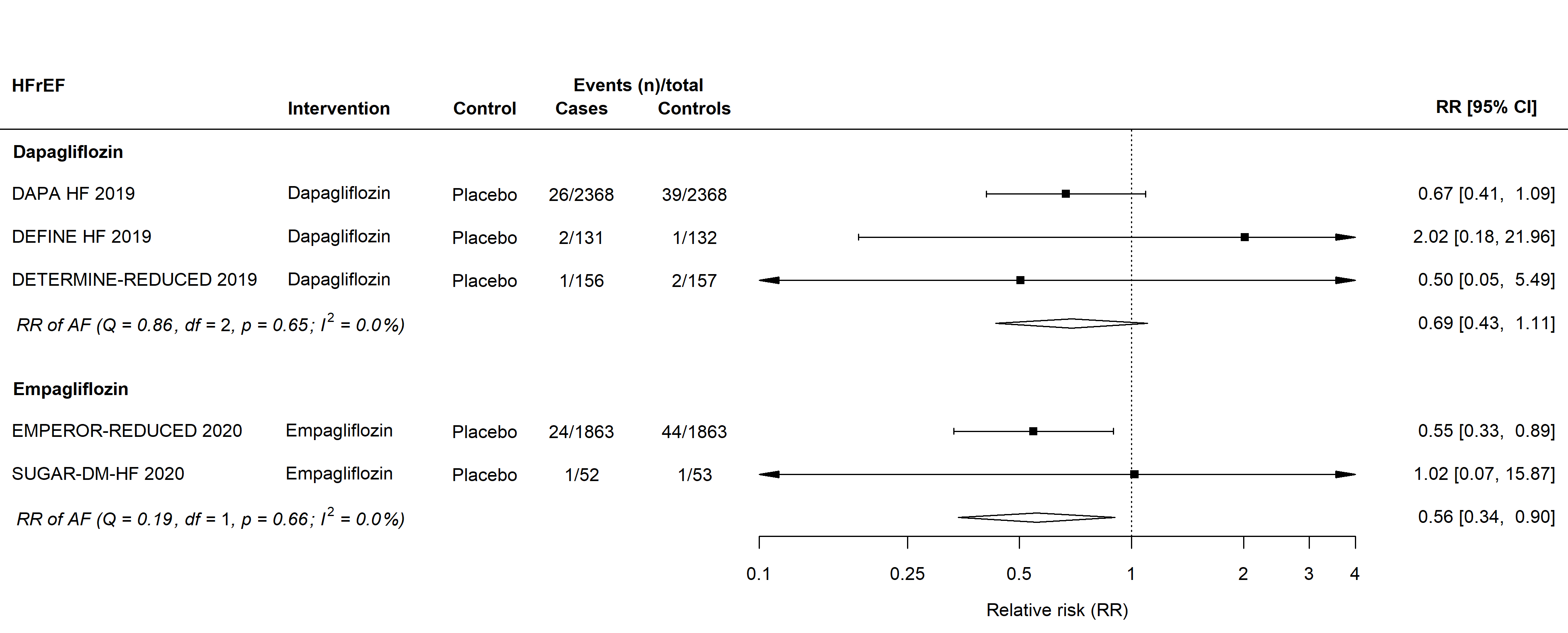


## Figure S21: Association between GLP-1 RA and incident atrial fibrillation in placebo-controlled obesity trials by agent.


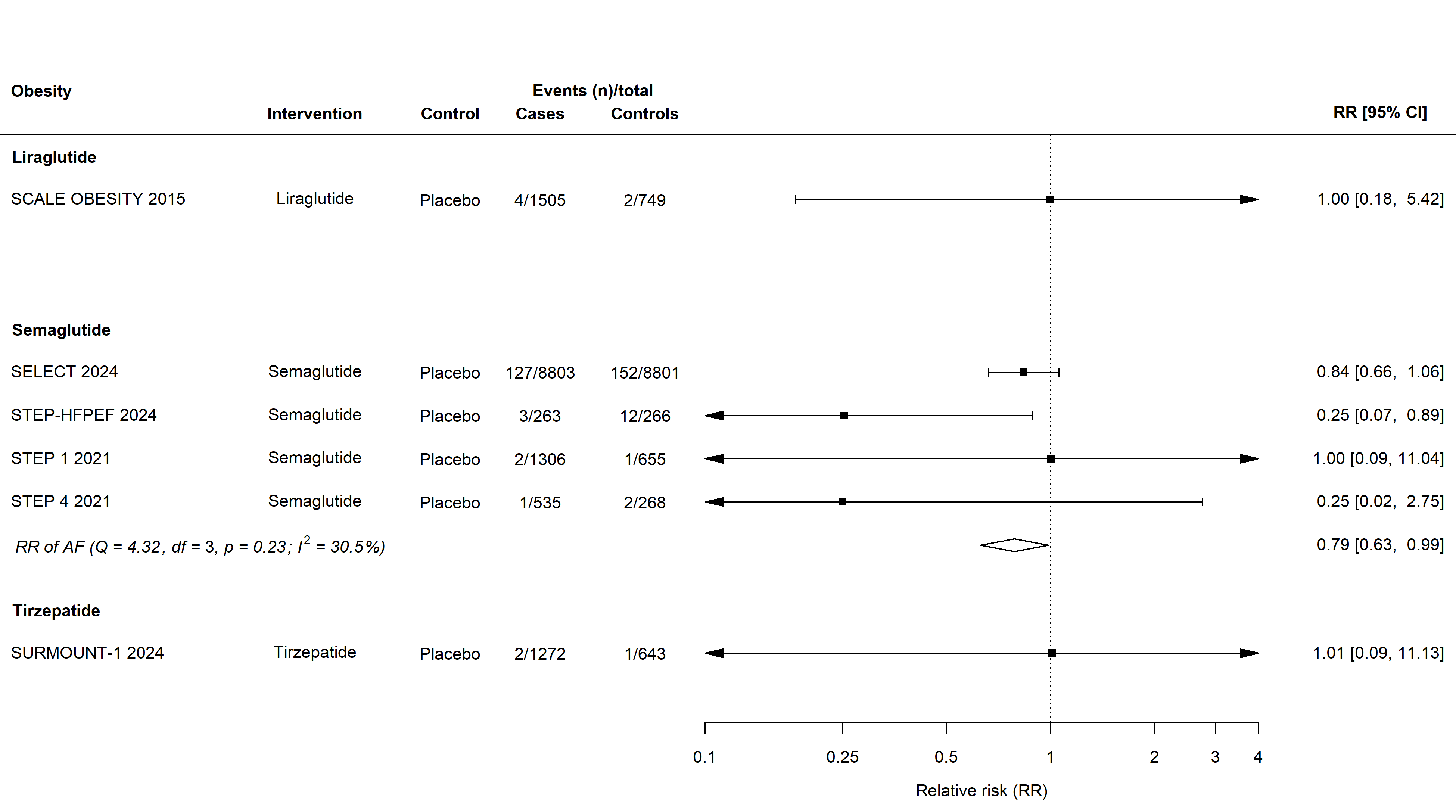


# Association between pharmacotherapy and incident atrial fibrillation in trials with prespecified endpoints.

## Figure S22: Association between pharmacotherapy in incident atrial fibrillation in trials with prespecified endpoints.


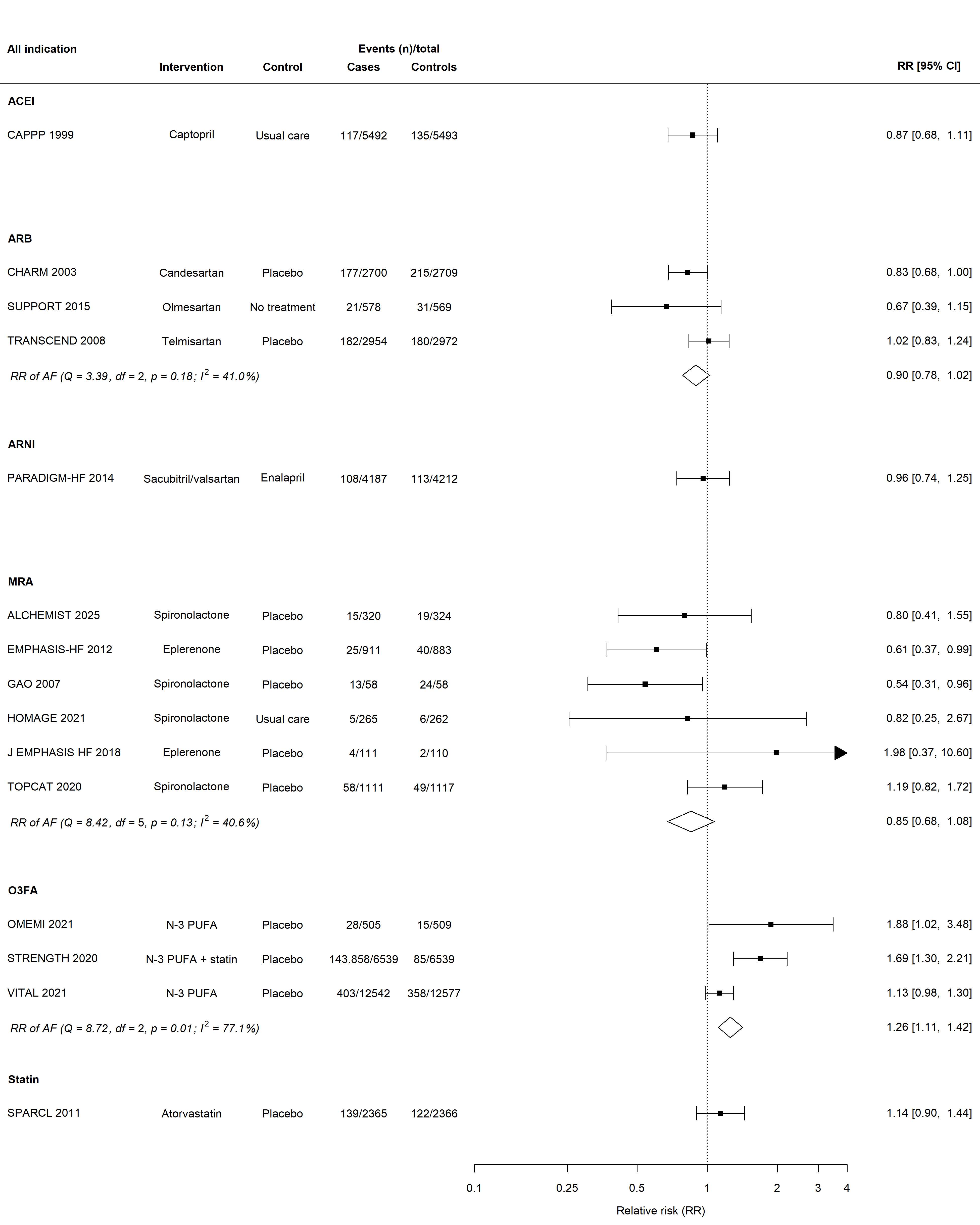


# Pharmacotherapies across cardio-renal-metabolic conditions

## Figure S23: Summary plot of overall risk of bias across all 249 trials.


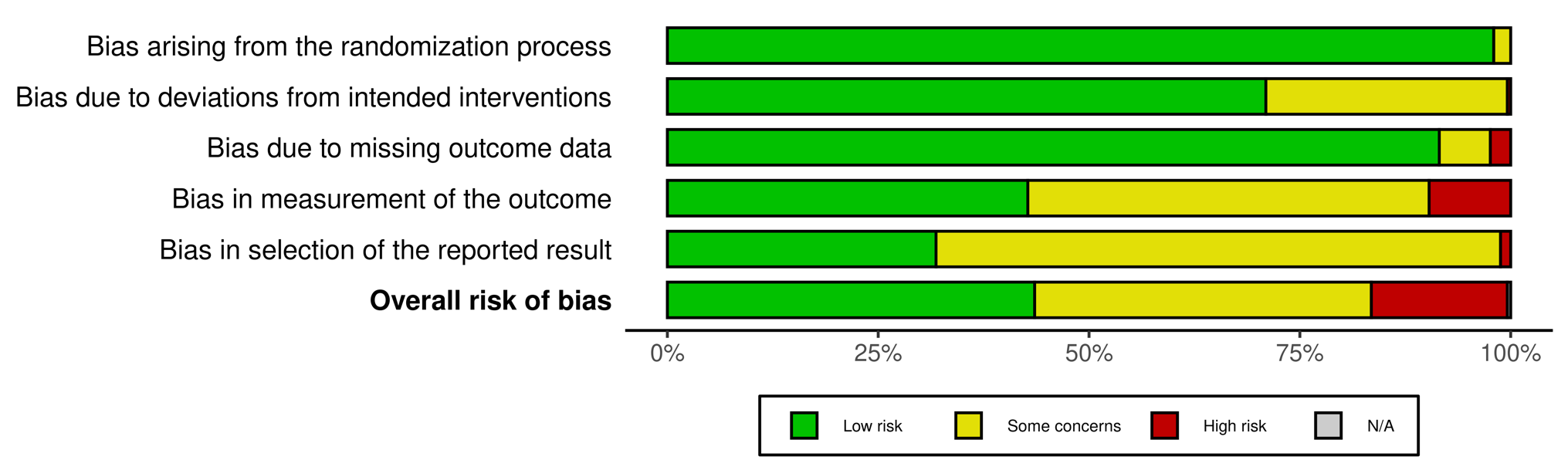

Supplement: ehag021_Supplementary_Data [file ehag021_supplementary_data.zip › NewSuppFigures20251019.docx]
