# Supplementary material for: Non-antiarrhythmic pharmacotherapy in cardio-renal-metabolic disease and incident atrial fibrillation: a trial meta-analysis
Source: Eur Heart J. 2026 Jan 28;47(23):2922–33. doi: 10.1093/eurheartj/ehag021 (PMC13268693; doi:10.1093/eurheartj/ehag021)
Supplement: ehag021_Supplementary_Data [file ehag021_supplementary_data.zip › Suppl. Material - Tables_updated_20251019.docx]

**Supplementary Material: Search Strategy, RCT Characteristics, Quality Assessment**

Non-antiarrhythmic pharmacotherapy in cardio-renal-metabolic disease and incident atrial fibrillation: a trial meta-analysis

Table of Contents

[Table S1: Search Strategy – Ovid 3](#_Toc211629915)

[Table S2: Search Strategy – Embase 4](#_Toc211629916)

[Table S3: Search Strategy – Cochrane 8](#_Toc211629917)

[Table S4: Indication of Trials by Class 9](#_Toc211629918)

[Table S5: Trial characteristics by pharmacotherapy class. 10](#_Toc211629919)

[Table S6: Trial Characteristics (ACEI) 11](#_Toc211629920)

[Table S7: Baseline Cohort Characteristics (ACEI) 12](#_Toc211629921)

[Table S8: Trial Characteristics (ARB) 13](#_Toc211629922)

[Table S10: Trial Characteristics (ARNI) 15](#_Toc211629923)

[Table S11: Baseline Cohort Characteristics (ARNI) 17](#_Toc211629924)

[Table S12: Trial Characteristics (DPP-4 Inhibitor) 18](#_Toc211629925)

[Table S13: Baseline Cohort Characteristics (DPP-4 Inhibitor) 19](#_Toc211629926)

[Table S14: Trial Characteristics (GLP-1 RA) 20](#_Toc211629927)

[Table S15: Baseline Cohort Characteristics (GLP-1 RA) 30](#_Toc211629928)

[Table S16: Trial Characteristics (MRA) 35](#_Toc211629929)

[Table S17: Baseline Cohort Characteristics (MRA) 37](#_Toc211629930)

[Table S18: Trial Characteristics (O3FA) 38](#_Toc211629931)

[Table S19: Baseline Cohort Characteristics (O3FA) 39](#_Toc211629932)

[Table S20: Trial Characteristics (SGLT2 Inhibitor) 40](#_Toc211629933)

[Table S21: Baseline Cohort Characteristics (SGLT2 Inhibitor) 46](#_Toc211629934)

[Table S22: Trial Characteristics (Statin) 51](#_Toc211629935)

[Table S23: Baseline Cohort Characteristics (Statin) 54](#_Toc211629936)

[Table S24: Risk of bias of ACEI trials. 56](#_Toc211629937)

[Table S25: Risk of bias of ARB trials. 57](#_Toc211629938)

[Table S26: Risk of bias of ARNI trials. 58](#_Toc211629939)

[Table S27: Risk of bias of DPP-4 Inhibitor trials. 59](#_Toc211629940)

[Table S28: Risk of bias of GLP-1 RA trials. 60](#_Toc211629941)

[Table S29: Risk of bias of MRA trials. 63](#_Toc211629942)

[Table S30: Risk of bias of O3FA trials. 64](#_Toc211629943)

[Table S31: Risk of bias of SGLT2 Inhibitor trials. 65](#_Toc211629944)

[Table S32: Risk of bias of statin trials. 68](#_Toc211629945)

[Table S33: Risk of bias in chronic kidney disease trials. 69](#_Toc211629946)

[Table S34: Risk of bias in all diabetes mellitus trials. 70](#_Toc211629947)

[Table S35: Risk of bias in heart failure with preserved ejection fraction trials. 72](#_Toc211629948)

[Table S36: Risk of bias in heart failure with reduced ejection fraction trials. 73](#_Toc211629949)

[Table S37: Risk of bias in hypertension trials. 74](#_Toc211629950)

[Table S38: Risk of bias in obesity trials. 75](#_Toc211629951)

[Table S39: Risk of bias in vascular disease trials. 76](#_Toc211629952)

[Table S40: Risk of bias in other trials. 77](#_Toc211629953)

[Table S41: Summary of Abbreviations in Suppl. File 1 78](#_Toc211629954)

[Table S42: PRISMA Checklist 79](#_Toc211629955)

[Table S43: PRISMA Abstract Checklist 83](#_Toc211629956)

#

# Table S1: Search Strategy – Ovid

Database(s): Ovid MEDLINE(R) ALL <1946 to October 7, 2025>

Search Strategy:

| # | Searches | Results |
| --- | --- | --- |
| 1 | Atrial fibrillation/ | 79258 |
| 2 | ((atrial or atria or atrium or auricular) adj3 fibrillat*).ti,ab. | 107170 |
| 3 | Af.ti,ab. | 62738 |
| 4 | or/1-3 | 140457 |
| 5 | Angiotensin Receptor Antagonists/ or Angiotensin-Converting Enzyme Inhibitors/ or Angiotensin II Type 1 Receptor Blockers/ | 49157 |
| 6 | (angiotensin converting enzyme inhibitors or ACE inhibitor or angiotensin receptor blockers or ARB).tw. | 29260 |
| 7 | bepridil/ or captopril/ or cilazapril/ or enalapril/ or fosinopril/ or irbesartan/ or lisinopril/ or losartan/ or perindopril/ or quinapril/ or ramipril/ or telmisartan/ or valsartan/ | 35534 |
| 8 | (bepridil or captopril or cilazapril or enalapril or fosinopril or irbesartan or lisinopril or losartan or perindopril or quinapril or ramipril or telmisartan or valsartan or candesartan or irbesartan or telmisartan).tw. | 45003 |
| 9 | mineralocorticoid receptor antagonists/ or canrenoic acid/ or canrenone/ or eplerenone/ or spironolactone/ | 11332 |
| 10 | (MRA or anti-aldosterone or aldosterone receptor antagonists or mineralocorticoid receptor antagonist or finerenone or spironolactone or eplerenone).tw. | 20032 |
| 11 | (Angiotensin receptor neprilysin inhibitor or neprilysin inhibitor or entresto or sacubitril valsartan or sacubitril).tw. | 2764 |
| 12 | sodium-glucose transporter 2 inhibitors/ or canagliflozin/ | 9004 |
| 13 | (sodium-glucose co-transporter or SGLT* or empagliflozin or dapagliflozin or canagliflozin or ertugliflozin).tw. | 18057 |
| 14 | exenatide/ or liraglutide/ | 5759 |
| 15 | (glucagon like peptide 1 receptor agonist or GLP* or exenatide or liraglutide or lixisenatide or dulaglutide or semaglutide).tw. | 31897 |
| 16 | dipeptidyl-peptidase iv inhibitors/ or linagliptin/ or sitagliptin phosphate/ or vildagliptin/ | 7519 |
| 17 | (DPP-4 inhibitors or DPP-4* or gliptins or sitagliptin or linagliptin or vildagliptin or saxagliptin or alogliptin or linagliptin).tw. | 8822 |
| 18 | hydroxymethylglutaryl-coa reductase inhibitors/ or atorvastatin/ or lovastatin/ or pravastatin/ or rosuvastatin calcium/ or simvastatin/ | 49606 |
| 19 | (hydroxymethylglutaryl-co A reductase inhibitor or HMG-coA* or statin or Fluvastatin or pravastatin or lovastatin or simvastatin or atorvastatin or rosuvastatin).tw. | 58035 |
| 20 | Fatty Acids, Omega-3/ | 17462 |
| 21 | (Omega-3 Fatty Acid or Omega 3 Fatty Acid or n-3 Oil or n-3 Fatty Acids or Omega 3 Fatty Acids or n-3 PUFA or n3 Fatty Acid or n3 Polyunsaturated Fatty Acid or n-3 Oils or N-3 Fatty Acid or Fatty Acid, N-3 or n-3 Polyunsaturated Fatty Acid or n 3 Polyunsaturated Fatty Acid or Oil, n-3 or Omega 3 Fatty Acids or PUFA, n3).tw. | 20869 |
| 22 | or/5-21 | 259772 |
| 23 | exp randomized controlled trial/ | 649351 |
| 24 | controlled clinical trial.pt. | 95743 |
| 25 | randomized.ab. | 712470 |
| 26 | placebo.ab. | 262702 |
| 27 | drug therapy.fs. | 2859743 |
| 28 | randomly.ab. | 470787 |
| 29 | trial.ab. | 778705 |
| 30 | groups.ab. | 2926811 |
| 31 | or/23-30 | 6429795 |
| 32 | 4 and 22 and 31 | 2412 |

#

# Table S2: Search Strategy – Embase

Database(s): Embase <1947 to 2025 Week 41>

Search Strategy:

| # | Searches | Results |
| --- | --- | --- |
| 1 | atrial fibrillation/ | 248477 |
| 2 | heart atrium fibrillation/ | 248477 |
| 3 | ((atrial or atria or atrium or auricular) adj3 fibrillat*).ti,ab. | 201417 |
| 4 | AF.ti,ab. | 122158 |
| 5 | or/1-4 | 310868 |
| 6 | angiotensin antagonist/ or angiotensin/ or angiotensin 2 receptor antagonist/ or angiotensin II antagonist/ or angiotensin receptor antagonist/ or dipeptidyl carboxypeptidase inhibitor/ | 202834 |
| 7 | (angiotensin converting enzyme inhibitors or ACE inhibitor or angiotensin receptor blockers or ARB).tw. | 46322 |
| 8 | bepridil/ or captopril/ or cilazapril/ or enalapril/ or fosinopril/ or irbesartan/ or lisinopril/ or losartan/ or perindopril/ or quinapril/ or ramipril/ or telmisartan/ or valsartan/ | 133531 |
| 9 | (bepridil or captopril or cilazapril or enalapril or fosinopril or irbesartan or lisinopril or losartan or perindopril or quinapril or ramipril or telmisartan or valsartan or candesartan or irbesartan or telmisartan).tw. | 69885 |
| 10 | mineralocorticoid receptor antagonists/ or canrenoic acid/ or canrenone/ or eplerenone/ or spironolactone/ | 55318 |
| 11 | (MRA or anti-aldosterone or aldosterone receptor antagonists or mineralocorticoid receptor antagonist or finerenone or spironolactone or eplerenone).tw. | 35963 |
| 12 | sacubitril plus valsartan/ | 7078 |
| 13 | (Angiotensin receptor neprilysin inhibitor or neprilysin inhibitor or entresto or sacubitril valsartan or sacubitril).tw. | 5557 |
| 14 | sodium glucose cotransporter 2 inhibitor/ or sodium glucose cotransporter inhibitor/ or atigliflozin/ or bexagliflozin/ or canagliflozin/ or dapagliflozin/ or empagliflozin/ or enavogliflozin/ or ertugliflozin/ or ipragliflozin/ or licogliflozin/ or luseogliflozin/ or mizagliflozin/ or remogliflozin etabonate/ or sergliflozin etabonate/ or sotagliflozin/ or tofogliflozin/ | 42028 |
| 15 | (sodium-glucose co-transporter or SGLT* or empagliflozin or dapagliflozin or canagliflozin or ertugliflozin).tw. | 31587 |
| 16 | glucagon like peptide 1 receptor agonist/ or glucagon like peptide receptor agonist/ or dulaglutide/ or exendin 4/ or liraglutide/ or lixisenatide/ or semaglutide/ | 44555 |
| 17 | (glucagon like peptide 1 receptor agonist or GLP* or exenatide or liraglutide or lixisenatide or dulaglutide or semaglutide).tw. | 55879 |
| 18 | dipeptidyl peptidase iv inhibitor/ or alogliptin/ or linagliptin/ or saxagliptin/ or sitagliptin/ or vildagliptin/ | 32310 |
| 19 | (DPP-4 inhibitors or DPP-4* or gliptins or sitagliptin or linagliptin or vildagliptin or saxagliptin or alogliptin or linagliptin).tw. | 16739 |
| 20 | hydroxymethylglutaryl coenzyme a reductase inhibitor/ or atorvastatin/ or cerivastatin/ or pitavastatin/ or pravastatin/ or rosuvastatin/ or simvastatin/ | 200500 |
| 21 | (hydroxymethylglutaryl-co A reductase inhibitor or HMG-coA* or statin or Fluvastatin or pravastatin or lovastatin or simvastatin or atorvastatin or rosuvastatin).tw. | 96784 |
| 22 | omega 3 fatty acid/ | 45198 |
| 23 | (Omega-3 Fatty Acid or Omega 3 Fatty Acid or n-3 Oil or n-3 Fatty Acids or Omega 3 Fatty Acids or n-3 PUFA or n3 Fatty Acid or n3 Polyunsaturated Fatty Acid or n-3 Oils or N-3 Fatty Acid or Fatty Acid, N-3 or n-3 Polyunsaturated Fatty Acid or n 3 Polyunsaturated Fatty Acid or Oil, n-3 or Omega 3 Fatty Acids or PUFA, n3).tw. | 27747 |
| 24 | or/6-23 | 683891 |
| 25 | exp randomized controlled trial/ | 1110605 |
| 26 | Controlled clinical trial/ | 460707 |
| 27 | random$.ti,ab. | 2515870 |
| 28 | randomization/ | 101772 |
| 29 | intermethod comparison/ | 317035 |
| 30 | placebo.ti,ab. | 470293 |
| 31 | (compare or compared or comparison).ti. | 729119 |
| 32 | ((evaluated or evaluate or evaluating or assessed or assess) and (compare or compared or comparing or comparison)).ab. | 3363569 |
| 33 | (open adj label).ti,ab. | 187963 |
| 34 | ((double or single or doubly or singly) adj (blind or blinded or blindly)).ti,ab. | 374113 |
| 35 | double blind procedure/ | 312110 |
| 36 | parallel group$1.ti,ab. | 53006 |
| 37 | (crossover or cross over).ti,ab. | 158142 |
| 38 | ((assign$ or match or matched or allocation) adj5 (alternate or group$1 or intervention$1 or patient$1 or subject$1 or participant$1)).ti,ab. | 515540 |
| 39 | (assigned or allocated).ti,ab. | 612979 |
| 40 | (controlled adj7 (study or design or trial)).ti,ab. | 640401 |
| 41 | (volunteer or volunteers).ti,ab. | 329666 |
| 42 | Human experiment/ | 730961 |
| 43 | Trial.ti. | 573309 |
| 44 | or/25-43 | 7627384 |
| 45 | (random$ adj sampl$ adj7 ("cross section$" or questionnaire$1 or survey$ or database$1)).ti,ab. not (comparative study/ or controlled study/ or randomi?ed controlled.ti,ab. or randomly assigned.ti,ab.) | 10847 |
| 46 | Cross-sectional study/ not (exp randomized controlled trial/ or controlled clinical study/ or controlled study/ or randomi?ed controlled.ti,ab. or control group$1.ti,ab.) | 471104 |
| 47 | (((case adj control$) and random$) not randomi?ed controlled).ti,ab. | 24601 |
| 48 | Systematic review.ti,ab. not (trial or study).ti. | 426471 |
| 49 | (nonrandom$ not random$).ti,ab. | 21503 |
| 50 | "random field$".ti,ab. | 3241 |
| 51 | (random cluster adj3 sampl$).ti,ab. | 1805 |
| 52 | (review.ab. and review.pt.) not trial.ti. | 1334862 |
| 53 | "we searched".ab. and (review.ti. or review.pt.) | 60232 |
| 54 | "update review".ab. | 156 |
| 55 | (databases adj4 searched).ab. | 79573 |
| 56 | (rat or rats or mouse or mice or swine or porcine or murine or sheep or lambs or pigs or piglets or rabbit or rabbits or cat or cats or dog or dogs or cattle or bovine or monkey or monkeys or trout or marmoset$1).ti. and animal experiment/ | 1320922 |
| 57 | Animal experiment/ not (human experiment/ or human/) | 2795269 |
| 58 | or/45-57 | 5017112 |
| 59 | 44 not 58 | 6720677 |
| 60 | 5 and 24 and 59 | 7052 |

# Table S3: Search Strategy – Cochrane

Database(s): Cochrane Library <October 7, 2025>

Search Strategy:

| # | Searches | Results |
| --- | --- | --- |
| 1 | [mh "atrial fibrillation"] | 7554 |
| 2 | AF:ti,ab | 10270 |
| 3 | ((atrial or atria or atrium or auricular) NEAR/3 fibrillat*):ti,ab | 16074 |
| 4 | #1 or #2 or #3 | 18924 |
| 5 | [mh "Angiotensin Receptor Antagonists"] | 3014 |
| 6 | [mh "Angiotensin-Converting Enzyme Inhibitors"] | 5206 |
| 7 | (angiotensin converting enzyme inhibitors or ACE inhibitor or angiotensin receptor blockers or ARB):ti,ab | 7960 |
| 8 | (bepridil or captopril or cilazapril or enalapril or fosinopril or irbesartan or lisinopril or losartan or perindopril or quinapril or ramipril or telmisartan or valsartan or candesartan or irbesartan or telmisartan):ti,ab | 15991 |
| 9 | [mh "Mineralocorticoid Receptor Antagonists"] | 983 |
| 10 | (MRA or anti-aldosterone or aldosterone receptor antagonists or mineralocorticoid receptor antagonist or finerenone or spironolactone or eplerenone):ti,ab | 3964 |
| 11 | [mh "Sodium-Glucose Transporter 2 Inhibitors"] | 1228 |
| 12 | (sodium-glucose co-transporter or SGLT* or empagliflozin or dapagliflozin or canagliflozin or ertugliflozin):ti,ab | 6967 |
| 13 | (glucagon like peptide 1 receptor agonist or GLP* or exenatide or liraglutide or lixisenatide or dulaglutide or semaglutide):ti,ab | 10045 |
| 14 | [mh "Dipeptidyl-Peptidase IV Inhibitors"] | 828 |
| 15 | (DPP-4 inhibitors or DPP-4* or gliptins or sitagliptin or linagliptin or vildagliptin or saxagliptin or alogliptin or linagliptin):ti,ab | 4912 |
| 16 | [mh "Hydroxymethylglutaryl-CoA reductase Inhibitors"] | 5046 |
| 17 | (hydroxymethylglutaryl-co A reductase inhibitor or HMG-coA* or statin or Fluvastatin or pravastatin or lovastatin or simvastatin or atorvastatin or rosuvastatin):ti,ab | 17561 |
| 18 | [mh "Fatty Acids, Omega-3"] | 4217 |
| 19 | (Omega-3 Fatty Acid or Omega 3 Fatty Acid or n-3 Oil or n-3 Fatty Acids or Omega 3 Fatty Acids or n-3 PUFA or n3 Fatty Acid or n3 Polyunsaturated Fatty Acid or n-3 Oils or N-3 Fatty Acid or Fatty Acid, N-3 or n-3 Polyunsaturated Fatty Acid or n 3 Polyunsaturated Fatty Acid or Oil, n-3 or Omega 3 Fatty Acids or PUFA, n3):ti,ab | 7403 |
| 20 | #5 or #6 or #7 or #8 or #9 or #10 or #11 or #12 or #13 or #14 or #15 or #16 or #17 or #18 or #19 | 64845 |
| 21 | #4 and #20 | 1179 |

# Table S4: Indication of Trials by Class

|  | ACEI | ARB | ARNI | DPP-4 Inhibitor | GLP-1 RA | MRA | O3FA | SGLT2 Inhibitor | Statin | Total |
| --- | --- | --- | --- | --- | --- | --- | --- | --- | --- | --- |
| CKD |  |  |  |  | 1 | 1 |  | 2 | 2 | 6 |
| DM |  |  |  | 1 | 52 |  | 1 | 33 | 2 | 89 |
| DM w/ ETOD | 2 | 3 |  | 3 | 13 | 5 | 2 | 14 |  | 42 |
| HC |  |  |  |  |  |  |  |  | 4 | 4 |
| HFmEF |  |  |  |  |  |  | 1 | 1 | 1 | 3 |
| HFpEF |  | 2 | 3 |  | 1 | 2 |  | 6 | 1 | 15 |
| HFrEF | I | 2 | 6 |  |  | 3 |  | 8 | 3 | 24 |
| HL |  |  |  |  |  |  | I |  |  | 1 |
| HTN | 3 | 2 | 4 |  |  | 1 |  |  | 2 | 12 |
| MI |  |  |  |  |  |  | 1 | 1 |  | 2 |
| Obesity +/- DM |  |  |  |  | 18, 14 |  |  |  |  | 32 |
| Vasc. Disease* or Risk Factors |  | 1 |  |  |  | 1 | 2 |  | 7 | 11 |
| Open or Other |  |  |  |  |  |  | 2 |  | 3 | 5 |

*Abbreviations: CKD, Chronic Kidney Disease; DM, Diabetes Mellitus; DM w/ ETOD; Diabetes Mellitus with End Target Organ Damage; HC, Hypercholesterolaemia; HFmEF, Heart Failure with mixed Ejection Fraction; HFpEF, Heart Failure with preserved Ejection Fraction; HFrEF, Heart Failure with reduced Ejection Fraction; HL, Hyperlipidaemia; HTN, Hypertension; MI, Myocardial Infarction; Vasc., Vascular*

**Vascular disease only includes instances where “vascular disease” was explicitly stated as the indication. However, vascular disease as described in the main text comprises explicitly stated indications of “vascular disease”, “MI”, and “atherosclerotic cardiovascular disease.”*

# Table S5: Trial characteristics by pharmacotherapy class.

|  | | ACEi | ARB | ARNI | DPP-4 inhibitor | GLP-1 RA | MRA | O3FA | SGLT2 inhibitor | Statin | Total |
| --- | --- | --- | --- | --- | --- | --- | --- | --- | --- | --- | --- |
| RCTs (n) | | 6 | 10 | 13 | 4 | 99 | 13 | 10 | 69 | 25 | 249 |
| RCTs with AF as pre-specified endpoint (n) | | 1 | 3 | 1 | 0 | 0 | 5 | 3 | 0 | 1 | 15 |
| AF  Capture | ECG (12-lead) | 3(3) | 7(7) |  |  |  | 4 (0) | 5 (5) | 1 (1) | 7 (5) | 27 (21) |
|  | Adverse Events |  |  | 13 | 4 | 99 | 9 | 4 | 68 | 16 | 189 |
|  | Medical Records |  |  |  |  |  |  | 4 |  |  | 4 |
|  | Not Reported | 3 | 3 |  |  |  |  |  |  | 2 | 8 |
| Participants (n) | | 53 333 | 99 592 | 21 358 | 32 107 | 158 442 | 28 839 | 98 299 | 124 894 | 128 775 | 745 041 |
| Participant characteristics | Age | 64.3 | 65.9 | 66.8 | 64.0 | 59.6 | 66.6 | 64.8 | 63.3 | 63.3 | 63.5 |
|  | Male (%) | 49.1 | 62.9 | 64.0 | 66.2 | 58.2 | 62.6 | 60.7 | 61.8 | 70.2 | 61.9 |
|  | HF (%) | 89.4 | 77.7 | 85.1 | 85.2 | 78.8 | 90.2 | 67.8 | 75.8 | 63.7 | 72.7 |
|  | DM (%) | 41.3 | 64.1 | 35.2 | 100 | 86.8 | 70.2 | 51.6 | 87.5 | 21.3 | 56.8 |
|  | HTN (%) | 0.76 | 39.6 | 97.5 | 14.1 | 18.7 | 44.7 | 36.5 | 39.2 | 9.40 | 18.0 |

*Abbreviations: RCT, randomised controlled trial; ACEi/ARB, Angiotensin-Converting Enzyme Inhibitor/Angiotensin II Receptor Blocker; GLP-1 RA, Glucagon-Like Peptide-1 Receptor Agonist; SGLT2 inhibitor, Sodium-Glucose Cotransporter 2 Inhibitor; ARNI, Angiotensin Receptor-Neprilysin Inhibitor; DPP-4 inhibitor, Dipeptidyl Peptidase-4 Inhibitor; MRA, Mineralocorticoid Receptor Antagonist; O3FA, Omega-3 Fatty Acids; AF, Atrial Fibrillation; HTN, Hypertension; DM, Diabetes Mellitus; HF, Heart Failure*

# Table S6: Trial Characteristics (ACEI)

| **Study Name** | **NCT Number** | **Dose** | **Controls** | **Countries** | **Follow-up (mean years)** | **Total Participants** | **Indication** | **AF Capture** |
| --- | --- | --- | --- | --- | --- | --- | --- | --- |
| CAPPP | NA | captopril | usual care | Sweden, Finland | 6.1 | 10985 | HTN | AE: “atrial fibrillation” |
| SOLVD | NCT00000516 | enalapril | PLA | Canada | 2.9 | 374 | HFrEF | 12-lead ECG |
| STOP-H2 | NA | enalapril or lisinopril | felodipine/ isradipine or usual care | Sweden | 4 | 4418 | HTN | AE: “atrial fibrillation” |
| ALLHAT-2 | NCT00000542 | lisinopril | chlorthalidone | USA, Canada | 4.9 | 24309 | HTN | 12-lead ECG |
| HOPE | NCT00106886 | ramipril | PLA | 19 | 4.5* | 8335 | DM w/ ETOD | AE: “atrial fibrillation” |
| DIABHYCAR | NA | ramipril | PLA | 16 | 4* | 4912 | DM w/ ETOD | 12-lead ECG |

*Abbreviations: PLA, Placebo; DM, Diabetes Mellitus; DM w/ ETOD, Diabetes Mellitus w/ End Target Organ Damage; HFpEF, Heart Failure with preserved Ejection Fraction; HFrEF, Heart Failure with reduced Ejection Fraction; HTN, Hypertension; Vasc. Disease, Vascular Disease; AE, Adverse Event*

**Median value*

# Table S7: Baseline Cohort Characteristics (ACEI)

| **Study Name** | **Participants (n)** | | | **Age** | **Male (%)** | **Comorbidity (%)** | | | | | |
| --- | --- | --- | --- | --- | --- | --- | --- | --- | --- | --- | --- |
|  | **Total** | **Intervention** | **Control** |  |  | **HTN** | **DM** | **HF** | **PVD** | **MI** | **IHD** |
| CAPPP | 10985 | 5492 | 5493 | 52.5 | 53.4 | 100 | 5.20 | 0.26 | NA | 47.5 | 72.5 |
| SOLVD | 374 | 186 | 188 | 57 | 90.4 | 19.0 | 20.0 | 100 | NA | NA | 93.7 |
| STOP-H2 | 4418 | 2205 | 2213 | 76 | 66.8 | 100 | 10.9 | 1.9 | NA | 3.1 | 8.0 |
| ALLHAT-2 | 24309 | 9054 | 15255 | 67 | 47 | 100 | 35.5 | 0 | NA | NA | 25.7 |
| HOPE | 8335 | 4291 | 4044 | 65.7 | 26.1 | 45.7 | 100 | 0 | NA | 53.26 | NA |
| DIABHYCAR | 4912 | 2443 | 2469 | 65.1 | 69.9 | 55.7 | 100 | 0 | 10.2 | 6.0 | NA |

*Abbreviations: BMI, Body Mass Index; SBP, Systolic Blood Pressure; HTN, Hypertension; DM, Diabetes Mellitus; HF, Heart Failure; PVD, Peripheral Vascular Disease; MI, Myocardial Infarction; IHD, Ischaemic Heart Disease*

# Table S8: Trial Characteristics (ARB)

| **Study Name** | **NCT Number** | **Dose** | **Controls** | **Countries** | **Follow-up (mean years)** | **Total Participants** | **Indication** | **AF Capture** |
| --- | --- | --- | --- | --- | --- | --- | --- | --- |
| CHARM | NCT00634400 | candesartan | PLA | 26 | 3.1* | 5409 | HFrEF | 12-lead ECG |
| I-PRESERVE | NCT00095238 | irbesartan | PLA | 25 | 4.1 | 4128 | HFpEF | AE: “atrial fibrillation” |
| LIFE | NCT00338260 | losartan | atenolol | Denmark, Finland, Iceland, Norway, Sweden, UK, and USA | 4.8 | 8480 | HTN | 12-lead ECG |
| SUPPORT | NCT00417222 | olmesartan | no treatment | Japan | 4.4* | 1147 | HFpEF | AE: “atrial fibrillation” |
| TRANSCEND | NCT00153101 | telmisartan | PLA | 40 | 4.7* | 5926 | DM w/ ETOD | 12-lead ECG |
| ProFESS | NCTC00153062 | telmisartan | PLA | 35 | 2.4* | 20332 | Vasc. disease | AE: “atrial fibrillation” |
| ONTARGET | [NCT00153101](https://clinicaltrials.gov/show/NCT00153101) | telmisartan | ramipril | 40 | 4.7* | 25620 | CVD/DM | 12-lead ECG |
| VALUE | NA | valsartan | amlodipine | 31 | 4.2 | 14849 | HTN | 12-lead ECG |
| ValHeFt | NA | valsartan | PLA | 16 | 1.9 | 4395 | HFrEF | 12-lead ECG |
| NAVIGATOR | NCT00097786 | valsartan | PLA | 40 | 6.5* | 9306 | DM | 12-lead ECG |

*Abbreviations: PLA, Placebo; CVD, Cardiovascular Disease; DM, Diabetes Mellitus; DM w/ ETOD, Diabetes Mellitus w/ End Target Organ Damage; HFpEF, Heart Failure with preserved Ejection Fraction; HFrEF, Heart Failure with reduced Ejection Fraction; HTN, Hypertension; Vasc. Disease, Vascular Disease; AE, Adverse Event*

**Median value*

Table S9: Baseline Cohort Characteristics (ARB)

| **Study Name** | **Participants (n)** | | | **Age** | **Male (%)** | **Comorbidity (%)** | | | | | |
| --- | --- | --- | --- | --- | --- | --- | --- | --- | --- | --- | --- |
|  | **Total** | **Intervention** | **Control** |  |  | **HTN** | **DM** | **HF** | **PVD** | **MI** | **IHD** |
| CHARM | 5409 | 2700 | 2709 | 66 | 68.4 | 54.8 | 28.99 | 100 | NA | 28.96 | 61.60 |
| I-PRESERVE | 4128 | 2061 | 2067 | 72 | 40 | 88 | 27 | 100 | NA | 23.5 | 25 |
| LIFE | 8480 | 4298 | 4182 | 71 | 51 | 100 | 13.6 | 0 | 4.4 | NA | 4.1 |
| SUPPORT | 1147 | 578 | 569 | 66 | 75 | 100 | 50.2 | 100 | NA | NA | 47.5 |
| TRANSCEND | 5926 | 2954 | 2972 | 66.9 | 57 | 35.7 | 100 | 0 | 11.3 | 46.3 | 74.6 |
| ProFESS | 20332 | 10146 | 10186 | 66.1 | 64 | NA | NA | NA | NA | NA | NA |
| ONTARGET | 25620 | 8218 | 8296 | 66.4 | 73.8 | 68.7 | 37.2 | 0 | 13.5 | 48.9 | 74.6 |
| VALUE | 14849 | 7450 | 7399 | 67.2 | 57.6 | 100 | NA | NA | 13.9 | NA | 45.8 |
| ValHeFt | 4395 | 2205 | 2190 | 67 | 79 | NA | 25.2 | 100 | NA | NA | 57.4 |
| NAVIGATOR | 9306 | 4631 | 4675 | 54 | 51 | 79.6 | 100 | NA | NA | 11.7 | NA |

*Abbreviations: BMI, Body Mass Index; SBP, Systolic Blood Pressure; HTN, Hypertension; DM, Diabetes Mellitus; HF, Heart Failure; PVD, Peripheral Vascular Disease; MI, Myocardial Infarction; IHD, Ischaemic Heart Disease*

# Table S10: Trial Characteristics (ARNI)

| **Study Name** | **NCT Number** | **Dose** | **Controls** | **Countries** | **Follow-up (median years)** | **Total Participants** | **Indication** | **AF Capture** |
| --- | --- | --- | --- | --- | --- | --- | --- | --- |
| PARALLAX | NCT03066804 | sacubitril/  valsartan | Background individualised medication | 32 | 0.5* | 2566 | HFpEF | AE: “atrial fibrillation” |
| OUTSTEP-HF | NCT02900378 | sacubitril/  valsartan | enalapril | 19 | 0.2* | 621 | HFrEF | AE: “atrial fibrillation” |
| EVALUATE-HF | NCT02874794 | sacubitril/  valsartan | enalapril | USA | 0.2* | 464 | HFrEF | AE: “atrial fibrillation” |
| ACTIVITY-HF | NCT02768298 | sacubitril/  valsartan | enalapril | Germany | 0.3* | 201 | HFrEF | AE: “atrial fibrillation” |
| PARADIGM-HF | NCT01035255 | sacubitril/  valsartan | enalapril | 47 | 1.1 | 8399 | HFrEF | AE: “atrial fibrillation” |
| PARALLEL-HF | NCT02468232 | sacubitril/  valsartan | enalapril | Japan | 1.6 | 225 | HFrEF | AE: “atrial fibrillation” |
| Efficacy, Safety of LCZ696 vs. Olmesartan in Asian Patients w/ Essential HTN | NCT01785472 | sacubitril/  valsartan | olmesartan | China, HK, SK, Philippines, Singapore, Taiwan, Thailand | 0.2* | 1438 | HTN | AE: “atrial fibrillation” |
| PARAMETER | NCT01692301 | sacubitril/  valsartan | olmesartan | 12 | 1* | 454 | HTN | AE: “atrial fibrillation” |
| Efficacy, Safety of LCZ696 vs. Olmesartan in Elderly Asian Patients w/ HTN | NCT01615198 | sacubitril/  valsartan | olmesartan | China, HK, Japan, SK, Philippines, Taiwan, Thailand | 0.3* | 588 | HTN | AE: “atrial fibrillation” |
| Efficacy of LCZ696 versus olmesartan in Japanese patients with essential HTN | NCT01599104 | sacubitril/  valsartan | olmesartan | Japan | 0.2* | 1161 | HTN | AE: “atrial fibrillation” |
| PARAGON-HF | NCT01920711 | sacubitril/  valsartan | valsartan | 43 | 2.9 | 4822 | HFpEF | AE: “atrial fibrillation” |
| PARAMOUNT | NCT00887588 | sacubitril/  valsartan | valsartan | 13 | 0.3* | 301 | HFpEF | AE: “atrial fibrillation” |
| PRIME | NCT02687932 | sacubitril/  valsartan | valsartan | South Korea | 1 | 118 | HFrEF | AE: “atrial fibrillation” |

*Abbreviations: PLA, Placebo; HFpEF, Heart Failure with preserved Ejection Fraction; HFrEF, Heart Failure with reduced Ejection Fraction; HTN, Hypertension; AE, Adverse Event*

**Derived value*

# Table S11: Baseline Cohort Characteristics (ARNI)

| **Study Name** | **Participants (n)** | | | **Age** | **Male (%)** | **Comorbidity (%)** | | | | | |
| --- | --- | --- | --- | --- | --- | --- | --- | --- | --- | --- | --- |
|  |  |  |  |  |  | **HTN** | **DM** | **HF** | **PVD** | **MI** | **IHD** |
|  | **Total** | **Intervention** | **Control** |  |  |  |  |  |  |  |  |
| PARALLAX | 2566 | 1281 | 1285 | 72.7 | 49.3 | 97.2 | 45.0 | 100 | NA | 0 | NA |
| OUTSTEP-HF | 621 | 310 | 311 | 66.8 | 78.6 | 67.2 | 34.4 | 100 | 6.77 | 45.56 | 43.13 |
| EVALUATE-HF | 464 | 231 | 233 | 67.3 | 76.5 | 100 | NA | 100 | NA | NA | 61 |
| ACTIVITY-HF | 201 | 103 | 98 | 66.9 | 81.1 | NA | NA | 100 | NA | 55.7 | NA |
| PARADIGM-HF | 8399 | 4187 | 4212 | 63.8 | 78.2 | 70.4 | 34.7 | 100 | NA | 43.2 | NA |
| PARALLEL-HF | 225 | 112 | 113 | 67.8 | 86.1 | 68.0 | 46.6 | 100 | NA | 43.55 | 47.5 |
| Efficacy, Safety of LCZ696 vs. Olmesartan in Asian Patients w/ Essential HTN | 1438 | 952 | 486 | 57.7 | 52.6 | 100 | 16.2 | NA | NA | NA | NA |
| PARAMETER | 454 | 229 | 225 | 67.7 | 52.2 | 100 | 28.7 | 0 | NA | NA | NA |
| Efficacy, Safety of LCZ696 vs. Olmesartan in Elderly Asian Patients w/ HTN | 588 | 296 | 292 | 70.7 | 50 | 100 | NA | NA | NA | NA | NA |
| Efficacy of LCZ696 versus olmesartan in Japanese patients with essential HTN | 1161 | 772 | 389 | 58.7 | 70.5 | 100 | 9.2 | NA | NA | 0 | NA |
| PARAGON-HF | 4822 | 2419 | 2403 | 72.7 | 48.3 | 95.5 | 43.0 | 100 | NA | 22.6 | 35.8 |
| PARAMOUNT | 301 | 149 | 152 | 71.1 | 43.5 | 93.5 | 38 | 100 | NA | 20.59 | NA |
| PRIME | 118 | 60 | 58 | 62.6 | 61.15 | 41.5 | 30.0 | 100 | NA | 19.49 | NA |

*Abbreviations: BMI, Body Mass Index; SBP, Systolic Blood Pressure; HTN, Hypertension; DM, Diabetes Mellitus; HF, Heart Failure; PVD, Peripheral Vascular Disease; MI, Myocardial Infarction; IHD, Ischaemic Heart Disease*

# Table S12: Trial Characteristics (DPP-4 Inhibitor)

| **Study Name** | **NCT Number** | **Dose** | **Controls** | **Countries** | **Follow-up (median years)** | **Total Participants** | **Indication** | **AF Capture** |
| --- | --- | --- | --- | --- | --- | --- | --- | --- |
| EXAMINE | NCT0096870 | alogliptin | PLA | 49 | 1.5 | 5380 | DM w/ ETOD | AE: “atrial fibrillation” |
| CAROLINA | NCT0124342 | linagliptin | glimepiride | 43 | 6.3 | 6033 | DM | AE: “atrial fibrillation” |
| NCT01703208 | NCT0170320 | omarigliptin | PLA | USA | 1.8 | 4202 | DM w/ ETOD | AE: “atrial fibrillation” |
| SAVOR-TIMI 53 | NCT0110788 | saxagliptin | PLA | 26 | 2.9 | 16492 | DM w/ ETOD | AE: “atrial fibrillation” |

*Abbreviations: PLA, Placebo; DM, Diabetes Mellitus; DM w/ ETOD, Diabetes Mellitus w/ End Target Organ Damage; AE, Adverse Event*

# Table S13: Baseline Cohort Characteristics (DPP-4 Inhibitor)

| **Study Name** | **Participants (n)** | | | **Age** | **Male (%)** | **Comorbidity (%)** | | | | | |
| --- | --- | --- | --- | --- | --- | --- | --- | --- | --- | --- | --- |
|  | **Total** | **Intervention** | **Control** |  |  | **HTN** | **DM** | **HF** | **PVD** | **MI** | **IHD** |
| EXAMINE | 5380 | 2701 | 2679 | 61 | 67.8 | 83.1 | 100 | 27.9 | 9.6 | 87.9 | NA |
| CAROLINA | 6033 | 3023 | 3010 | 64 | 60.1 | 89.9 | 100 | 4.5 | 6.8 | NA | 31.6 |
| NCT01703208 | 4202 | 2100 | 2102 | 63.6 | 70 | 95.3 | 100 | 15.2 | NA | NA | NA |
| SAVOR-TIMI 53 | 16492 | 8280 | 8212 | 65 | 66.9 | 81.6 | 100 | 12.8 | NA | 37.8 | NA |

*Abbreviations: BMI, Body Mass Index; SBP, Systolic Blood Pressure; HTN, Hypertension; DM, Diabetes Mellitus; HF, Heart Failure; PVD, Peripheral Vascular Disease; MI, Myocardial Infarction; IHD, Ischaemic Heart Disease*

# Table S14: Trial Characteristics (GLP-1 RA)

| **Study Name** | **NCT Number** | **Dose** | **Controls** | **Countries** | **Follow-up (mean years)** | **Total Participants** | **Indication** | **AF Capture** |
| --- | --- | --- | --- | --- | --- | --- | --- | --- |
| HARMONY 1 | NCT00849056 | albiglutide | PLA + piaglitazone | 4 | 1 | 310 | DM | AE: “atrial fibrillation” |
| HARMONY 2 | NCT00849017 | albiglutide | PLA | USA, Mexico | 1 | 309 | DM | AE: “atrial fibrillation” |
| HARMONY 4 | NCT00838916 | albiglutide | insulin + metformin +/- sulfonylurea | USA, UK, Russia, SA | 1 | 745 | DM | AE: “atrial fibrillation” |
| HARMONY 5 | NCT00839527 | albiglutide | pioglitazone or PLA + metformin + glimepiride | 9 | 1 | 685 | DM | AE: “atrial fibrillation” |
| HARMONY 6 | NCT00976391 | albiglutide | insulin | 14 | 0.5 | 586 | DM | AE: “atrial fibrillation” |
| HARMONY 8 | NCT01098539 | albiglutide | sitagliptin | 15 | 0.5 | 500 | DM with ETOD | AE: “atrial fibrillation” |
| HARMONY outcomes | NCT02465515 | albiglutide | PLA | 28 | 1.6 | 8892 | DM with ETOD | AE: “atrial fibrillation” |
| AWARD 1 | NCT01064687 | dulaglutide | PLA | US, Argentina, Mexico, Puerto Rico | 1 | 976 | DM | AE: “atrial fibrillation” |
| AWARD 2 | NCT01075282 | dulaglutide | insulin | 20 | 1.6 | 810 | DM | AE: “atrial fibrillation” |
| AWARD 3 | NCT01126580 | dulaglutide | metformin |  | 1 | 807 | DM | AE: “atrial fibrillation” |
| AWARD 4 | NCT01191268 | dulaglutide | insulin | 15 | 1 | 884 | DM | AE: “atrial fibrillation” |
| AWARD 5 | NCT00734474 | dulaglutide | sitagliptin or PLA | 13 | 2 | 1098 | DM | AE: “atrial fibrillation” |
| AWARD 7 | NCT01621178 | dulaglutide | insulin | 9 | 1 | 482 | DM with ETOD | AE: “atrial fibrillation” |
| AWARD 9 | NCT02152371 | dulaglutide | PLA | 8 | 0.5 | 300 | DM | AE: “atrial fibrillation” |
| AWARD 10 | NCT02597049 | dulaglutide | PLA | 8 | 0.5 | 423 | DM | AE: “atrial fibrillation” |
| AWARD-CHN2 | NCT01648582 | dulaglutide | insulin | China, SK, Mexico, Russia | 0.6 | 774 | DM | AE: “atrial fibrillation” |
| NCT00791479 | NCT00791479 | dulaglutide | PLA | 9 | NA | 167 | DM | AE: “atrial fibrillation” |
| REWIND | NCT01394952 | dulaglutide | PLA | 24 | 5.4* | 9892 | DM | AE: “atrial fibrillation” |
| AMPLITUDE-O | NCT03496298 | efpeglenatide | PLA | 28 | 1.8 | 4073 | DM | AE: “atrial fibrillation” |
| Derosa | NA | exenatide | glimepiride | Italy | 1 | 110 | DM | AE: “atrial fibrillation” |
| DURATION 8 | NCT02229396 | exenatide | dapagliflozin | 6 | 2 | 457 | DM | AE: “atrial fibrillation” |
| EUREXA | NCT00359762 | exenatide | glimepiride | 14 | 4 | 1029 | DM | AE: “atrial fibrillation” |
| EXSCEL | NCT01144338 | exenatide | PLA | 35 | 3.2 | 14716 | DM | AE: “atrial fibrillation” |
| NCT00082407 | NCT00082407 | exenatide | insulin | 13 | 1 | 501 | DM | AE: “atrial fibrillation” |
| NCT00701935 | NCT00701935 | exenatide | PLA | USA, Canada | 0.5 | 80 | DM | AE: “atrial fibrillation” |
| NCT00855439 | NCT00855439 | exenatide | insulin | USA | 1.5 | 46 | DM with ETOD | AE: “atrial fibrillation” |
| NCT00935532 | NCT00935532 | exenatide | insulin | Japan | 0.5 | 427 | DM | AE: “atrial fibrillation” |
| A Study of LY2189265 in Japanese Participants With Type 2 DM | NCT01558271 | liraglutide | PLA | Japan | 1 | 50 | DM | AE: “atrial fibrillation” |
| Effect of Adding Liraglutide vs PLA to a High-Dose lnsulin Regimen in Patients With Type 2 DM | NCT01505673 | liraglutide | PLA | USA | 0.5 | 1091 | DM | AE: “atrial fibrillation” |
| ELEGANT | NCT01392898 | liraglutide | insulin | Netherlands | 0.5 | 745 | DM | AE: “atrial fibrillation” |
| LEAD-2 | NCT00318461 | liraglutide | PLA/glimepiride + metformin | 21 | 0.5 | 9340 | DM | AE: “atrial fibrillation” |
| LEAD-3 | NCT00294723 | liraglutide | glimepiride | USA, Mexico | 2 | 124 | DM | AE: “atrial fibrillation” |
| LEADER | NCT01179048 | liraglutide | PLA | 32 | 3.8* | 653 | DM | AE: “atrial fibrillation” |
| MDI-Liraglutide | NCT02113332 | liraglutide | PLA | Sweden | 0.5 | 71 | DM | AE: “atrial fibrillation” |
| NCT01296412 | NCT01296412 | liraglutide | sitagliptin | 21 | 1 | 492 | DM | AE: “atrial fibrillation” |
| NCT02008682 | NCT02008682 | liraglutide | sitagliptin | China | 0.5 | 368 | DM | AE: “atrial fibrillation” |
| NCT02545738 | NCT02545738 | liraglutide | PLA/liraglutide | Denmark | 0.5 | 64 | DM | AE: “atrial fibrillation” |
| SCALE Insulin | NCT02963922 | liraglutide | PLA | 7 | 1.1 | 396 | DM | AE: “atrial fibrillation” |
| SCALE Obesity and PreDM | NCT01272219 | liraglutide | PLA | 27 | 1.1 | 2254 | Pre-DM | AE: “atrial fibrillation” |
| NCT00422058 | NCT00422058 | liraglutide | PLA | 8 | 1.6 | 283 | Obesity | AE: “atrial fibrillation” |
| ELIXA | NCT01147250 | lixisenatide | PLA | 49 | 2.1 | 6063 | DM | AE: “atrial fibrillation” |
| GetGoal-Duo-2 | NCT01768559 | lixisenatide | insulin | 18 | 0.5 | 890 | DM | AE: “atrial fibrillation” |
| GETGOAL-F1 | NCT00763451 | lixisenatide | PLA | 15 | 0.5 | 484 | DM | AE: “atrial fibrillation” |
| GETGOAL-L | NCT00715624 | lixisenatide | PLA | 15 | 0.5 | 495 | DM | AE: “atrial fibrillation” |
| GETGOAL-M | NCT00712673 | lixisenatide | PLA | 16 | 0.5 | 680 | DM | AE: “atrial fibrillation” |
| NCT02207374 | NCT02207374 | semaglutide | usual care | Japan | 1.1 | 601 | DM | AE: “atrial fibrillation” |
| PIONEER 1 | NCT02906930 | semaglutide | PLA | 9 | 0.6 | 703 | DM | AE: “atrial fibrillation” |
| PIONEER 2 | NCT02863328 | semaglutide | empagliflozin | 12 | 1.0 | 821 | DM | AE: “atrial fibrillation” |
| PIONEER 3 | NCT02607865 | semaglutide | sitagliptin | 14 | 1.5 | 1863 | DM | AE: “atrial fibrillation” |
| PIONEER 4 | NCT02863419 | semaglutide | PLA | 12 | 1.0 | 711 | DM | AE: “atrial fibrillation” |
| PIONEER 5 | NCT02827708 | semaglutide | PLA | 8 | 1.0 | 324 | DM with ETOD | AE: “atrial fibrillation” |
| PIONEER 6 | NCT02692716 | semaglutide | PLA | 21 | 1.3 | 3183 | DM with ETOD | AE: “atrial fibrillation” |
| PIONEER 7 | NCT02849080 | semaglutide | sitagliptin | 10 | 1.1 | 504 | DM | AE: “atrial fibrillation” |
| PIONEER 8 | NCT03021187 | semaglutide | PLA | 9 | 1 | 731 | DM | AE: “atrial fibrillation” |
| PIONEER 9 | NCT03018028 | semaglutide | PLA | Japan | 1.1 | 243 | DM | AE: “atrial fibrillation” |
| PIONEER 10 | NCT03015220 | semaglutide | dulaglutide | Japan | 1.1 | 458 | DM | AE: “atrial fibrillation” |
| SELECT | NCT03574597 | semaglutide | placebo | 41 | 2.0 | 17604 | Obesity | AE: “atrial fibrillation” |
| STEP 1 | NCT03548935 | semaglutide | PLA | 16 | 1.3 | 1961 | Obesity | AE: “atrial fibrillation” |
| STEP 2 | NCT03552757 | semaglutide | PLA | 12 | 1.3 | 1210 | Obesity | AE: “atrial fibrillation” |
| STEP 4 | NCT03548987 | semaglutide | PLA | 10 | 1.3 | 803 | Obesity | AE: “atrial fibrillation” |
| STEP 6 | NCT03811574 | semaglutide | PLA | Japan, South Korea | 1.3 | 401 | Obesity | AE: “atrial fibrillation” |
| STEP 10 | NCT05040971 | semaglutide | PLA | 5 | 1.3 | 207 | Obesity | AE: “atrial fibrillation” |
| STEP 11 | NCT04998136 | semaglutide | PLA | South Korea, Thailand | 1.1 | 150 | Obesity | AE: “atrial fibrillation” |
| STEP-HFpEF | NCT04788511 | semaglutide | PLA | 14 | 1.1 | 616 | Obesity | AE: “atrial fibrillation” |
| STEP-HFpEF DM | NCT04916470 | semaglutide | PLA | 16 | 1.0 | 529 | DM | AE: “atrial fibrillation” |
| STEP UP | NCT05646706 | semaglutide | PLA | 11 | 1.6 | 1407 | Obesity | AE: “atrial fibrillation” |
| STEP UP T2D | NCT05649137 | semaglutide | PLA | 8 | 1.6 | 512 | Obesity | AE: “atrial fibrillation” |
| SUSTAIN 1 | NCT02054897 | semaglutide | PLA | 8 | 0.7 | 388 | DM | AE: “atrial fibrillation” |
| SUSTAIN 2 | NCT01930188 | semaglutide | sitagliptin | 18 | 1.1 | 1231 | DM | AE: “atrial fibrillation” |
| SUSTAIN 3 | NCT01885208 | semaglutide | exenatide | 12 | 1.2 | 809 | DM | AE: “atrial fibrillation” |
| SUSTAIN 4 | NCT02128932 | semaglutide | insulin | 14 | 0.6 | 1082 | DM | AE: “atrial fibrillation” |
| SUSTAIN 5 | NCT02305381 | semaglutide | PLA | 5 | 0.7 | 396 | DM | AE: “atrial fibrillation” |
| SUSTAIN 6 | NCT01720446 | semaglutide | PLA | 20 | 2.0 | 3297 | DM | AE: “atrial fibrillation” |
| SUSTAIN 7 | NCT02648204 | semaglutide | dulaglutide | 16 | 0.9 | 1201 | DM | AE: “atrial fibrillation” |
| SUSTAIN 8 | NCT03136484 | semaglutide | canagliflozin | 11 | 1.1 | 788 | DM | AE: “atrial fibrillation” |
| SUSTAIN 9 | NCT03086330 | semaglutide | PLA | 6 | 0.7 | 302 | DM | AE: “atrial fibrillation” |
| SUSTAIN 10 | NCT03191396 | semaglutide | liraglutide | 11 | 0.7 | 577 | DM | AE: “atrial fibrillation” |
| SUSTAIN 11 | NCT03689374 | semaglutide | PLA | 21 | 1.0 | 1748 | DM | AE: “atrial fibrillation” |
| SUSTAIN-CHINA | NCT03061214 | semaglutide | sitagliptin | 7 | 0.6 | 868 | DM | AE: “atrial fibrillation” |
| SURPASS-AP-Combo | NCT04093752 | tirzepatide | insulin | 4 | 0.8 | 907 | DM | AE: “atrial fibrillation” |
| SURPASS 2 | NCT03987919 | tirzepatide | semaglutide | 9 | 5.0 | 1878 | DM | AE: “atrial fibrillation” |
| SURPASS 3 | NCT03882970; NCT03730662 | tirzepatide | insulin | 13 | 1.0 | 1444 | DM | AE: “atrial fibrillation” |
| SURPASS 4 | NCT03882970; NCT03730662 | tirzepatide | insulin | 14 | 1.0 | 2002 | DM | AE: “atrial fibrillation” |
| SURMOUNT-1 | NCT04184622 | tirzepatide | PLA | 9 | 3.7 | 2539 | Obesity | AE: “atrial fibrillation” |
| SURMOUNT-2 | NCT04657003 | tirzepatide | PLA | 7 | 1.5 | 938 | Obesity | AE: “atrial fibrillation” |
| SURMOUNT 5 | NCT05822830 | tirzepatide | semaglutide | United States | 1.4 | 751 | Obesity | AE: “atrial fibrillation” |
| SURMOUNT-J | NCT04844918 | tirzepatide | PLA | Japan | 1.5 | 267 | Obesity | AE: “atrial fibrillation” |
| SEMA-AP | NCT05205928 | semaglutide | PLA | Canada | 0.3 | 28 | DM | AE: “atrial fibrillation” |
| NCT04889183 | NCT04889183 | semaglutide | PLA | Netherlands, Germany, Spain, Canada | 0.5 | 101 | CKD | AE: “atrial fibrillation” |
| ATTAIN-1 | NCT05869903 | orforglipron | PLA | 9 | 1.4 | 3127 | Obesity | AE: “atrial fibrillation” |
| OASIS-4 | NCT05564117 | semaglutide | PLA | Canada, Germany, Poland, United States | 1.4 | 205 | Obesity | AE: “atrial fibrillation” |
| MariTide Phase 2 | NCT05669599 | maridebart cafraglutide | PLA | United States, Australia | 1.0 | 592 | Obesity | AE: “atrial fibrillation” |
| ACHIEVE-1 | NCT05971940 | orforglipron | PLA | United States | 0.8 | 559 | DM | AE: “atrial fibrillation” |
| REDEFINE 1 | NCT05567796 | Cagrilinitide/  semaglutide | PLA | 22 | 1.1 | 3417 | Obesity | AE: “atrial fibrillation” |
| REDEFINE 2 | NCT05394519 | Cagrilinitide/  semaglutide | PLA | 12 | 1.3 | 1206 | Obesity and T2DM | AE: “atrial fibrillation” |
| SOUL | NCT03914326 | semaglutide | PLA | 33 | 4.1 | 9650 | DM with ETOD | AE: “atrial fibrillation” |
| SUMMIT | NCT04847557 | tirzepatide | PLA | 9 | 2.0 | 731 | HFpEF | AE: “atrial fibrillation” |
| EECOH-2 | NCT05680129 | ecnoglutide | dulaglutide | China | 1.0 | 623 | DM | AE: “atrial fibrillation” |
| NCT04867785 | NCT04867785 | retatrutide | PLA | United States | 0.8 | 281 | DM | AE: “atrial fibrillation” |
| STRIDE | NCT04560998 | semaglutide | PLA | 20 | 1.0 | 792 | DM and ETOD | AE: “atrial fibrillation” |
| FLOW | NCT03819153 | semaglutide | PLA | 28 | 3.4 | 3553 | DM and ETOD | AE: “atrial fibrillation” |

*Abbreviations: PLA, Placebo; DM, Diabetes Mellitus; DM w/ ETOD, Diabetes Mellitus w/ End Target Organ Damage; AE, Adverse Event*

**Median value*

# Table S15: Baseline Cohort Characteristics (GLP-1 RA)

| **Study Name** | **Participants (n)** | | | **Age** | **Male (%)** | **SBP (mmHg)** | **BMI** | **Comorbidity (%)** | | | | | |
| --- | --- | --- | --- | --- | --- | --- | --- | --- | --- | --- | --- | --- | --- |
|  | **Total** | **Intervention** | **Control** |  |  |  |  | **HTN** | **DM** | **HF** | **PVD** | **MI** | **IHD** |
| HARMONY 1 | 310 | 155 | 155 | 55 | 59.8 | 127.1 | 34.1 | NA | 100 | NA | NA | NA | NA |
| HARMONY 2 | 309 | 204 | 105 | 52.9 | 55.1 | NA | 33.5 | NA | 100 | NA | NA | 3 | NA |
| HARMONY 4 | 745 | 504 | 241 | 55.5 | 56.1 | 130.9 | 33.1 | NA | 100 | NA | NA | 5.0 | NA |
| HARMONY 5 | 685 | 281 | 404 | 55.2 | 53.2 | NA | 32.2 | NA | 100 | NA | NA | 4.2 | NA |
| HARMONY 6 | 586 | 292 | 294 | 55.5 | 47 | NA | NA | NA | 100 | NA | NA | 8.6 | NA |
| HARMONY 8 | 500 | 250 | 250 | 63.3 | 53.7 | 132.9 | 30.4 | NA | 100 | NA | NA | 8.7 | NA |
| HARMONY outcomes | 8892 | 4717 | 4175 | 64.1 | 70 | 134.8 | 32.3 | 86.5 | 100 | 20 | 24.5 | 47 | 70.5 |
| AWARD 1 | 976 | 835 | 141 | 55.5 | 58 | 127 | 33 | NA | 100 | NA | NA | NA | NA |
| AWARD 2 | 810 | 540 | 270 | 57 | 51.4 | 131 | 31.7 | NA | 100 | NA | NA | NA | NA |
| AWARD 3 | 807 | 539 | 268 | 55.7 | 43.67 | 129.7 | 33.3 | NA | 100 | NA | NA | NA | NA |
| AWARD 4 | 884 | 588 | 296 | 59.4 | 53.33 | 133.5 | 32.5 | NA | 100 | NA | NA | NA | NA |
| AWARD 5 | 1098 | 606 | 492 | 54 | 47.4 | NA | 31 | NA | 100 | NA | NA | NA | NA |
| AWARD 7 | 482 | 292 | 190 | 64.6 | 52.3 | 137 | 32.5 | NA | 100 | NA | NA | NA | NA |
| AWARD 9 | 300 | 150 | 150 | 60.4 | 57.7 | 136.3 | 32.7 | NA | 100 | NA | NA | NA | NA |
| AWARD 10 | 423 | 283 | 140 | 57.3 | 50 | 130.2 | 32.7 | NA | 100 | NA | NA | NA | NA |
| AWARD-CHN2 | 774 | 516 | 258 | 55 | 55.2 | 130.5 | 26.8 | NA | 100 | NA | NA | NA | NA |
| NCT00791479 | 167 | 135 | 32 | NA | NA | NA | NA | NA | 100 | NA | NA | NA | NA |
| REWIND | 9892 | 4943 | 4949 | 66.2 | 53.7 | 137.2 | 32.3 | 93.2 | 100 | 8.6 | NA | NA | NA |
| AMPLITUDE-O | 4073 | 2718 | 1355 | 64.5 | 67 | 134.9 | 32.7 | 91.3 | 100 | 18.1 | NA | NA | NA |
| Derosa | 110 | 55 | 55 | 56 | 47.37 | NA | 28.4 | NA | 100 | NA | NA | NA | NA |
| DURATION 8 | 457 | 227 | 230 | 54.2 | 47.89 | 129.7 | 32.4 | NA | 100 | NA | NA | NA | NA |
| EUREXA | 1029 | 515 | 514 | 56 | 54 | 133.1 | 32.4 | NA | 100 | NA | NA | NA | NA |
| EXSCEL | 14716 | 7344 | 7372 | 62 | 62 | NA | 31.8 | NA | 100 | 16.1 | 19.0 | NA | 52.8 |
| NCT00082407 | 501 | 253 | 248 | 58.5 | 49 | 137 | 30.4 | NA | 100 | NA | NA | NA | NA |
| NCT00701935 | 80 | 43 | 37 | NA | NA | NA | NA | NA | 100 | NA | NA | NA | NA |
| NCT00855439 | 46 | 22 | 24 | 51.5 | 56.52 | NA | 36 | NA | 100 | NA | NA | NA | NA |
| NCT00935532 | 427 | 215 | 212 | 57 | 67.9 | NA | 26.15 | NA | 100 | NA | NA | NA | NA |
| ELEGANT | 50 | 26 | 24 | 58 | 62 | 139 | 32.6 | NA | 100 | NA | NA | NA | NA |
| LEAD-2 | 1091 | 727 | 364 | 57 | 58.4 | 132.2 | 30.5 | NA | 100 | NA | NA | NA | NA |
| LEAD-3 | 745 | 497 | 248 | 53.0 | 50 | 128.7 | 33.1 | NA | 100 | NA | NA | NA | NA |
| LEADER | 9340 | 4668 | 4672 | 64.3 | 64.2 | 135.9 | 32.5 | NA | 100 | 14 | NA | 30.7 | 35.2 |
| MDI-Liraglutide | 124 | 64 | 60 | 63.6 | 64.5 | 135.8 | 33.6 | NA | 100 | NA | NA | 12.9 | NA |
| NCT01296412 | 653 | 326 | 327 | 57.3 | 55 | 130.9 | 32.6 | NA | 100 | NA | NA | NA | NA |
| NCT01505673 | 71 | 35 | 36 | 54.2 | 37 | 136 | 41.2 | NA | 100 | NA | NA | NA | NA |
| NCT01558271 | 492 | 422 | 70 | 57.4 | 81 | NA | 25.5 | NA | 100 | NA | NA | NA | NA |
| NCT02008682 | 368 | 184 | 184 | 51.6 | 59.65 | NA | 27.3 | NA | 100 | NA | NA | NA | NA |
| NCT02545738 | 64 | 32 | 32 | 66.5 | 82.5 | 138 | 31.5 | NA | 100 | 0 | NA | NA | NA |
| SCALE Insulin | 396 | 198 | 198 | 56.7 | 47.75 | 130.5 | 35.6 | NA | 100 | NA | NA | NA | NA |
| SCALE Obesity and PreDM | 2254 | 1505 | 749 | 47.4 | 23.5 | 124.7 | 38.9 | 42 | 0 | NA | NA | NA | NA |
| NCT00422058 | 283 | 185 | 98 | 45.5 | 24 | 124.3 | 34.9 | 21 | 4.1 | NA | NA | NA | NA |
| ELIXA | 6063 | 3031 | 3032 | 60.3 | 66.3 | 129.5 | 30.1 | 76.2 | 100 | 22.4 | 7.7 | 22.1 | 100 |
| GetGoal-Duo-2 | 890 | 297 | 593 | 59.8 | 45.3 | NA | 32.2 | NA | 100 | NA | NA | NA | NA |
| GETGOAL-F1 | 484 | 322 | 162 | 56.1 | 44.67 | NA | 32.5 | NA | 100 | NA | NA | NA | NA |
| GETGOAL-L | 495 | 328 | 167 | 57 | 46 | NA | 32.1 | NA | 100 | NA | NA | NA | NA |
| GETGOAL-M | 680 | 510 | 170 | 54.8 | 43.57 | NA | 32.9 | NA | 100 | NA | NA | NA | NA |
| NCT02207374 | 601 | 480 | 121 | 58.5 | 71.5 | 129.2 | 26.4 | NA | 100 | NA | NA | NA | NA |
| PIONEER 1 | 703 | 527 | 176 | 55 | 50.8 | 126.3 | 31.9 | NA | 100 | NA | NA | NA | NA |
| PIONEER 2 | 821 | 411 | 410 | 58 | 50.5 | NA | 32.8 | NA | 100 | NA | NA | NA | NA |
| PIONEER 3 | 1863 | 1396 | 467 | 58 | 52.82 | NA | 32.5 | NA | 100 | NA | NA | NA | 16.4 |
| PIONEER 4 | 711 | 569 | 142 | 56 | 52 | 128.5 | 33.0 | NA | 100 | NA | NA | NA | NA |
| PIONEER 5 | 324 | 163 | 161 | 70 | 48 | NA | 32.4 | NA | 100 | NA | NA | NA | NA |
| PIONEER 6 | 3183 | 1591 | 1592 | 66 | 68.4 | 136 | 32.3 | 93.9 | 100 | 12.2 (II-III) | NA | NA | NA |
| PIONEER 7 | 504 | 253 | 251 | 57.4 | 57 | NA | 31.5 | NA | 100 | NA | NA | NA | NA |
| PIONEER 8 | 731 | 547 | 184 | 61 | 54 | 133 | 31.0 | NA | 100 | NA | NA | NA | NA |
| PIONEER 9 | 243 | 194 | 49 | 59.4 | 78.60 | 127.8 | 25.9 | NA | 100 | NA | NA | NA | NA |
| PIONEER 10 | 458 | 393 | 65 | 58 | 74 | 132 | 26.0 | NA | 100 | NA | NA | NA | NA |
| SELECT | 17604 | 8803 | 8801 | 61.6 | 72.3 | 131 | 33.3 | 81.9 | NA | 24.4 | 8.8 | 76.5 | 82.5 |
| STEP 1 | 1961 | 1306 | 655 | 46.5 | 25.45 | NA | 37.9 | 35.9 | 0 | NA | 2.6 | NA | NA |
| STEP 2 | 1210 | 807 | 403 | 55 | 49.1 | 130 | 35.7 | 70.1 | 100 | NA | NA | NA | 8.2 |
| STEP 4 | 803 | 535 | 268 | 46 | 21 | 127 | 38.4 | 37.1 | 0 | NA | NA | NA | 0.9 |
| STEP 6 | 401 | 300 | 101 | 51 | 63 | 134 | 31.9 | 75 | 25 | NA | NA | NA | NA |
| STEP 10 | 207 | 138 | 69 | 53 | 29 | 131 | 40.7 | 46 | 0 | NA | NA | NA | NA |
| STEP 11 | 150 | 101 | 49 | 39 | 26 | 127 | 31.3 | 23 | 0 | NA | NA | NA | NA |
| STEP-HFpEF DM | 616 | 310 | 306 | 69.5 | 55.7 | 134 | NA | NA | 100 | 17 | NA | NA | NA |
| STEP-HFpEF | 529 | 263 | 266 | 68 | 43.9 | 133.8 | 37 | 83 | 0 | 100 | NA | NA | 22 |
| STEP UP | 1407 | 1206 | 201 | 47 | 26.3 | NA | 39.9 | 41.6 | 0 | NA | NA | NA | NA |
| STEP UP T2D | 512 | 410 | 102 | 56 | 48.2 | 134 | 38.6 | 79.8 | 100 | NA | NA | NA | NA |
| SUSTAIN 1 | 388 | 259 | 129 | 53.7 | 54 | NA | 32.9 | NA | 100 | NA | NA | NA | NA |
| SUSTAIN 2 | 1231 | 820 | 411 | 55.1 | 50.67 | 132.6 | 32.5 | NA | 100 | NA | NA | NA | NA |
| SUSTAIN 3 | 809 | 404 | 405 | 56.6 | 55.3 | 133.5 | 33.8 | NA | 100 | NA | NA | NA | NA |
| SUSTAIN 4 | 1082 | 722 | 360 | 56.5 | 53 | 132.1 | 33.0 | NA | 100 | NA | NA | NA | NA |
| SUSTAIN 5 | 396 | 264 | 132 | 58.8 | 56.1 | 134.8 | 32.2 | NA | 100 | NA | NA | NA | NA |
| SUSTAIN 6 | 3297 | 1648 | 1649 | 64.6 | 60.7 | 135.6 | NA | 92.8 | 100 | 23.6 | NA | 32.5 | 60.5 |
| SUSTAIN 7 | 1201 | 601 | 600 | 55.5 | 55 | 132 | 33.5 | NA | 100 | NA | NA | NA | NA |
| SUSTAIN 8 | 788 | 394 | 394 | 56.6 | 54 | 130.4 | 32.3 | NA | 100 | NA | NA | NA | NA |
| SUSTAIN 9 | 302 | 151 | 151 | 57 | 58.3 | 127.9 | 31.9 | NA | 100 | NA | NA | NA | NA |
| SUSTAIN 10 | 577 | 290 | 287 | 59.5 | 56.7 | NA | 33.7 | NA | 100 | NA | NA | NA | NA |
| SUSTAIN 11 | 1748 | 874 | 874 | 61.2 | 51.1 | 134.4 | 31.5 | 78.7 | 100 | NA | NA | 6.1 | 5.9 |
| SUSTAIN-CHINA MRCT | 868 | 578 | 290 | 53 | 57.5 | 128.8 | 27.8 | 31.8 | 100 | NA | NA | NA | NA |
| SURPASS-AP-Combo | 907 | 687 | 220 | 54.1 | 55.9 | NA | NA | NA | 100 | 0 | NA | NA | NA |
| SURPASS 2 | 1878 | 1409 | 469 | 56.6 | 47 | 130.6 | NA | NA | NA | NA | NA | NA | NA |
| SURPASS 3 | 1444 | 1084 | 360 | 57.4 | 55.9 | 132 | 33.5 | NA | 100 | NA | NA | NA | NA |
| SURPASS 4 | 2002 | 1002 | 1000 | 63.6 | 62.5 | 134.4 | 32.6 | 93 | 100 | 7 | 30 | 32 | 44 |
| SURMOUNT-1 | 2539 | 1896 | 643 | 44.9 | 32.5 | 123.3 | 38 | NA | 0 | NA | NA | NA | NA |
| SURMOUNT-2 | 938 | 623 | 315 | 54.2 | 49 | NA | 36.1 | NA | 100 | NA | NA | NA | NA |
| SURMOUNT 5 | 751 | 376 | 375 | 44.7 | 35.4 | 125.7 | 39.4 | 39.6 | 0 | 0 | 0.4 | NA | 1.5 |
| SURMOUNT-J | 267 | 192 | 75 | 50.8 | 59 | 125.4 | 33.5 | 53 | 0 | NA | NA | 2 | NA |
| SEMA-AP | 28 | 14 | 14 | 45 | 39 | NA | 32.2 | NA | 100 | NA | NA | NA | NA |
| NCT04889183 | 101 | 51 | 50 | 56 | 60 | 131.7 | 36.2 | NA | 0 | NA | NA | NA | NA |
| ATTAIN-1 | 3127 | 2178 | 949 | 45.1 | 35.8 | 125.5 | 37.0 | 39.5 | 0 | NA | NA | NA | 1.5 |
| OASIS-4 | 307 | 205 | 102 | 47.5 | 21.2 | 131.0 | 37.6 | 42.8 | 1.6 | NA | NA | NA | 1.7 |
| MariTide Phase 2 | 592 | 482 | 110 | 49.2 | 41.7 | 127.8 | 37.6 | NA | 21.4 | NA | NA | NA | NA |
| ACHIEVE-1 | 559 | 421 | 138 | 53.4 | 51.9 | 127.7 | 33.0 | NA | 100 | NA | NA | NA | NA |
| REDEFINE 1 | 3417 | 2108 | 705 | 47.0 | 32.4 | 127.0 | 37.9 | 36.3 | 0 | NA | NA | NA | 1.4 |
| REDEFINE 2 | 1206 | 904 | 302 | 56.2 | 53.0 | 130.2 | 36.3 | 73.3 | 100 | NA | NA | NA | 8.7 |
| SOUL | 9650 | 4825 | 4825 | 66.1 | 71.1 | 134.7 | 31.1 | 90.7 | 100 | 23.1 | 15.7 | 40.0 | 70.7 |
| SUMMIT | 731 | 364 | 367 | 65.2 | 46.2 | 128.1 | 38.3 | NA | 48.2 | 100 | NA | NA | 30.0 |
| EECOH-2 | 623 | 415 | 208 | 53.8 | 55.3 | 125.4 | 26.9 | NA | 100 | NA | NA | NA | NA |
| NCT04867785 | 281 | 236 | 45 | 56.2 | 44 | 129.7 | 35.0 | NA | 100 | NA | NA | NA | NA |
| STRIDE | 792 | 396 | 390 | 68.0 | 75.5 | 134 | 28.5 | 88 | 100 | 14 | 100 | 18 | 43 |
| FLOW | 3533 | 1767 | 1766 | 66.6 | 69.7 | 138.7 | 32.0 | NA | 100 | 19.2 | NA | NA | NA |

*Abbreviations: BMI, Body Mass Index; SBP, Systolic Blood Pressure; HTN, Hypertension; DM, Diabetes Mellitus; HF, Heart Failure; PVD, Peripheral Vascular Disease; MI, Myocardial Infarction; IHD, Ischaemic Heart Disease*

# Table S16: Trial Characteristics (MRA)

| **Study Name** | **NCT Number** | **Dose** | **Controls** | **Countries** | **Follow -up (median years)** | **Total Participants** | **Indication** | **AF Capture** |
| --- | --- | --- | --- | --- | --- | --- | --- | --- |
| EMPHASIS-HF | NA | eplerenone | PLA | 26 | 1.75 | 1794 | HFrEF | ECG^1^ |
| J‐EMPHASIS‐HF | NCT01115855 | eplerenone | PLA | Japan | 4.8* | 221 | HFrEF | AE: “atrial fibrillation” |
| FIDELIO-DKD | NCT02540993 | finerenone | PLA | 48 | 2.6* | 5674 | DM w/ ETOD | AE: “atrial fibrillation” |
| ARTS-DN | NCT1874431 | finerenone | PLA | 23 | 0.2 | 823 | DM w/ ETOD | AE: “atrial fibrillation” |
| FIGARO-DKD | NCT02545049 | finerenone | PLA | 48 | 3.4* | 7352 | DM w/ ETOD | AE: “atrial fibrillation” |
| FINEARTS-HF | [NCT04435626](http://clinicaltrials.gov/show/NCT04435626) | finerenone | PLA | 37 | 2.3 | 5986 | HFpEF | ECG |
| Gao - CHF | NA | spironolactone | PLA | China | 0.5 | 116 | HFrEF | ECG |
| TOPCAT | NCT00094302 | spironolactone | PLA | USA, Canada, Brazil, Argentina, Russia, Georgia | 3.1 | 2228 | HFpEF | ECG |
| HOMAGE | NCT02556450 | spironolactone | usual care | France, Germany, Ireland, Italy, Netherlands, UK | 0.8 | 527 | CVRF | ECG |
| CONFIDENCE | NCT05254002 | finerenone | empagliflozin | 14 | 0.5 | 818 | DM w/ ETOD | AE: “atrial fibrillation” |
| ALCHEMIST | NCT01848639 | spironolactone | PLA | France, Belgium, Monaco | 2.72 | 644 | CKD | ECG |
| ACHIEVE | NCT03020303 | spironolactone | PLA | 12 | 1.8 | 2538 | DM w/ ETOD | AE: “atrial fibrillation” |
| NCT04331691 | NCT04331691 | spironolactone | amiloride | South Korea | 0.23 | 118 | HTN | AE: “atrial fibrillation” |

*Abbreviations: PLA, Placebo; CKD, Chronic Kidney Disease; DM w/ ETOD, Diabetes Mellitus w/ End Target Organ Damage; HFpEF, Heart Failure with preserved Ejection Fraction; HFrEF, Heart Failure with reduced Ejection Fraction; HTN, Hypertension; CVRF, Cardiovascular Risk Factors; AE, Adverse Event*

*^1^includes atrial flutter*

**Median value*

# Table S17: Baseline Cohort Characteristics (MRA)

| **Study Name** | **Participants (n)** | | | **Age** | **Male (%)** | **Comorbidity (%)** | | | | | |
| --- | --- | --- | --- | --- | --- | --- | --- | --- | --- | --- | --- |
|  |  |  |  |  |  | **HTN** | **DM** | **HF** | **PVD** | **MI** | **IHD** |
|  | **Total** | **Intervention** | **Control** |  |  |  |  |  |  |  |  |
| EMPHASIS-HF | 1794 | 911 | 883 | 67.9 | 77 | 70.8 | 31.4 | 100 | NA | NA | 68.9 |
| J‐EMPHASIS‐HF | 221 | 111 | 110 | 69 | 79.6 | 6.8 | 39.8 | 100 | NA | 30.5 | NA |
| FIDELIO-DKD | 5674 | 2837 | 2837 | 65.56 | 70.71 | 97.02 | 100 | 8.05 | NA | NA | NA |
| ARTS-DN | 823 | 729 | 94 | 64.2 | 78 | 94.4 | 100 | NA | NA | NA | NA |
| FIGARO-DKD | 7352 | 3686 | 3666 | 64.1 | 69.4 | 96.0 | 100 | 7.8 | NA | NA | NA |
| FINEARTS-HF | 5986 | 2993 | 2993 | 71.9 | 45.5 | 88.9 | 40.7 | 100 | NA | 25.7 | NA |
| Gao - CHF | 116 | 58 | 58 | 54.5 | 64.66 | 58 | 35 | 100 | NA | NA | 63 |
| TOPCAT | 2228 | 1111 | 1117 | 68 | 46.03 | 91.8 | 32.4 | 100 | NA | NA | NA |
| HOMAGE | 527 | 265 | 262 | 73 | 32.5 | 78.2 | 41.5 | 0 | NA | 40.9 | 72.1 |
| CONFIDENCE | 818 | 264 | 267 | 66.5 | 75.2 | 135.2 | 100 | 3.9 | NA | NA | NA |
| ALCHEMIST | 644 | 320 | 324 | 71.3 | 69 | 90 | 69 | NA | 32 | NA | 42 |
| ACHIEVE | 2538 | 1260 | 1278 | 61.7 | 63.3 | NA | 55.6 | 11.5 | NA | 11.9 | NA |
| NCT04331691 | 118 | 60 | 58 | 55 | 70 | 100 | 29.7 | 6.8 | 6 | 0.9 | NA |

*Abbreviations: BMI, Body Mass Index; SBP, Systolic Blood Pressure; HTN, Hypertension; DM, Diabetes Mellitus; HF, Heart Failure; PVD, Peripheral Vascular Disease; MI, Myocardial Infarction; IHD, Ischaemic Heart Disease*

# Table S18: Trial Characteristics (O3FA)

| **Study Name** | **NCT Number** | **Dose** | **Controls** | **Countries** | **Follow-up (median years)** | **Total Participants** | **Indication** | **AF Capture** |
| --- | --- | --- | --- | --- | --- | --- | --- | --- |
| ASCEND | NCT00135226 | n-3 PUFA | PLA | UK | 7.4 | 15341 | DM | AE: “atrial fibrillation” |
| VITAL | NCT02178410 | n-3 PUFA | PLA | USA | 5.3* | 25119 | Open^1^ | Medical Record, 12-lead ECG |
| GISSI-HF | NA | n-3 PUFA | PLA | Italy | 3.9* | 5835 | CHF | 12-lead ECG |
| R&P | NCT00317707 | n-3 PUFA | PLA | Italy | 5* | 12505 | ASCVD | Medical Record |
| REDUCE-IT | NCT02926027 | n-3 PUFA | PLA | 11 | 4.9* | 8179 | CVD / DM | AE: “atrial fibrillation” |
| STRENGTH | NCT02104817 | n-3 PUFA + statin | PLA | 22 | 3.5* | 13078 | HL | 12-lead ECG |
| OMEMI | NCT01841944 | n-3 PUFA | PLA | Norway | 2 | 1014 | AMI | 12-lead ECG, Medical Record |
| ORIGIN | NCT00069784 | n-3 PUFA | PLA | 40 | 6.2* | 12611 | CVD / DM | AE: “atrial fibrillation” |
| RESPECT-EPA | NA | n-3 PUFA + statin | standard statin only | Japan | 4 | 2460 | CAD | 12-lead ECG, Medical Record |
| DO-HEALTH | N[CT01745263](https://clinicaltrials.gov/ct2/show/NCT01745263) | n-3 PUFA | PLA | Switzerland, Germany, Austria, France, Portugal | 3* | 2157 | Elderly | AE: “atrial fibrillation” |

*^1^No pathology stated for inclusion*

*Abbreviations: PLA, Placebo; DM, Diabetes Mellitus; CHF, Congestive Heart Failure; ASCVD, Atherosclerotic Cardiovascular Disease; CVD, Cardiovascular Disease; DM, Diabetes Mellitus; AMI, Acute Myocardial Infarction; CAD, Coronary Artery Disease; HL, Hyperlipidaemia; AE, Adverse Event; *Median value*

# Table S19: Baseline Cohort Characteristics (O3FA)

| **Study Name** | **Participants (n)** | | | **Age** | **Male (%)** | **BMI** | **Smoking (%)** | **Comorbidity (%)** | | | | | |
| --- | --- | --- | --- | --- | --- | --- | --- | --- | --- | --- | --- | --- | --- |
|  | **Total** | **Intervention** | **Control** |  |  |  |  | **HTN** | **DM** | **HF** | **PVD** | **IHD** | **MI** |
| ASCEND | 15341 | 7672 | 7669 | 63 | 63 | 30.7 | 53.8 | 61.6 | 100 | NA | NA | NA | NA |
| VITAL | 25119 | 12542 | 12577 | 66.7 | 49.2 | 27.1 | 48.3 | 51.65 | 13.72 | NA | NA | NA | NA |
| GISSI-HF | 5835 | 2921 | 2914 | NA | 68 | 26.9 | NA | 53.8 | 28.6 | 100 | 8.9 | 52.2 | 44.5 |
| R&P | 12505 | 6239 | 6266 | 64 | 61 | NA | 21.7 | 84.5 | 59.9 | 3.2 | 7.9 | NA | 0 |
| REDUCE-IT | 8179 | 4089 | 4090 | 64 | 71.2 | 30.8 | NA | NA | 59.5 | NA | NA | NA | NA |
| STRENGTH | 13078 | 6539 | 6539 | 62.5 | 65 | 32.2 | NA | 87.3 | 70.1 | NA | 3.7 | 46.1 | NA |
| OMEMI | 1014 | 505 | 509 | 74 | 70 | 27.0 | 11.9 | 60.3 | 20.8 | 6.4 | NA | NA | 25.8 |
| ORIGIN | 12611 | 6319 | 6292 | 64 | 65 | 29.8 | 12.3 | 79.5 | NA | NA | NA | NA | 58.8* |
| RESPECT-EPA | 2460 | 1225 | 1235 | 68 | 82.7 | 24.5 | 13.6 | 83.2 | 45.1 | NA | NA | 54.4 | 100 |
| DO-HEALTH | 2157 | 1073 | 1084 | 74.9 | 38.1 | 26.4 | NA | 49.4 | NA | NA | NA | 0 | NA |

*MI, stroke, or revascularisation.

*Abbreviations: BMI, Body Mass Index; SBP, Systolic Blood Pressure; HTN, Hypertension; DM, Diabetes Mellitus; HF, Heart Failure; PVD, Peripheral Vascular Disease; MI, Myocardial Infarction; IHD, Ischaemic Heart Disease*

# Table S20: Trial Characteristics (SGLT2 Inhibitor)

| **Study Name** | **NCT Number** | **Dose** | **Controls** | **Countries** | **Follow-up (mean years)** | **Total Participants** | **Indication** | **AF Capture** |
| --- | --- | --- | --- | --- | --- | --- | --- | --- |
| NCT00642278 | NCT00642278 | canagliflozin | sitagliptin | 12 | 0.2 | 451 | DM | AE: “atrial fibrillation” |
| CANVAS Program | NCT01032629  NCT01989754 | canagliflozin | PLA | 39 | 3.6 | 10134 | DM with ETOD | AE: “atrial fibrillation” |
| SUSTAIN 8 | [NCT03136484](https://clinicaltrials.gov/show/NCT03136484) | canagliflozin | semaglutide | 11 | 1 | 788 | DM | AE: “atrial fibrillation” |
| DIA3004 | NCT01064414 | canagliflozin | PLA | 19 | 1 | 269 | DM with ETOD | AE: “atrial fibrillation” |
| NCT01106651 | NCT01106651 | canagliflozin | PLA | 17 | 2 | 1428 | DM | AE: “atrial fibrillation” |
| CANTATA-SU | NCT00968812 | canagliflozin | glimepiride | 19 | 1 | 1450 | DM | AE: “atrial fibrillation” |
| NCT01022112 | NCT01022112 | canagliflozin | PLA | Japan | 0.2 | 383 | DM | AE: “atrial fibrillation” |
| The CANTATA-D Trial | NCT01106677 | canagliflozin | sitagliptin/ PLA | 22 | 1.1 | 1284 | DM | AE: “atrial fibrillation” |
| NCT00968812 | NCT00968812 | canagliflozin | glimepiride | 19 | 2 | 1450 | DM | AE: “atrial fibrillation” |
| CREDENCE | NCT02065791 | canagliflozin | PLA | 34 | 2.6 | 4397 | DM with ETOD | AE: “atrial fibrillation” |
| CANTATA-D2 | NCT01137812 | canagliflozin | sitagliptin | 17 | 1.1 | 666 | DM | AE: “atrial fibrillation” |
| NCT01809327 | NCT01809327 | canagliflozin | metformin | 12 | 0.5 | 756 | DM | AE: “atrial fibrillation” |
| [NCT00528879](http://www.clinicaltrials.gov/ct2/show/NCT00528879) | [NCT00528879](http://www.clinicaltrials.gov/ct2/show/NCT00528879) | dapagliflozin | PLA + metformin | 5 | 2 | 1186 | DM | AE: “atrial fibrillation” |
| DELIGHT | NCT02547935 | dapagliflozin | PLA | 9 | 0.5 | 485 | DM with ETOD | AE: “atrial fibrillation” |
| Dapagliflozin Monotherapy in Type 2 Diabetic Patients With Inadequate Glycemic Control by Diet and Exercise | NCT00528372 | dapagliflozin | PLA | US, Canada, Mexico, Russia | 2 | 485 | DM | AE: “atrial fibrillation” |
| Dapagliflozin’s Effects on Glycemia and Cardiovascular Risk Factors in High-Risk Patients With Type 2 DM: A 24-Week, Multicenter, Randomized, Double-Blind, PLA-Controlled Study With a 28-Week Extension | NCT01031680 | dapagliflozin | PLA | 9 | 0.5 | 922 | DM with ETOD | AE: “atrial fibrillation” |
| CompoSIT-R | NCT02532855 | dapagliflozin | sitagliptin | 24 | 0.5 | 614 | DM with ETOD | AE: “atrial fibrillation” |
| DETERMINE-reduced | NCT03877237 | dapagliflozin | PLA | 9 | NA | 313 | HFrEF | AE: “atrial fibrillation” |
| [NCT00673231](http://clinicaltrials.gov/show/NCT00673231) | [NCT00673231](http://clinicaltrials.gov/show/NCT00673231) | dapagliflozin | PLA | 9 | 1.1 | 807 | DM | AE: “atrial fibrillation” |
| NCT01042977 | NCT01042977 | dapagliflozin | PLA | 10 | 1 | 963 | DM with ETOD | AE: “atrial fibrillation” |
| DAPA HF | NCT03036124 | dapagliflozin | PLA | 20 | 1.5 | 4736 | HFrEF | AE: “atrial fibrillation” |
| DECLARE-TIMI58 | NCT01730534 | dapagliflozin | PLA | 33 | 4.2 | 17143 | DM with ETOD | AE: “atrial fibrillation” |
| DEFINE HF | NCT02653482 | dapagliflozin | PLA | US | 0.2 | 263 | HFrEF | AE: “atrial fibrillation” |
| Dapa-CKD | NCT03036150 | dapagliflozin | PLA | 21 | 2.4 | 4298 | CKD | AE: “atrial fibrillation” |
| DETERMINE-preserved | NCT03877224 | dapagliflozin | PLA | 12 | NA | 501 | HFpEF | AE: “atrial fibrillation” |
| PRESERVED-HF | NCT03030235 | dapagliflozin | PLA | US | 0.2 | 324 | HFpEF | AE: “atrial fibrillation” |
| NCT00683878 | NCT00683878 | dapagliflozin | PLA + pioglitazone | 8 | 0.9 | 420 | DM | AE: “atrial fibrillation” |
| NCT01646320 | NCT01646320 | dapagliflozin | PLA + saxagliptin + metformin | 7 | 1 | 320 | DM | AE: “atrial fibrillation” |
| NCT02284893 | NCT02284893 | dapagliflozin | sitagliptin + metformin | 6 | 1 | 461 | DM | AE: “atrial fibrillation” |
| DapaZu | NCT02471404 | dapagliflozin | glimepiride + metformin | Germany, Czech Republic, Hungary, Poland, Slovakia | 1 | 929 | DM | AE: “atrial fibrillation” |
| DURATION-8 | NCT02229396 | dapagliflozin | exenatide | 6 | 0.5 | 694 | DM | AE: “atrial fibrillation” |
| DELIVER | NCT03619213 | dapagliflozin | PLA | 20 | 2.3 | 6253 | HFpEF | AE: “atrial fibrillation” |
| EMPA-REG RENAL | NCT01164501 | empagliflozin | PLA | 15 | 1 | 738 | DM with ETOD | AE: “atrial fibrillation” |
| Empire HF | NCT03198585 | empagliflozin | PLA | Denmark | 0.2 | 190 | HFrEF | AE: “atrial fibrillation” |
| EMPEROR-Preserved | NCT03057951 | empagliflozin | PLA | 23 | 2.2 | 5988 | HFpEF | AE: “atrial fibrillation” |
| EMPA-HEART Cardiolink 6 | NCT02998970 | empagliflozin | PLA | Canada | 0.5 | 97 | DM with ETOD | AE: “atrial fibrillation” |
| NCT03152552 | NCT03152552 | empagliflozin | PLA | 24 | 0.7 | 63 | Heart failure where ejection fraction has not been stipulated | AE: “atrial fibrillation” |
| Long-term treatment with empagliflozin as add-on to oral antiDM therapy in Japanese patients with type 2 DM | NCT01368081 | empagliflozin | usual care | Japan | 1 | 1160 | DM | AE: “atrial fibrillation” |
| EMPA-REG-METSU | NCT01159600 | empagliflozin | PLA | 12 | 0.5 | 669 | DM | AE: “atrial fibrillation” |
| SUGAR-DM-HF | NCT03485092 | empagliflozin | PLA | UK | 0.7 | 105 | HFrEF | AE: “atrial fibrillation” |
| PIONEER 2 | NCT02863328 | empagliflozin | semaglutide | 12 | 1 | 821 | DM | AE: “atrial fibrillation” |
| EMPA-REG MONO | NCT01177813 | empagliflozin | sitagliptin | 9 | 0.5 | 899 | DM | AE: “atrial fibrillation” |
| Ferrannini E | NCT00789035 | empagliflozin | PLA | 13 | 0.25 | 659 | DM | AE: “atrial fibrillation” |
| EMPA-REG OUTCOME | NCT01131676 | empagliflozin | PLA | 42 | 3.1* | 7020 | DM | AE: “atrial fibrillation” |
| EMPA-REG PIO | NCT01210001 | empagliflozin | PLA | 7 | 1.5 | 498 | DM | AE: “atrial fibrillation” |
| EMPA-REG BASAL | NCT01011868 | empagliflozin | PLA | 7 | 1.5 | 494 | DM | AE: “atrial fibrillation” |
| Emperial - preserved | NCT03448406 | empagliflozin | PLA | 11 | 0.2 | 315 | HFpEF | AE: “atrial fibrillation” |
| Emperial - Reduced | NCT03448419 | empagliflozin | PLA | 11 | 0.2 | 311 | HFrEF | AE: “atrial fibrillation” |
| EMPEROR-Reduced | NCT03057977 | empagliflozin | PLA | 20 | 1.3 | 3726 | HFrEF | AE: “atrial fibrillation” |
| NCT00749190 | NCT00749190 | empagliflozin | PLA | 16 | 0.2 | 494 | DM | AE: “atrial fibrillation” |
| NCT01734785 | NCT01734785 | empagliflozin | PLA + linagliptin | 10 | 0.5 | 332 | DM | AE: “atrial fibrillation” |
| EMPA-REG H2H-SU | NCT01167881 | empagliflozin | glimepiride + metformin | 23 | 2 | 1545 | DM | AE: “atrial fibrillation” |
| EMPA-KIDNEY | NCT03594110 | empagliflozin | PLA | 8 | 2* | 686 | CKD | AE: “atrial fibrillation” |
| Combination of Empagliflozin and Linagliptin as Second-Line Therapy in Subjects With Type 2 DM Inadequately Controlled on Metformin | NCT01422876 | empagliflozin | linagliptin | 22 | 2* | 6609 | DM | AE: “atrial fibrillation” |
| VERTIS MET | NCT02033889 | ertugliflozin | PLA | 14 | 2 | 621 | DM | AE: “atrial fibrillation” |
| VERTIS RENAL | NCT01986855 | ertugliflozin | PLA | 13 | 1 | 467 | DM with ETOD | AE: “atrial fibrillation” |
| VERTIS SU | NCT01999218 | ertugliflozin | glimepiride | 16 | 2 | 1315 | DM | AE: “atrial fibrillation” |
| VERTIS CV | NCT01986881 | ertugliflozin | PLA | 34 | 3.5* | 8238 | DM with ETOD | 12-lead ECG |
| VERTIS FACTORIAL | NCT02099110 | ertugliflozin | sitagliptin | 21 | 1 | 1472 | DM | AE: “atrial fibrillation” |
| inTandem2 | NCT02421510 | sotagliflozin | PLA | 18 | 1 | 782 | DM | AE: “atrial fibrillation” |
| SCORED | NCT03315143 | sotagliflozin | PLA | 44 | 1.3* | 10584 | DM with ETOD | AE: “atrial fibrillation” |
| inTandem3 | NCT02531035 | sotagliflozin | PLA | 19 | 0.5 | 1402 | DM | AE: “atrial fibrillation” |
| inTandem1 | NCT02384941 | sotagliflozin | PLA + insulin | 2 | 1 | 793 | DM | AE: “atrial fibrillation” |
| EMPRESS-MI | NCT05020704 | empagliflozin | PLA | United Kingdom | 0.5 | 104 | Vasc. disease | AE: “atrial fibrillation” |
| CAMEO DAPA | NCT04730947 | dapagliflozin | PLA | United States | 0.5 | 43 | HFpEF | AE: “atrial fibrillation” |
| EMPATROPISM-FE | NCT03485222 | empagliflozin | PLA | Germany | 0.5 | 84 | HFrEF | AE: “atrial fibrillation” |

*Abbreviations: PLA, Placebo; DM, Diabetes Mellitus; DM w/ ETOD, Diabetes Mellitus w/ End Target Organ Damage; HFpEF, Heart Failure with preserved Ejection Fraction; HFrEF, Heart Failure with reduced Ejection Fraction; CKD, Chronic Kidney Disease; Vasc. Disease, Vascular Disease; AE, Adverse Event*

**Median value*

# Table S21: Baseline Cohort Characteristics (SGLT2 Inhibitor)

| **Study Name** | **Participants (n)** | | | **Age** | **Male (%)** | **BMI** | **SBP (mmHg)** | **Comorbidity (%)** | | | | | |
| --- | --- | --- | --- | --- | --- | --- | --- | --- | --- | --- | --- | --- | --- |
|  | **Total** | **Intervention** | **Control** |  |  |  |  | **HTN** | **DM** | **HF** | **PVD** | **MI** | **IHD** |
| NCT00642278 | 451 | 321 | 130 | 52.9 | 52 | 31.5 | 126.8 | NA | 100 | NA | NA | NA | NA |
| CANVAS Program | 10134 | 5790 | 4344 | 63.3 | 64.2 | 32.0 | 136.6 | 90.0 | 100 | 14.4 | 20.8 | NA | 56.4 |
| SUSTAIN 8 | 788 | 394 | 394 | 56.6 | 54 | 32.3 | 130.4 | NA | 100 | NA | NA | NA | NA |
| DIA3004 - NCT01064414 | 269 | 179 | 90 | 68.5 | 60.6 | 33.0 | NA | NA | 100 | NA | NA | NA | NA |
| NCT01106651 | 1428 | 954 | 474 | 63.6 | 55.5 | 31.6 | 234 | NA | 100 | NA | NA | NA | NA |
| CANTATA-SU | 1450 | 968 | 482 | 56.2 | 52 | 31.0 | 130 | NA | 100 | NA | NA | NA | NA |
| NCT01022112 | 383 | 308 | 75 | 57.4 | 68.1 | 25.7 | NA | 44 | 100 | NA | NA | NA | NA |
| The CANTATA-D Trial | 1284 | 735 | 549 | 55.4 | 47.1 | 31.8 | 128.2 | NA | 100 | NA | NA | NA | NA |
| NCT00968812 | 1450 | 968 | 482 | 56.2 | 52.1 | 31.0 | 130 | NA | 100 | NA | NA | NA | NA |
| CREDENCE | 4397 | 2200 | 2197 | 63 | 66.1 | 31.3 | 140.0 | 96.8 | 100 | 14.8 | NA | NA | NA |
| Effects of Canagliflozin on Cardiovascular Biomarkers in Older Adults With T2DM | 666 | 450 | 216 | 63.7 | 57.21 | 31.6 | 130.0 | 77.3 | 100 | NA | NA | NA | NA |
| CANTATA-D2 | 756 | 378 | 378 | 56.7 | 55.9 | 31.6 | 130.6 | 0 | 100 | 0 | 0 | 0 | 0 |
| NCT01809327 | 1186 | 949 | 237 | 54.9 | 48 | 32.5 | 127.8 | NA | 100 | NA | NA | NA | NA |
| [NCT00528879](http://www.clinicaltrials.gov/ct2/show/NCT00528879) | 485 | 410 | 75 | 54.6 | 53.4 | 31.5 | 127 | NA | 100 | NA | NA | NA | NA |
| Dapagliflozin Monotherapy in Type 2 Diabetic Patients With Inadequate Glycemic Control by Diet and Exercise | 485 | 410 | 75 | 51.8 | 52.16 | 32 | NA | NA | 100 | NA | NA | NA | NA |
| NCT01031680 | 922 | 461 | 461 | 62.9 | 68.25 | 32.7 | 133.2 | 100 | 100 | 0 | 3.6 | NA | 75.1 |
| CompoSIT-R | 614 | 307 | 307 | 67.1 | 57.9 | 31.6 | 127 | NA | 100 | NA | NA | NA | NA |
| DETERMINE-reduced | 313 | 156 | 157 | 69 | 74.5 | 28.5 | NA | NA | NA | 100 | NA | NA | NA |
| [NCT00673231](http://clinicaltrials.gov/show/NCT00673231) | 807 | 610 | 197 | 59.3 | 47.75 | 33.1 | NA | 85.6 | 100 | NA | 10.5 | NA | 18.9 |
| NCT01042977 | 963 | 480 | 483 | 63.7 | 67 | 32.8 | 135 | 92.8 | 100 | 15.8 | NA | NA | NA |
| DAPA HF | 4736 | 2368 | 2368 | 66.3 | 76.6 | 28.1 | NA | NA | 41.8 | 100 | NA | NA | NA |
| DECLARE-TIMI58 | 17143 | 8574 | 8569 | 64 | 63 | 32.1 | 134.9 | NA | 100 | 10.1 | 6.0 | NA | 32.9 |
| DEFINE HF | 263 | 131 | 132 | 61.3 | 73.35 | NA | 123.5 | NA | 63.1 | 100 | NA | NA | 52.9 |
| Dapa-CKD | 4298 | 2149 | 2149 | 61.9 | 66.9 | 29.4 | 137.0 | NA | 67.5 | 10.8 | NA | NA | NA |
| DETERMINE-preserved | 501 | 252 | 249 | 73 | 63.4 | 28.5 | NA | NA | NA | 100 | NA | NA | NA |
| PRESERVED-HF | 324 | 162 | 162 | 70 | 43 | 34.8 | 133 | NA | 55.9 | 100 | NA | NA | 31.5 |
| NCT00683878 | 420 | 281 | 139 | 53.5 | 50.5 | NA | NA | NA | 100 | NA | NA | NA | NA |
| NCT01646320 | 320 | 160 | 160 | 55.1 | 45.6 | 31.7 | NA | NA | 100 | NA | NA | NA | NA |
| NCT02284893 | 461 | 232 | 229 | 55.9 | 45.6 | 33.1 | 129.3 | NA | 100 | NA | 0 | 0 | 0 |
| DapaZu | 929 | 616 | 313 | 58.4 | 63.9 | 32.9 | 138.8 | 80.9 | 100 | NA | NA | NA | NA |
| DURATION-8 | 694 | 464 | 230 | 54 | 48 | 32.7 | 130.0 | NA | 100 | NA | NA | NA | NA |
| DELIVER | 6253 | 3126 | 3127 | 71.6 | 56.1 | 29.9 | 128.2 | 88.7 | 44.8 | 100 | NA | 26.7 | 100 |
| EMPA-REG RENAL | 738 | 419 | 319 | 64.9 | 57 | 31.5 | 135.6 | NA | 100 | NA | NA | NA | NA |
| Empire HF | 190 | 95 | 95 | 64 | 85 | 29 | NA | NA | 17.5 | 100 | NA | NA | 51.5 |
| EMPEROR-Preserved | 5988 | 2997 | 2991 | 71.8 | 55.4 | 29.84 | 131.8 | 90.6 | 49.1 | 100 | NA | NA | NA |
| EMPA-HEART Cardiolink 6 | 97 | 49 | 48 | 64 | 93 | 26.6 | 131 | 91 | 100 | 6 | 2.5 | 20 | NA |
| NCT03152552 | 63 | 30 | 33 | NA | NA | NA | NA | NA | NA | 100 | NA | NA | NA |
| Long-term treatment with empagliflozin as add-on to oral antiDM therapy in Japanese patients with type 2 DM | 1160 | 1097 | 63 | 60.3 | 71.98 | 25 | 132.5 | NA | 100 | NA | NA | NA | NA |
| EMPA-REG METSU | 669 | 444 | 225 | 57.1 | 51 | 28.1 | 128.9 | NA | 100 | NA | NA | NA | NA |
| SUGAR-DM-HF | 105 | 52 | 53 | 68.7 | 73.3 | 30.7 | 128.0 | 70.5 | 78.1 | 100 | NA | 83.8 | 70.5 |
| PIONEER 2 | 821 | 410 | 411 | 58 | 50.5 | 32.8 | NA | NA | 100 | NA | NA | NA | NA |
| EMPA-REG MONO | 899 | 447 | 452 | 55 | 61 | 28.2 | 131.4 | NA | 100 | NA | NA | NA | NA |
| Ferrannini E | 659 | 547 | 112 | 58 | 52 | 28.5 | 131.2 | NA | 100 | NA | NA | NA | NA |
| EMPA-REG OUTCOME | 7020 | 4687 | 2333 | 63.1 | 71.3 | 30.6 | 135.4 | NA | 100 | 10.0 | 21 | NA | 76 |
| EMPA-REG PIO | 498 | 333 | 165 | 54.5 | 48.4 | 29.2 | 126.1 | NA | 100 | NA | NA | NA | NA |
| EMPA-REG BASAL | 494 | 324 | 170 | 58.8 | 56 | 32.2 | NA | NA | 100 | NA | NA | NA | NA |
| Emperial - Preserved | 315 | 157 | 158 | 74 | 56.8 | 29.6 | 130 | NA | 51.1 | 100 | NA | NA | NA |
| Emperial - Reduced | 311 | 155 | 156 | 69.5 | 74.4 | 29.6 | 123 | NA | 59.9 | 100 | NA | NA | NA |
| EMPEROR-Reduced | 3726 | 1863 | 1863 | 66.85 | 76.05 | 27.9 | 122.0 | 72.3 | 49.8 | 100 | NA | NA | NA |
| NCT00749190 | 494 | 423 | 71 | NA | NA | NA | NA | NA | 100 | NA | NA | NA | NA |
| NCT01734785 | 332 | 222 | 110 | 55.2 | 59.6 | 30.2 | 130.5 | NA | 100 | NA | NA | NA | NA |
| EMPA-REG H2H-SU | 1545 | 765 | 780 | 57.2 | 57 | 30.39 | 133.4 | NA | 100 | NA | NA | NA | NA |
| Combination of Empagliflozin and Linagliptin as Second-Line Therapy in Subjects With Type 2 DM Inadequately Controlled on Metformin | 686 | 558 | 128 | 55.23 | 52.77 | 30.7 | 130.7 | NA | 100 | NA | NA | NA | NA |
| EMPA-KIDNEY | 6609 | 3304 | 3305 | 63.8 | 66.8 | 29.7 | 136.5 | 22* | 96.6 | 10 | 7 | NA | NA |
| VERTIS MET | 621 | 412 | 209 | 56.6 | 46.4 | 31.4 | 130.1 | NA | 100 | NA | NA | NA | NA |
| VERTIS RENAL | 467 | 313 | 154 | 67.3 | 49.5 | 32.5 | NA | NA | 100 | 49.7 | NA | NA | NA |
| VERTIS SU | 1315 | 880 | 435 | 58.2 | 48.8 | 31.4 | 130.3 | NA | 100 | NA | NA | NA | NA |
| VERTIS CV | 8238 | 5493 | 2745 | 64.4 | 69.8 | 31.9 | 133.3 | NA | 100 | 23.9 | 18.7 | 48.1 | 76.1 |
| VERTIS FACTORIAL | 1472 | 985 | 487 | 53.86 | 53.86 | 32 | 129.2 | NA | 100 | NA | NA | NA | NA |
| inTandem2 | 782 | 524 | 258 | 41.2 | 51.9 | 27.77 | 123.1 | 32.1 | 100 | NA | NA | NA | NA |
| SCORED | 10584 | 5292 | 5292 | 69 | 55.1 | 31.8 | 138.5 | NA | 100 | 31 | NA | 19.9 | NA |
| inTandem3 | 1402 | 699 | 703 | 42.9 | 49.7 | 28.20 | 121.9 | NA | 100 | NA | NA | NA | NA |
| inTandem1 | 793 | 525 | 268 | 46.1 | 48.3 | 29.66 | 120.1 | 23.2 | 100 | NA | NA | NA | NA |
| EMPRESS-MI | 105 | 51 | 54 | 63.0 | 86.5 | NA | 114.3 | 33.7 | 8.5 | NA | NA | 100 | NA |
| CAMEO DAPA | 43 | 17 | 21 | 67 | 34 | 34.7 | NA | 63 | 17 | 100 | NA | NA | NA |
| EMPATROPISM-FE | 80 | 40 | 40 | 62 | 60 | NA | NA | NA | NA | 100 | NA | NA | NA |

*Abbreviations: BMI, Body Mass Index; SBP, Systolic Blood Pressure; HTN, Hypertension; DM, Diabetes Mellitus; HF, Heart Failure; PVD, Peripheral Vascular Disease; MI, Myocardial Infarction; IHD, Ischaemic Heart Disease*

# Table S22: Trial Characteristics (Statin)

| **Study Name** | **NCT Number** | **Dose** | **Controls** | **Countries** | **Follow-up (mean years)** | **Total Participants** | **Indication** | **AF Capture** |
| --- | --- | --- | --- | --- | --- | --- | --- | --- |
| ASCOT-LLA | NA | atorvastatin | PLA | Scandinavia, UK, Ireland | 3.3* | 10305 | HTN | AE: “atrial fibrillation” |
| ALLIANCE | NA | atorvastatin | usual care | USA | 4.3 | 2442 | Vasc. disease | AE: “atrial fibrillation” |
| CARDS | NA | atorvastatin | PLA | UK, Ireland | 3.9* | 2838 | DM | AE: “atrial fibrillation” |
| 4D | NA | atorvastatin | PLA | Germany | 3.9* | 1255 | CKD | AE: “atrial fibrillation” |
| ASPEN | NA | atorvastatin | PLA | 14 | 4.3 | 2410 | DM | AE: “atrial fibrillation” |
| Sola | NA | atorvastatin | usual care | USA | 1 | 108 | HFrEF | AE: “atrial fibrillation” |
| Vrtovec | NA | atorvastatin | usual care | Slovenia | 1 | 110 | HFrEF | AE: “atrial fibrillation” |
| LEADe | NCT00053599 | atorvastatin | PLA | 10 | 1.5 | 640 | Dementia | AE: “atrial fibrillation” |
| SPARCL | NCT00147602 | atorvastatin | PLA | 25 | 4.9* | 4731 | Vasc. disease | 12-lead ECG |
| Intensive Vs. Standard h Atorvastatin on Calcified Coronary Atherosclerosis | NA | atorvastatin | atorvastatin | 3 | 1 | 471 | Cardiovascular risk factors | AE: “atrial fibrillation” |
| AFCAPS/ TexCAPs | NA | lova­statin | PLA + low-sat fat, low-chol diet | USA | 5.2 | 6605 | Vasc. disease | AE: “atrial fibrillation” |
| ALLHAT | NCT00000542 | pravastatin | PLA | USA, Canada, Mexico | 4.9 | 8582 | HTN | ECG |
| WOSCOP | NA | pravastatin |  | Scotland | 4.9 | 6594 | HC, no known vasc. disease | ECG |
| PROSPER | NA | pravastatin | PLA | NLD, Scotland, Ireland | 3.2 | 5804 | Vasc. disease | AE: “atrial fibrillation” |
| GISSI-P | NA | pravastatin | PLA | Italy | 1.9 | 4271 | Vasc. disease | AE: “atrial fibrillation” |
| PREVEND IT | NA | pravastatin | PLA | Netherlands | 3.8 | 864 | CKD | AE: “atrial fibrillation” |
| MEGA | NCT00211705 | pravastatin | PLA | Japan | 5.3 | 7832 | HC no known vasc. disease | AE: “atrial fibrillation” |
| LIPID | NA | pravastatin | PLA | Australia, NZ | 8 | 9014 | HC, no known vasc. disease | Unclear |
| Pravastatin Therapy and the Risk of Stroke | NA | pravastatin | PLA | USA | 6 | 9014 | Vasc. disease | Unclear |
| GISSI-HF | NCT00336336 | rosuvastatin | PLA | Italy | 3.7* | 1868 | HFpEF | 12-lead ECG |
| GISSI-HF | NCT00336336 | rosuvastatin | PLA | Italy | 3.7* | 1822 | HFrEF | 12-lead ECG |
| METEOR | NCT00225589 | rosuvastatin | PLA | US/Europe | 2 | 984 | HC no known vasc. disease | AE: “atrial fibrillation” |
| JUPITER | NCT00239681 | rosuvastatin | PLA | 26 | 1.8* | 17802 | Elevated CRP | AE: “atrial fibrillation” |
| Heart Protection Study | NA | simvastatin | PLA | UK | 5 | 20536 | Vasc. disease | AE: “atrial fibrillation” |
| SEAS | NCT00092677 | simvastatin+ ezetimibe | PLA | 7 | 4.4* | 1873 | Aortic stenosis | AE: “atrial fibrillation” |

*Abbreviations: PLA, Placebo; DM, Diabetes Mellitus; DM w/ ETOD, Diabetes Mellitus w/ End Target Organ Damage; HFpEF, Heart Failure with preserved Ejection Fraction; HFrEF, Heart Failure with reduced Ejection Fraction; HTN, Hypertension; Vasc. Disease, Vascular Disease; CKD, Chronic Kidney Disease; HC, Hypercholesterolaemia; CRP, C-reactive peptide*

**Median value*

# Table S23: Baseline Cohort Characteristics (Statin)

| **Study Name** | **Participants (n)** | | | **Age** | **Male (%)** | **Smoking (%)** | **Comorbidity (%)** | | | | | | | | |
| --- | --- | --- | --- | --- | --- | --- | --- | --- | --- | --- | --- | --- | --- | --- | --- |
|  | **Total** | **Intervention** | **Control** |  |  |  | **HTN** | **DM** | | **HF** | **PVD** | | **MI** | | **IHD** |
| ASCOT-LLA | 10305 | 5168 | 5137 | 63.2 | 81.2 | 32.7 | 100 | 24.6 | | NA | 5.0 | | NA | | NA |
| ALLIANCE | 2442 | 1,217 | 1,225 | 61.2 | 82.2 | 19.4 | NA | 22.2 | | 6.6 | 3.8^3^ | | 57.6 | | 6.5 |
| CARDS | 2838 | 1428 | 1410 | 62 | 68 | 67 | 84 | 100 | | NA | NA | | NA | | NA |
| 4D | 1255 | 619 | 636 | 65.7 | 46 | 35.5 | NA | 100 | | 35.4 | 44.6 | | 17.6 | | 35.4 |
| ASPEN | 2410 | 1211 | 1199 | 61.1 | 66 | 12.4 | 55 | 100 | | NA | 8.6 | | 16.4 | | NA |
| Sola | 108 | 54 | 54 | 53.7 | 63 | NA | NA | NA | | 100 | NA | | NA | | NA |
| Vrtovec - SCD in CHF | 110 | 55 | 55 | 63 | 61 | NA | NA | NA | | 100 | NA | | NA | | 59 |
| LEADe | 640 | 314 | 326 | 73.8 | 48 | NA | NA | NA | | NA | NA | | NA | | NA |
| SPARCL | 4731 | 2365 | 2366 | 63 | 59.8 | 19.1 | 61.8 | 16.7 | | NA | NA | | NA | | NA |
| Schermund - Calcified Coronary Atherosclerosis | 471 | 236 | 235 | 61.5 | 74.5 | 73.5 | 83 | 13 | | 0 | NA | | NA | | NA |
| AFCAPS/TexCAPs | 6605 | 3304 | 3301 | 58 | 85 | 12.5 | 21.9 | 1.7^2^ | | NA | NA | | NA | | NA |
| ALLHAT | 8582 | 4,327 | 4,255 | 67 | 47 | 21.8 | 100 | 35.5 | | 0 | 46.9^1^ | |  | | 25.7 |
| WOSCOP | 6594 | 3302 | 3292 | 55.2 | 100 | 78 | 15.5 | 1 | | NA | NA | | NA | | NA |
| PROSPER | 5804 | 2891 | 2913 | 75.4 | 48.3 | 66.2 | 63.1 | 11.0 | | NA | 12.4 | | 17.2 | | 34.2 |
| GISSI-P | 4271 | 2138 | 2133 | 65 | 86.3 | 71.9 | 36.6 | 13.6 | | NA | NA | | 16.2 | | NA |
| PREVEND IT | 864 | 433 | 431 | 51.3 | 64.9 | 36.4 | NA | 2.6 | | 0 | 0.6 | | 0.5 | | 0.8^4^ |
| MEGA | 7832 | 3866 | 3966 | 58.3 | 68.5 | 21.5 | 42 | 21 | | NA | NA | | NA | | NA |
| LIPID | 9014 | 4512 | 4502 | 62 | 83 | 73 | 41.5 | 7.5 | | NA | 9.5 | | 64 | | 45^4^ |
| White - Pravastatin Therapy and the Risk of Stroke | 9014 | 4512 | 4502 | 62 | 83 | 73.5 | 41 | 9 | | NA | 10 | | 64 | | 41^4^ |
| GISSI-HFr | 1868 | 950 | 918 | 67 | 77.7 | NA | 53.3 | 26.5 | 100 | | | 7.1 | | 35.4 | NA |
| GISSI-HFp | 1822 | 905 | 917 |  |  |  |  |  |  |  |  |  |  |  |  |
| METEOR | 984 | 702 | 282 | 57 | 60 | NA | NA | 0.3 | | NA | NA | | NA | | NA |
| JUPITER | 17802 | 8901 | 8901 | 66 | 38.2 | 15.8 | NA | 0 | | NA | NA | | NA | | NA |
| Heart Protection Study | 20536 | 10269 | 10267 | NA | 84 | NA | 41 | 19.3 | | NA | 13.1 | | 41 | | 65 |
| SEAS | 1873 | 944 | 929 | 67.6 | 61.4 | 54.3 | 51.5 | 0 | | NA | 0 | | 0 | | 0 |

^1^atherosclerotic cardiovascular disease

^2^non-insulin dependent DM

^3^peripheral revascularisation

^4^coronary angioplasty/bypass

*Abbreviations: BMI, Body Mass Index; SBP, Systolic Blood Pressure; HTN, Hypertension; DM, Diabetes Mellitus; HF, Heart Failure; PVD, Peripheral Vascular Disease; MI, Myocardial Infarction; IHD, Ischaemic Heart Disease*

# Table S24: Risk of bias of ACEI trials.


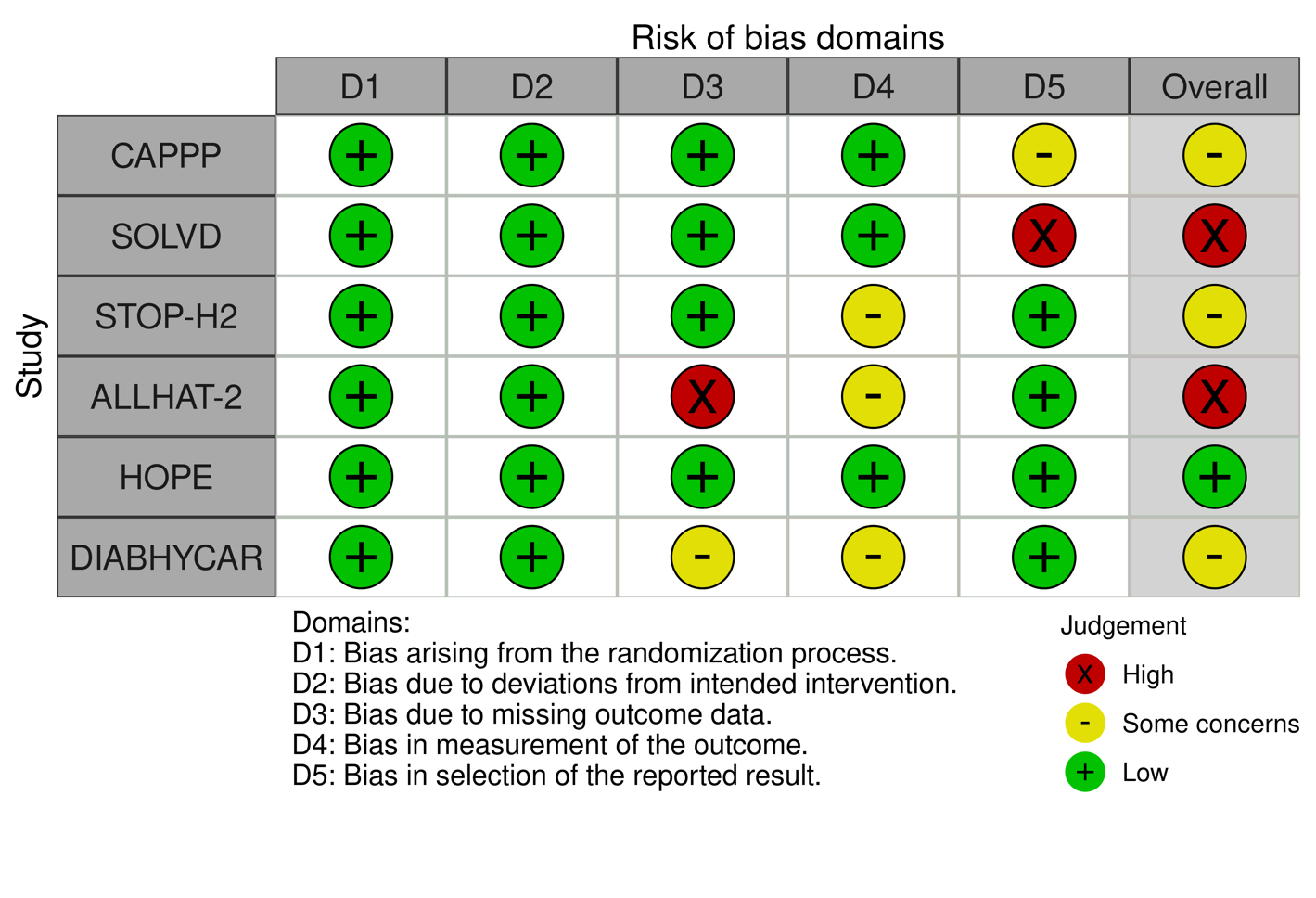


# Table S25: Risk of bias of ARB trials.


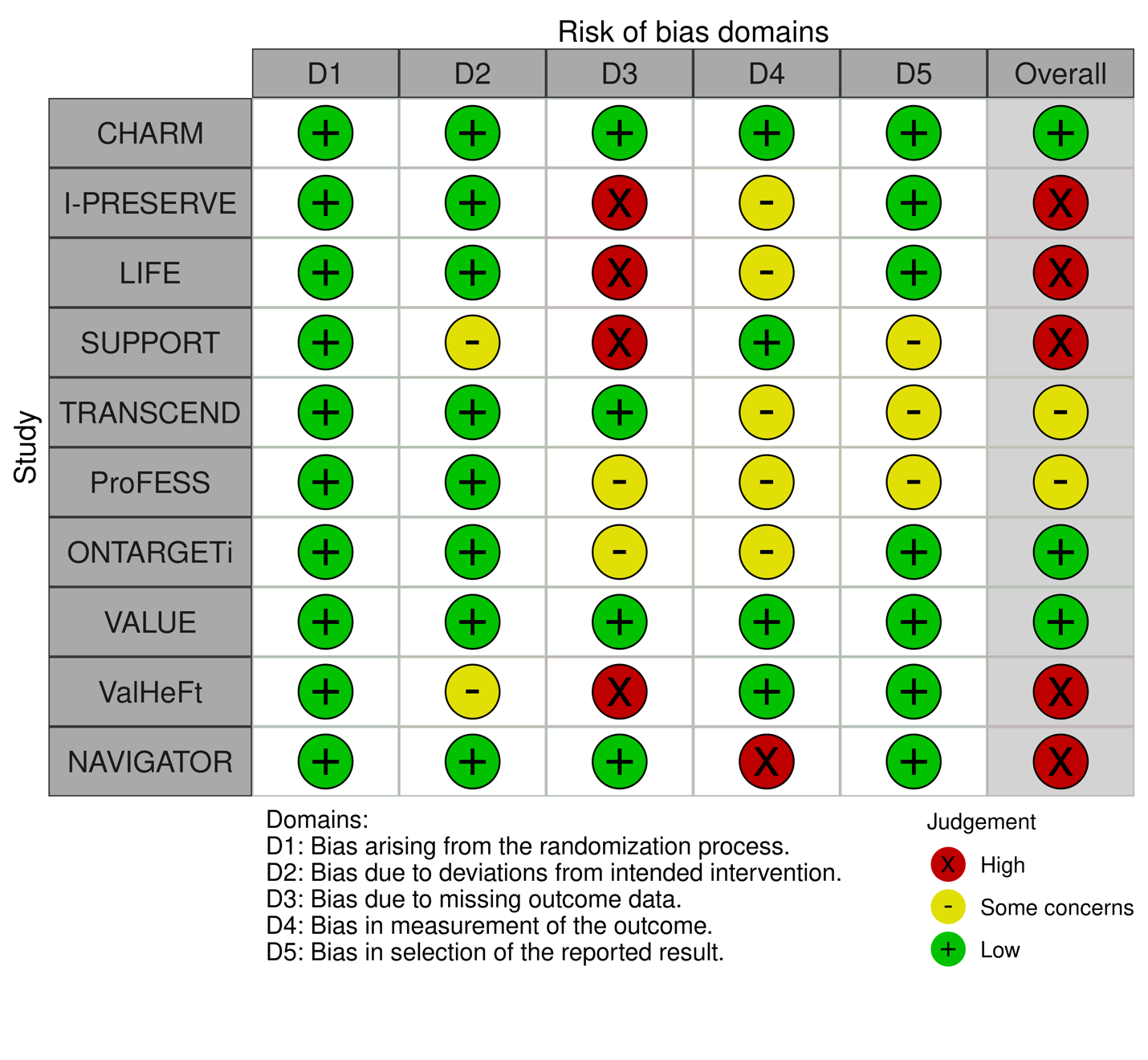


# Table S26: Risk of bias of ARNI trials.


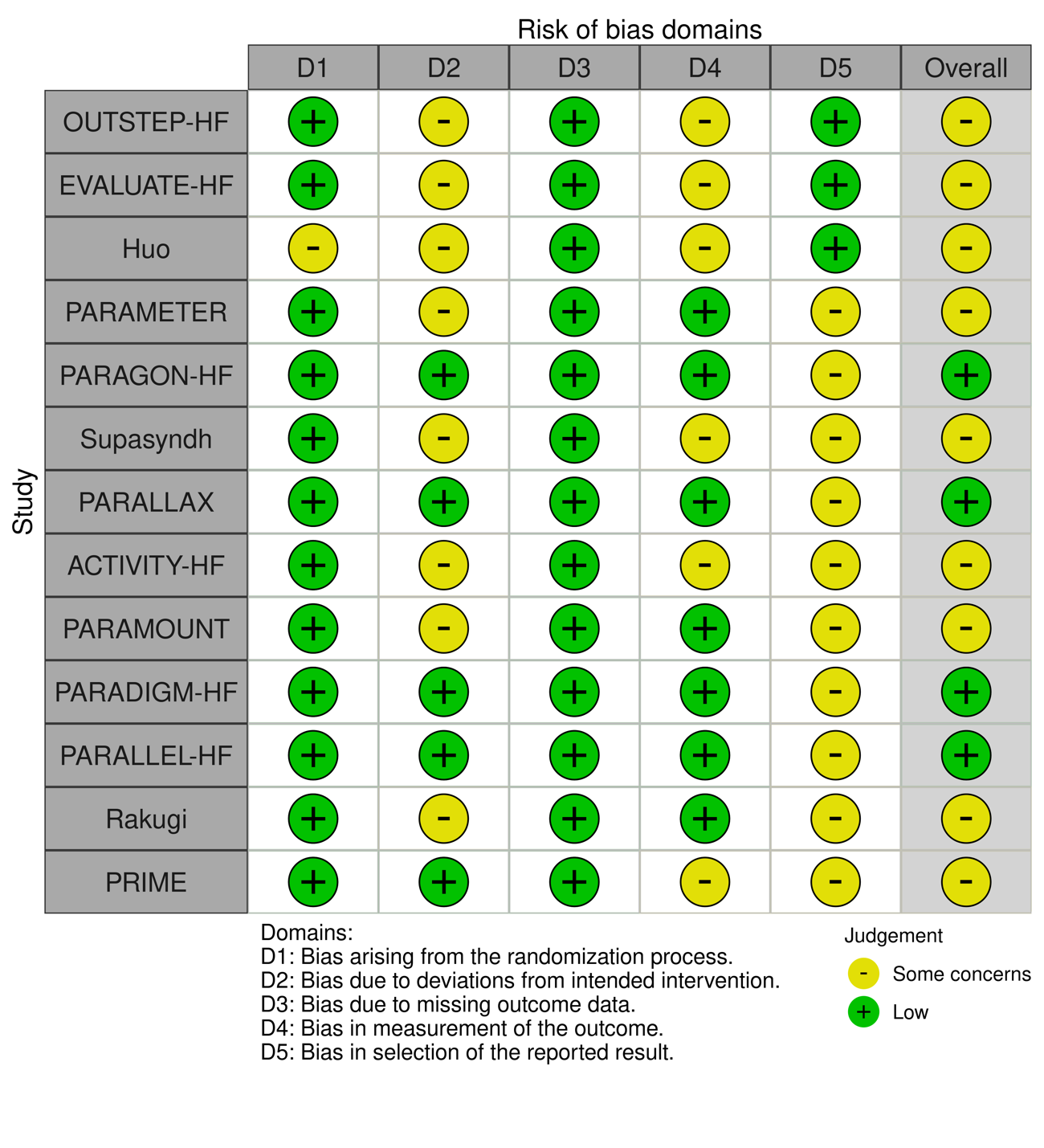


# Table S27: Risk of bias of DPP-4 Inhibitor trials.


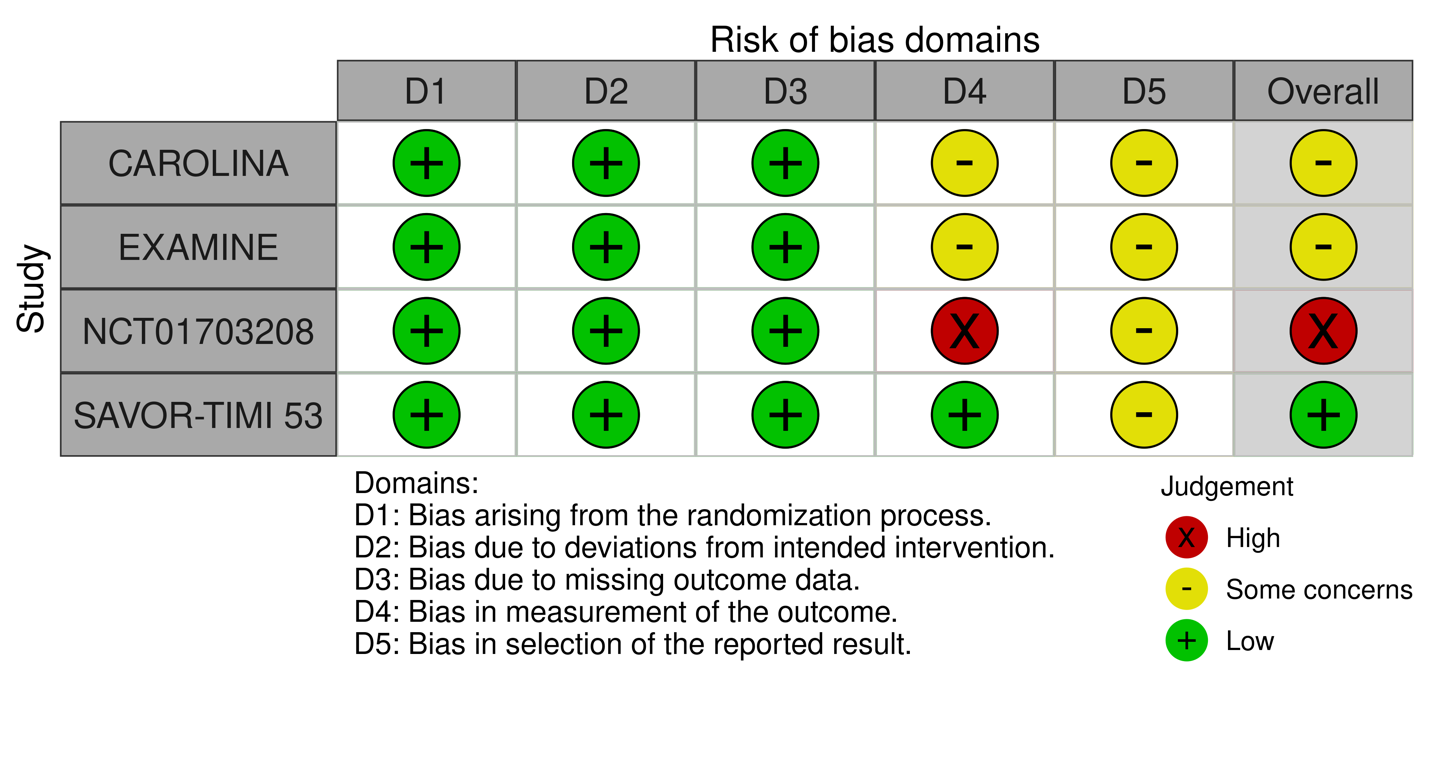


# Table S28: Risk of bias of GLP-1 RA trials.


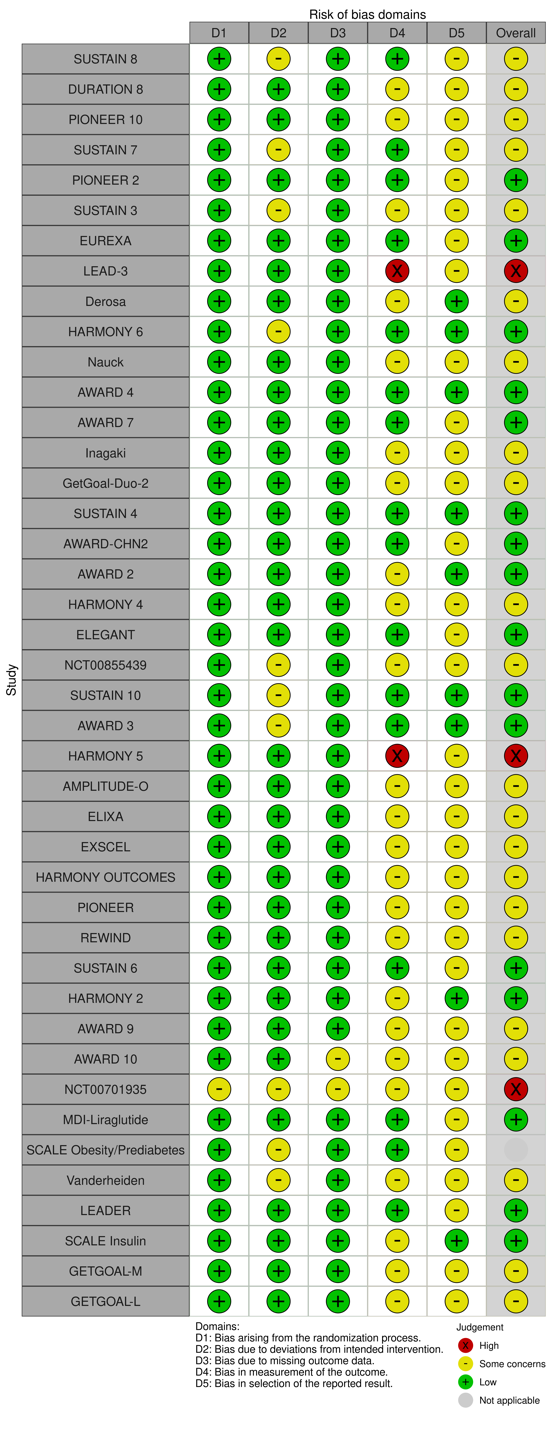


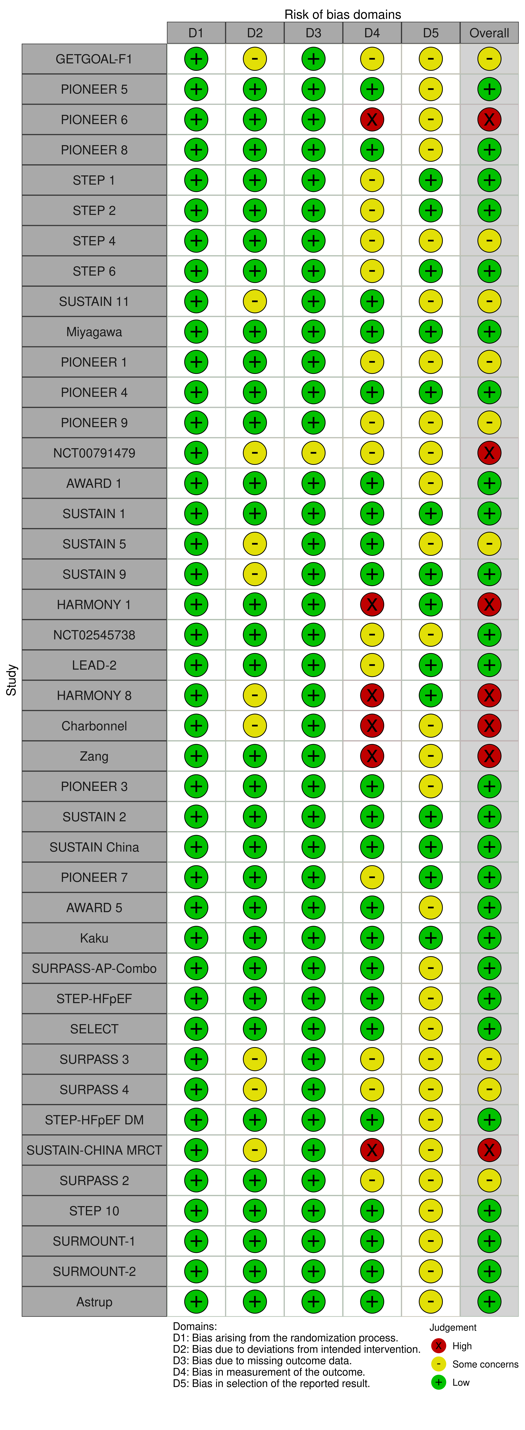


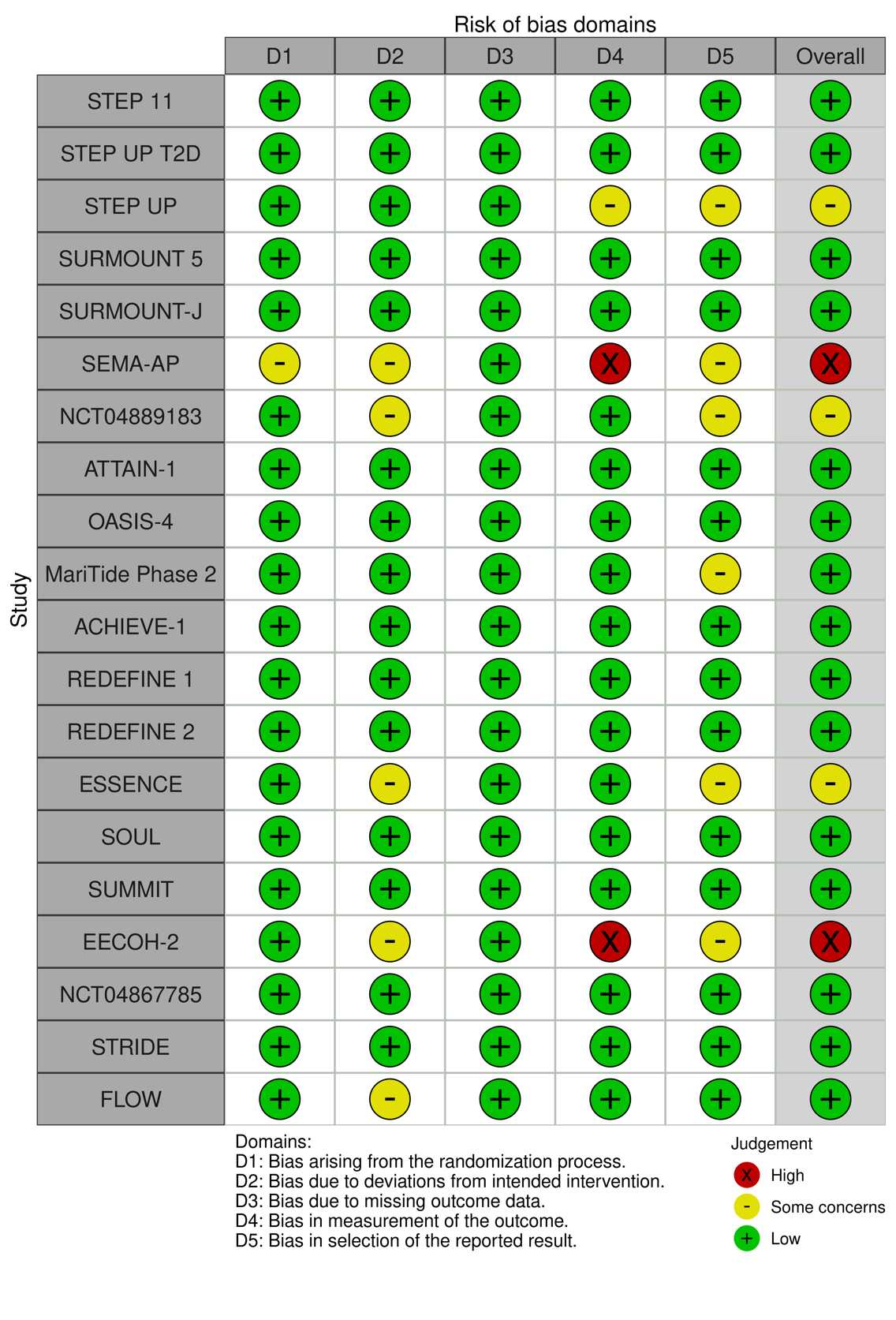


# Table S29: Risk of bias of MRA trials.


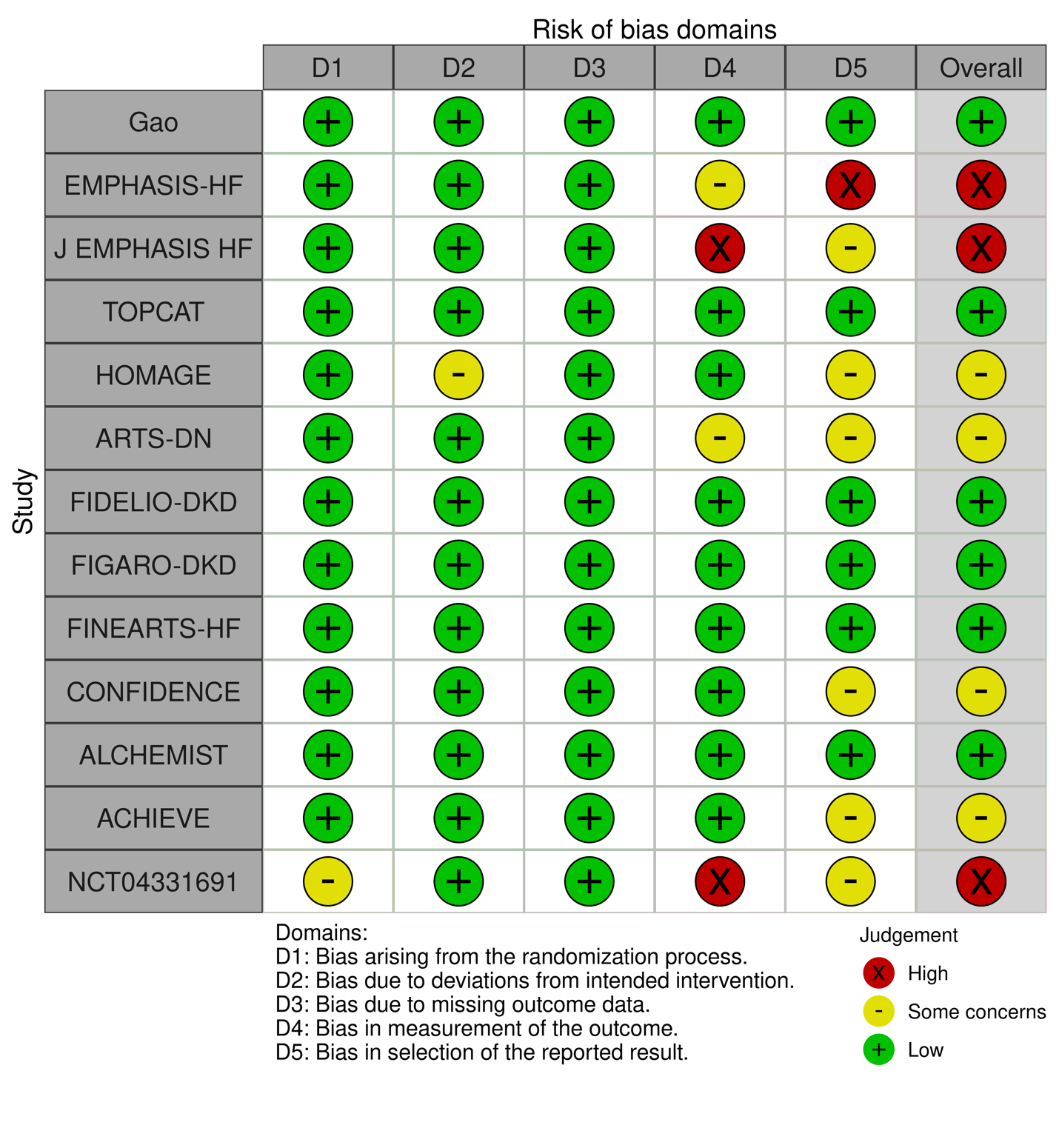


# Table S30: Risk of bias of O3FA trials.


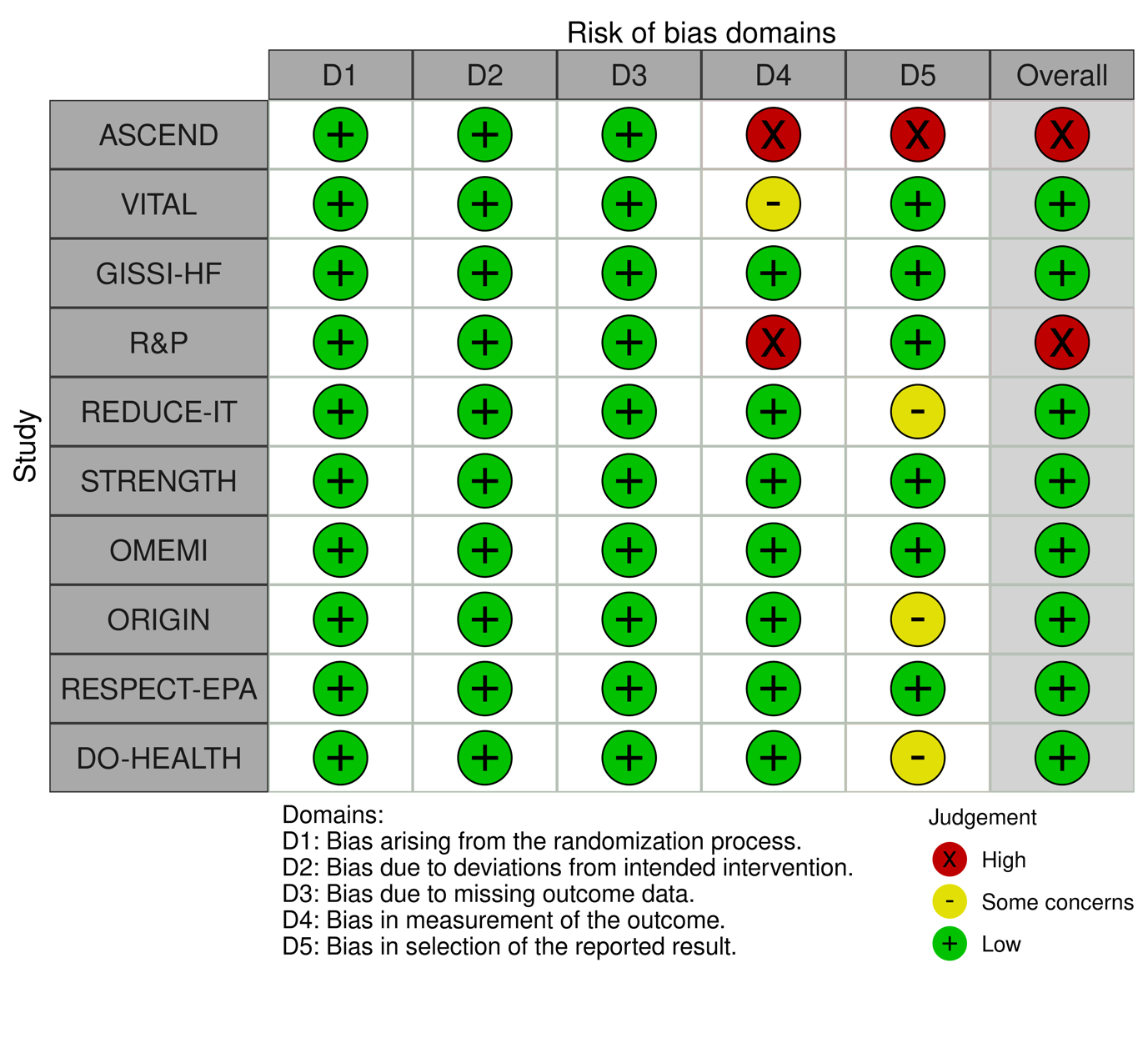


# Table S31: Risk of bias of SGLT2 Inhibitor trials.


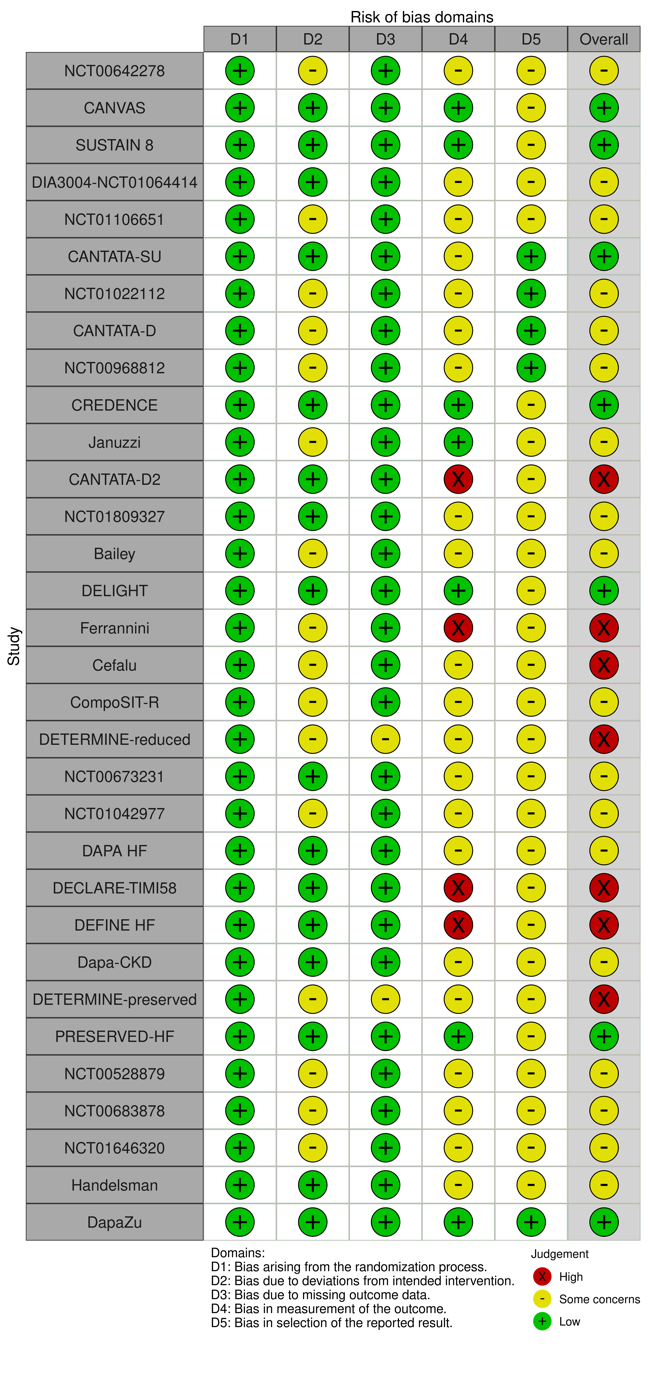


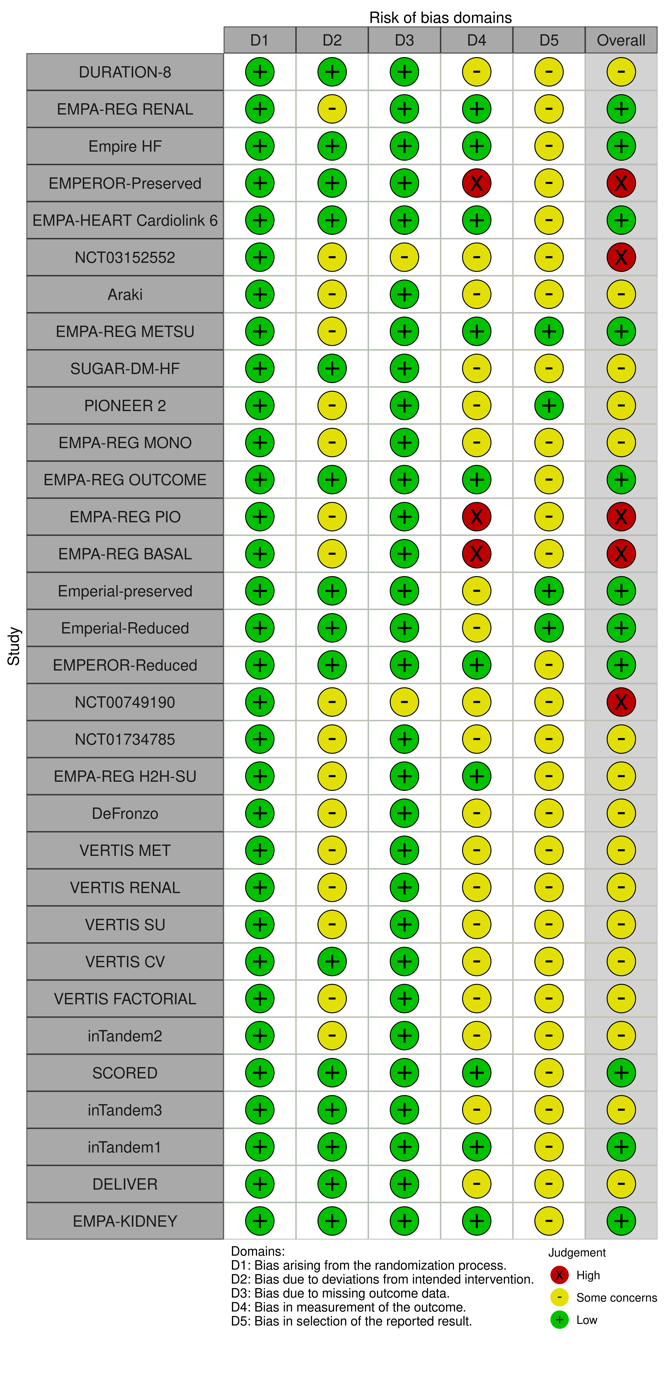


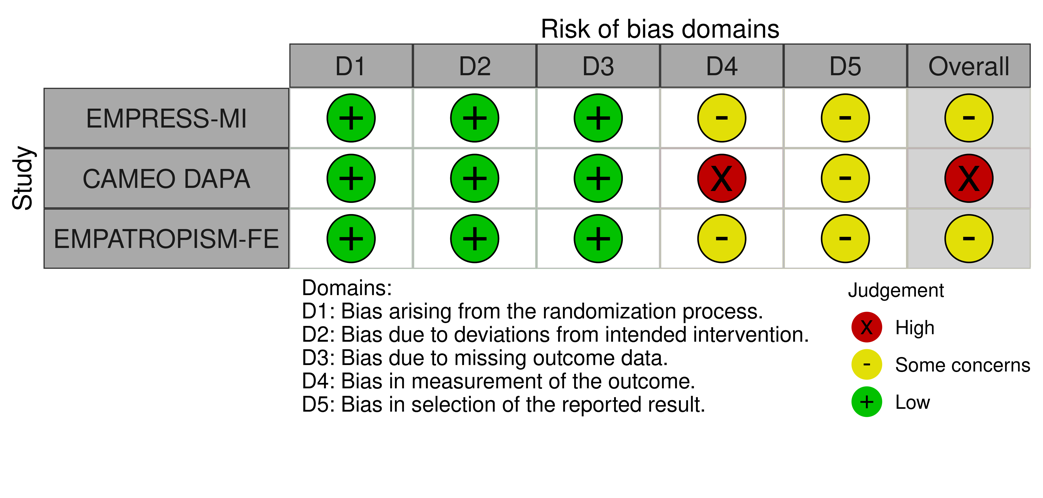


# Table S32: Risk of bias of statin trials.


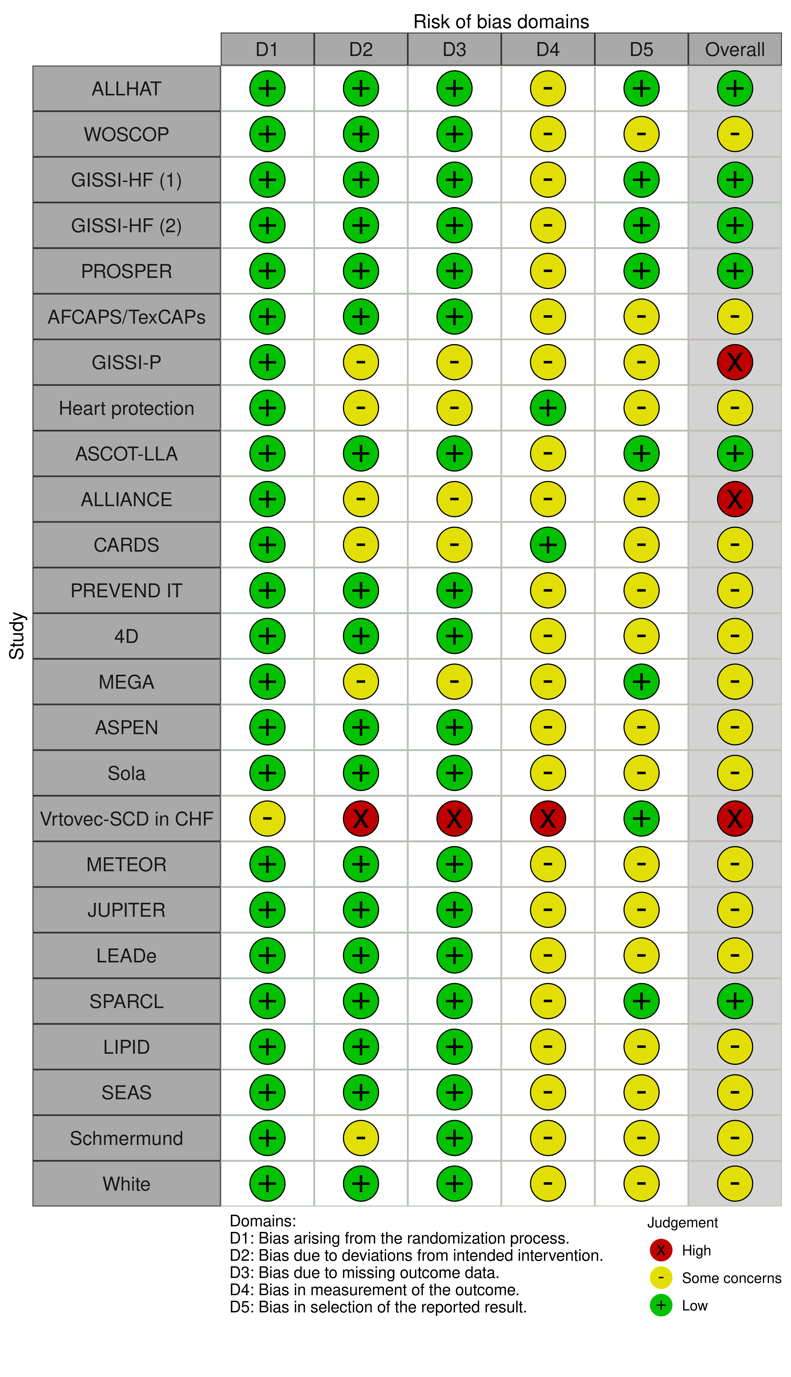


# Table S33: Risk of bias in chronic kidney disease trials.


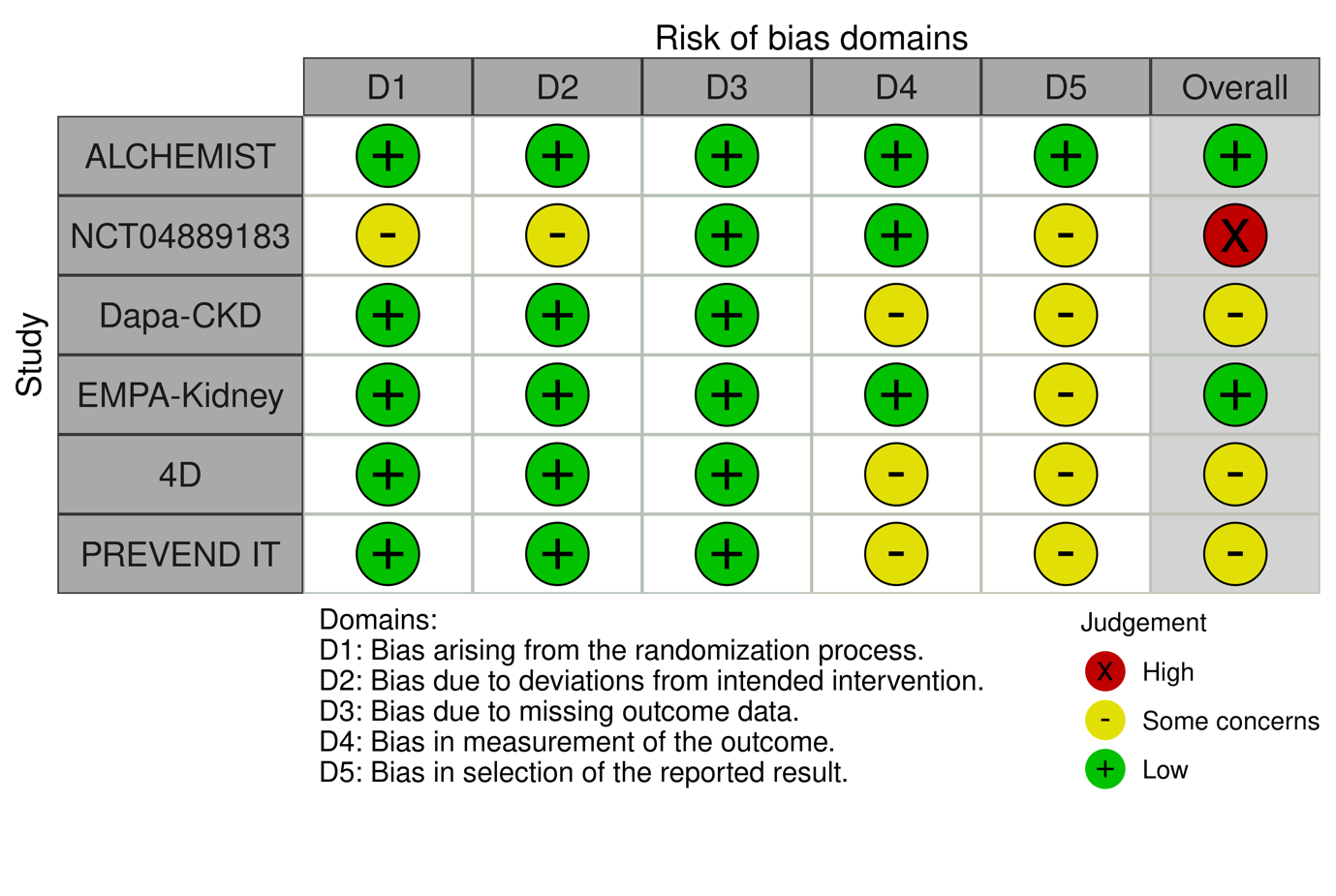


# Table S34: Risk of bias in all diabetes mellitus trials.


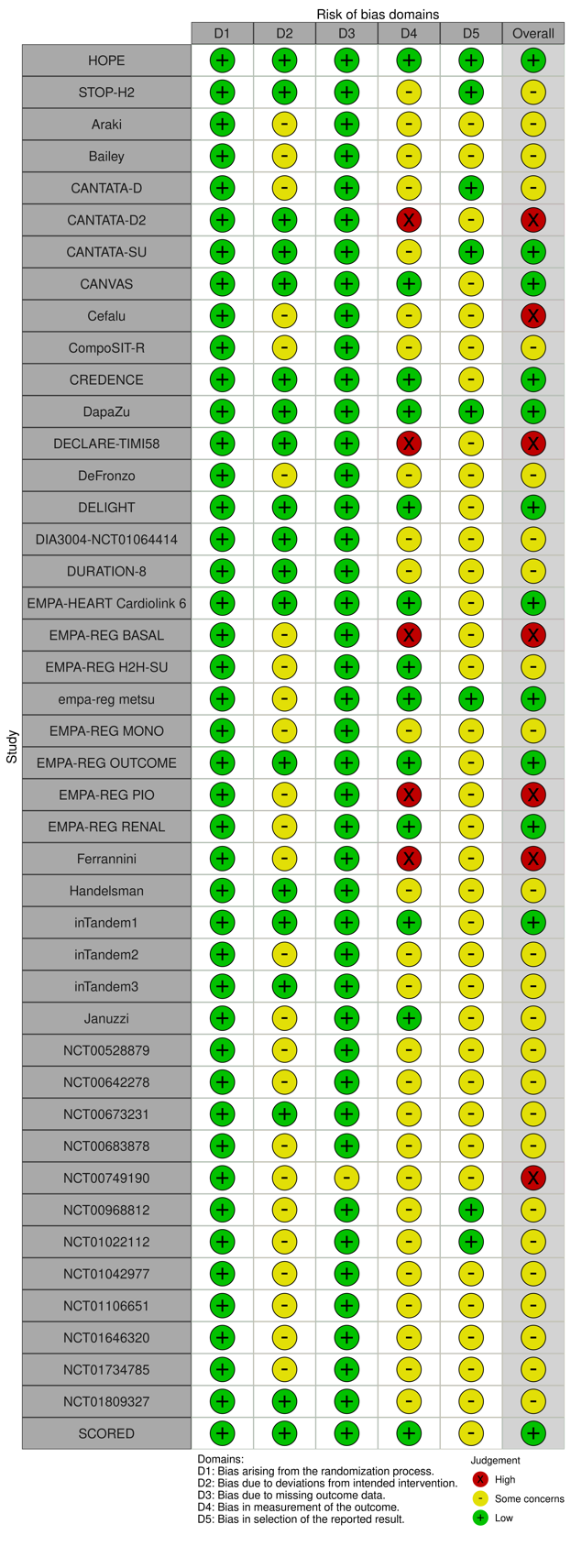

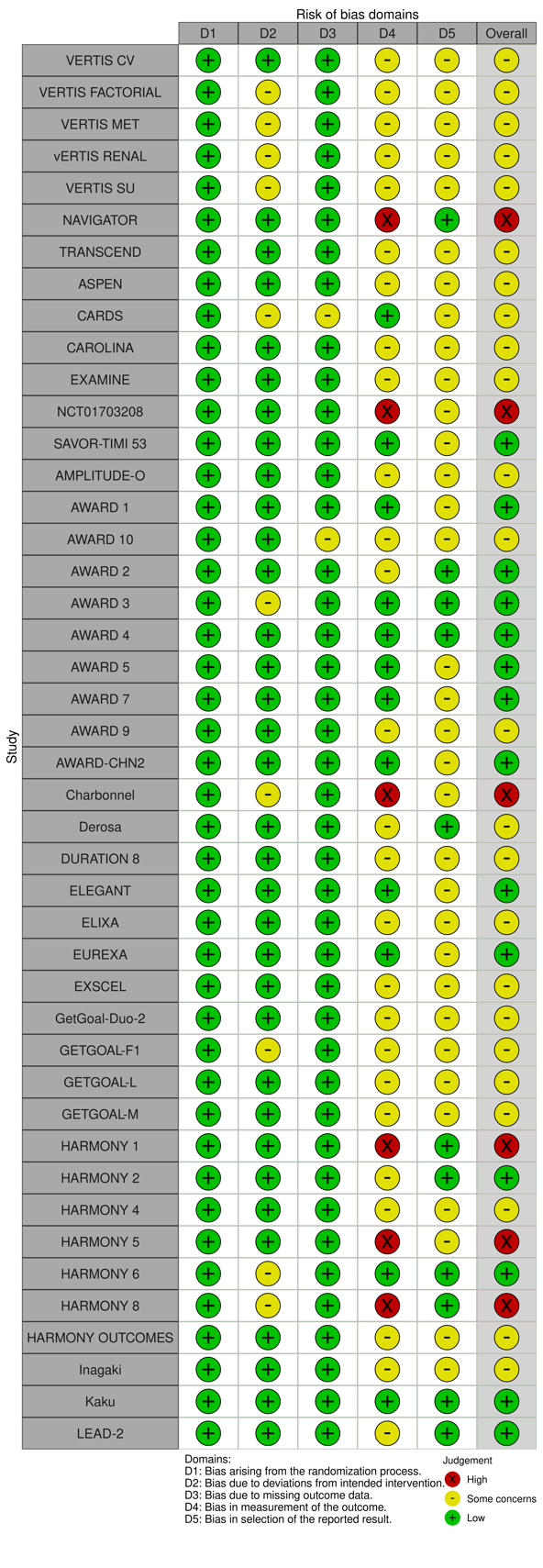


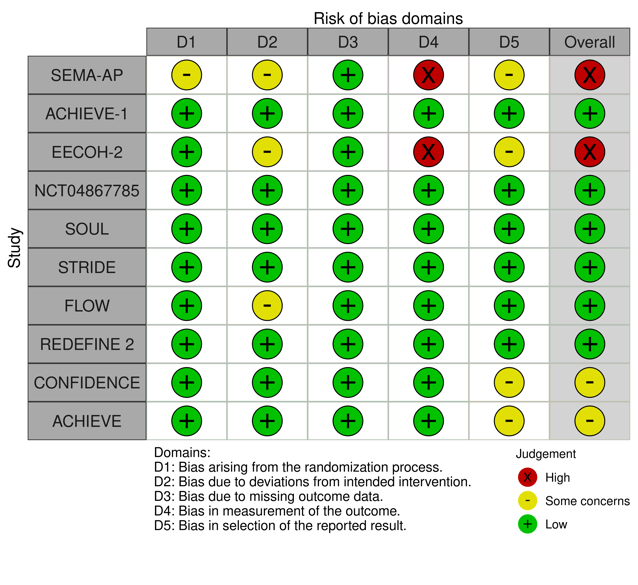

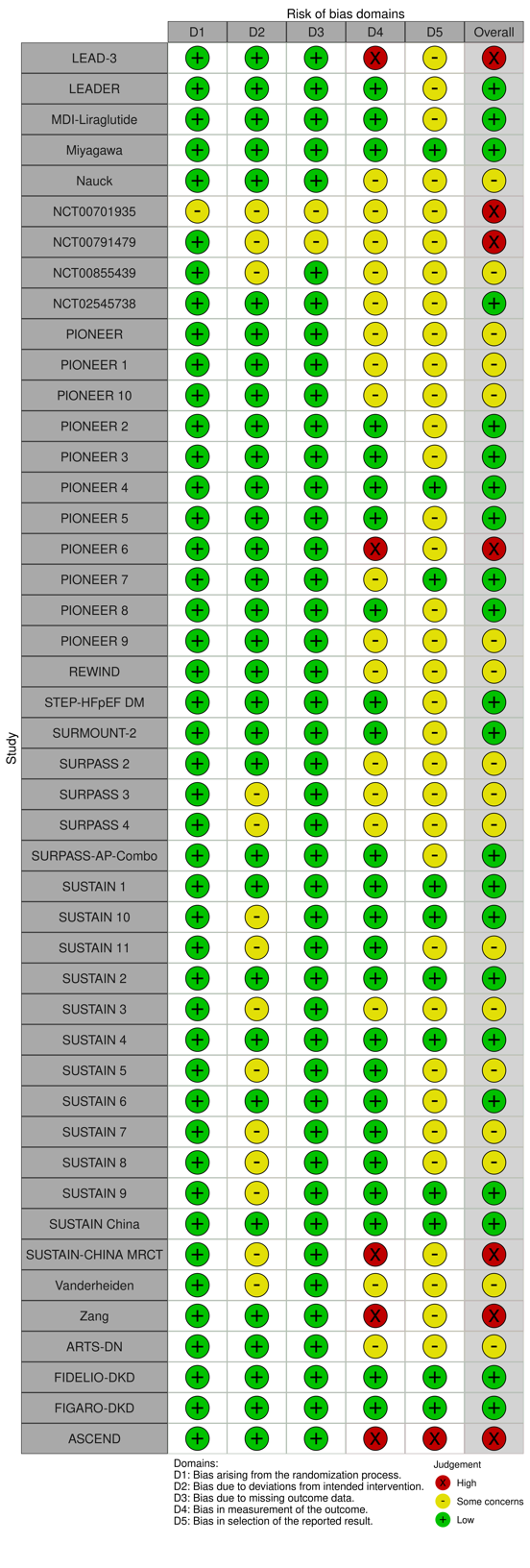


# Table S35: Risk of bias in heart failure with preserved ejection fraction trials.


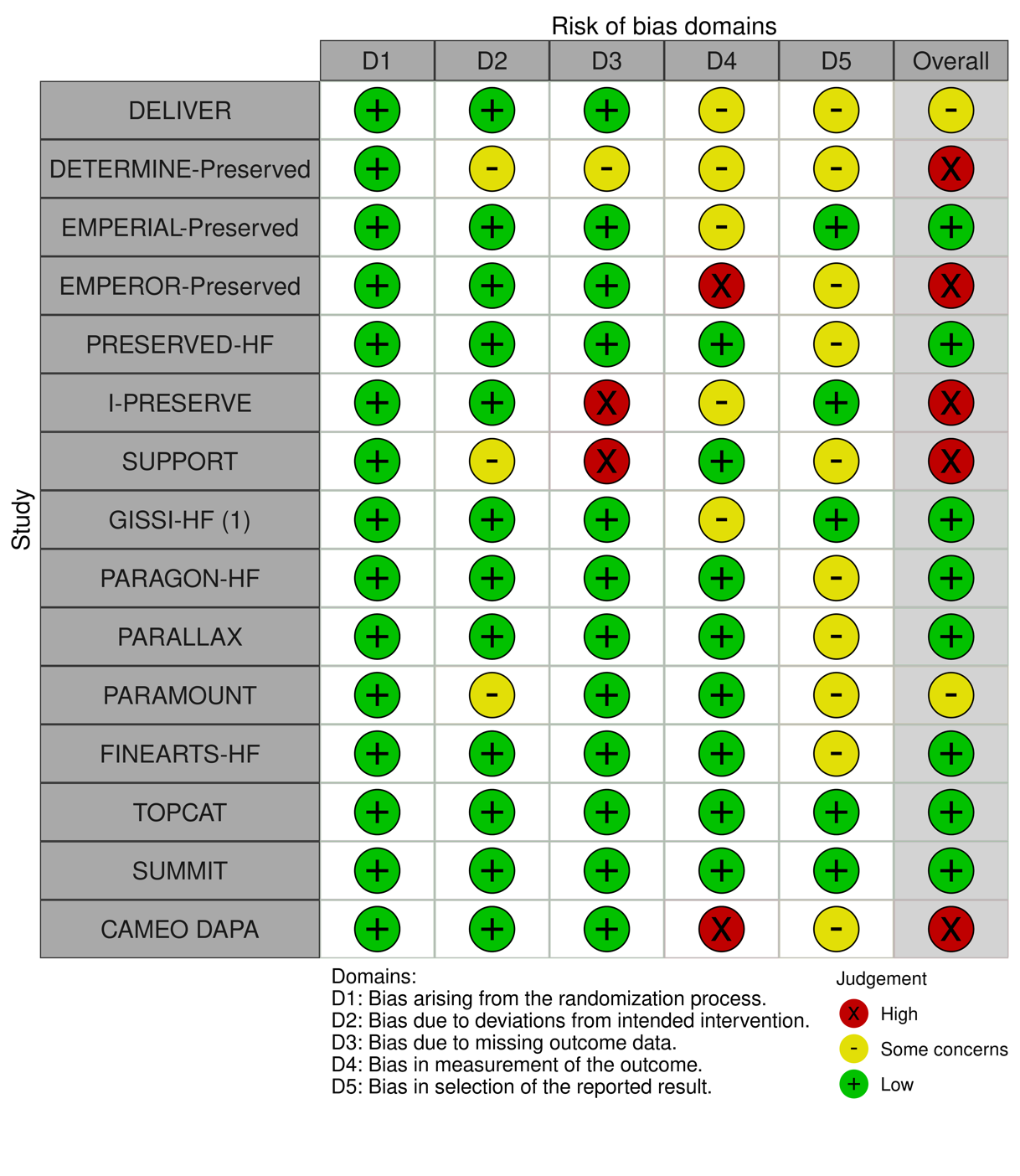


# Table S36: Risk of bias in heart failure with reduced ejection fraction trials.


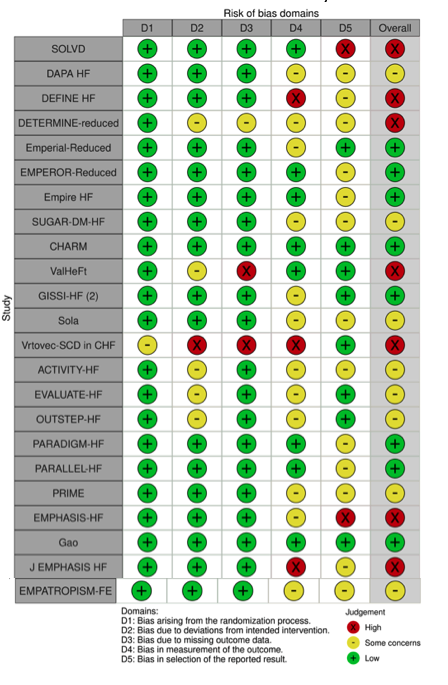


Table S37: Risk of bias in hypertension trials.


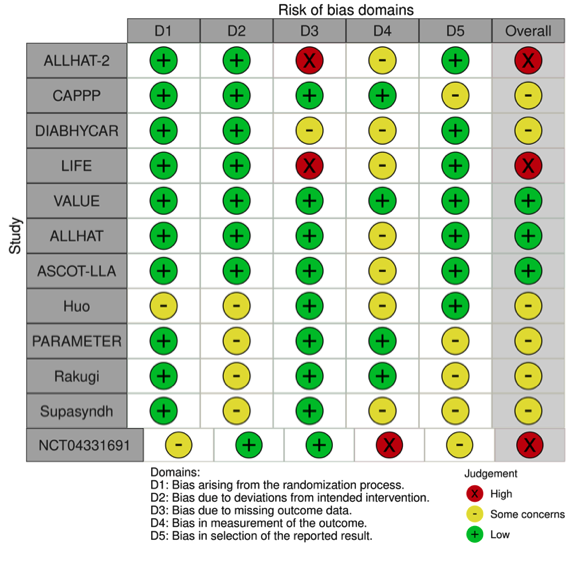


Table S38: Risk of bias in obesity trials.


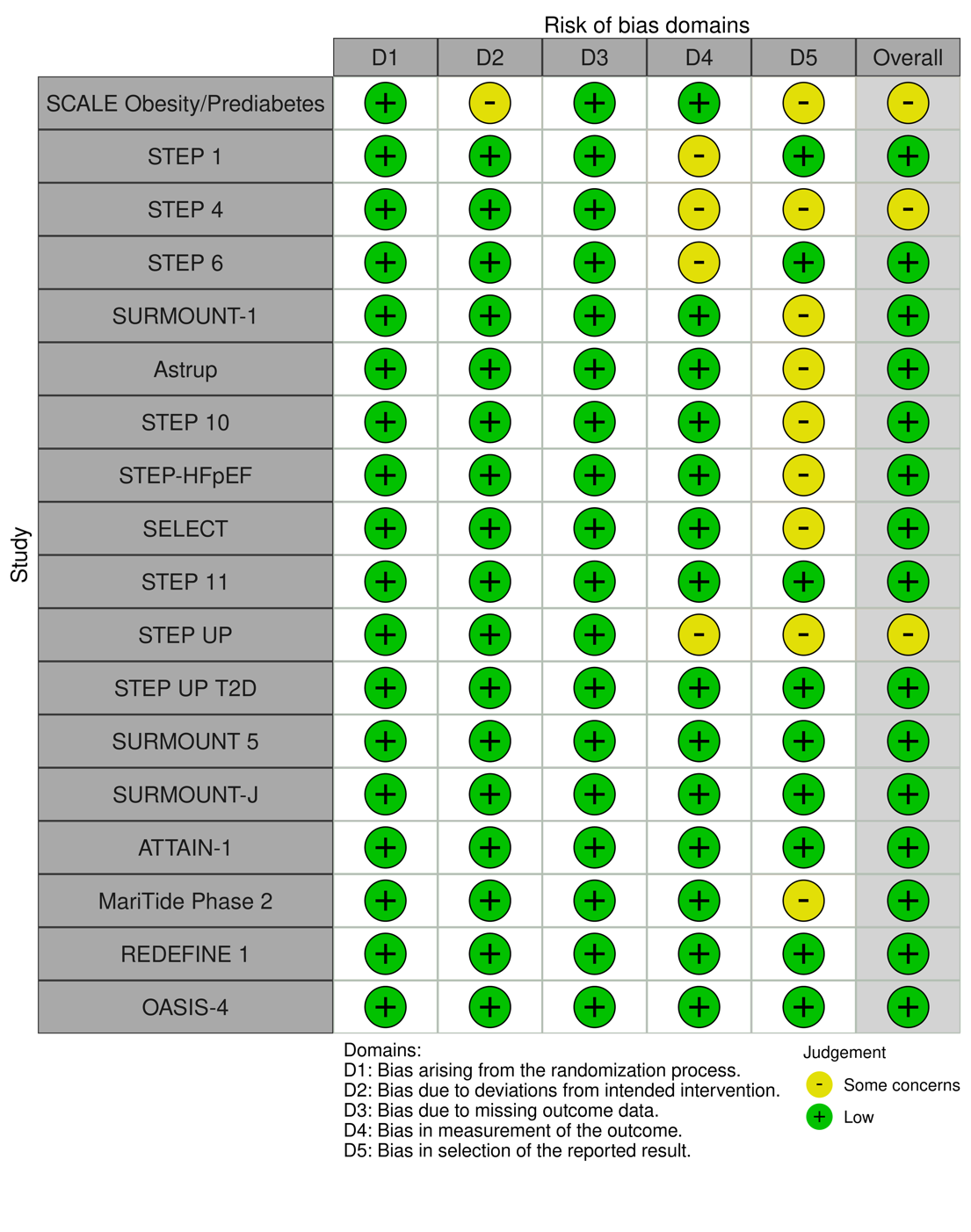


# Table S39: Risk of bias in vascular disease trials.


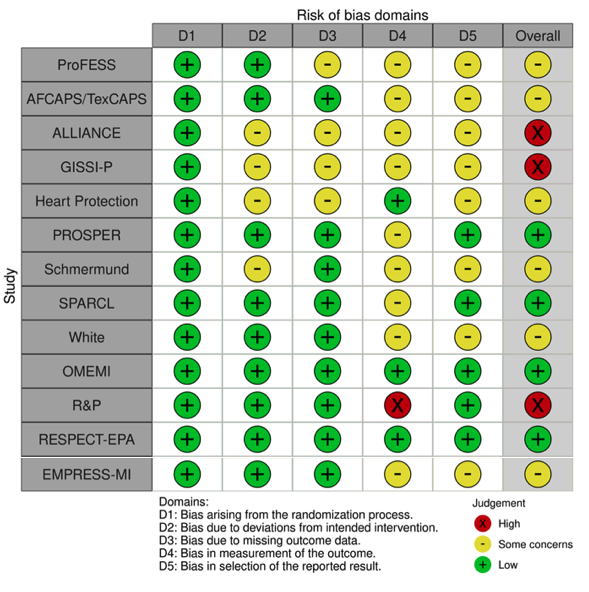


# Table S40: Risk of bias in other trials.


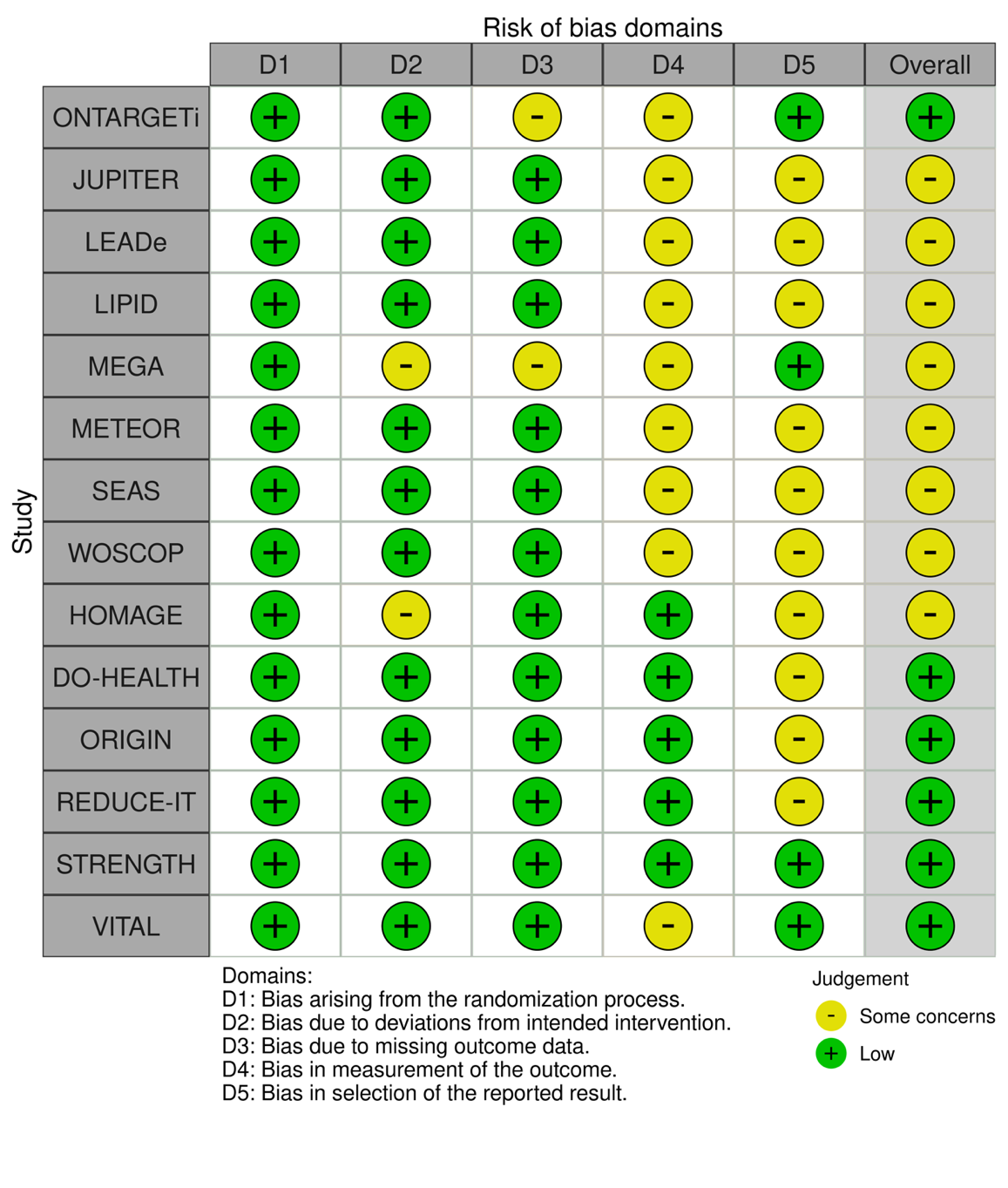


# Table S41: Summary of Abbreviations in Suppl. File 1

| A | United States of America |
| --- | --- |
| BMI | Body Mass Index |
| CHF | Congestive Heart Failure |
| DM | Diabetes Mellitus |
| E | Europe |
| ETOD | End Target Organ Damage |
| HC | Hypercholesterolaemia |
| HF | Heart Failure |
| HFpEF | Heart Failure with Preserved Ejection Fraction |
| HFrEF | Heart Failure with Reduced Ejection Fraction |
| HL | Hyperlipidaemia |
| HTN | Hypertension |
| IHD | Ischaemic Heart Disease |
| MI | Myocardial Infarction |
| O | Other countries |
| PLA | Placebo |
| PVD | Peripheral Vascular Disease |
| SBP | Systolic Blood Pressure |
| Vasc. Disease | Vascular disease |

# Table S42: PRISMA Checklist

| **Section and Topic** | **Item #** | **Checklist item** | **Location where item is reported** |
| --- | --- | --- | --- |
| **TITLE** | | |  |
| Title | 1 | Identify the report as a systematic review. | Page 1 |
| **ABSTRACT** | | |  |
| Abstract | 2 | See the PRISMA 2020 for Abstracts checklist. | Abstract |
| **INTRODUCTION** | | |  |
| Rationale | 3 | Describe the rationale for the review in the context of existing knowledge. | Pages 6, 7 |
| Objectives | 4 | Provide an explicit statement of the objective(s) or question(s) the review addresses. | Pages 6, 7 |
| **METHODS** | | |  |
| Eligibility criteria | 5 | Specify the inclusion and exclusion criteria for the review and how studies were grouped for the syntheses. | Page 8 |
| Information sources | 6 | Specify all databases, registers, websites, organisations, reference lists and other sources searched or consulted to identify studies. Specify the date when each source was last searched or consulted. | Page 8 |
| Search strategy | 7 | Present the full search strategies for all databa­ses, registers and websites, including any filters and limits used. | Supplementary material |
| Selection process | 8 | Specify the methods used to decide whether a study met the inclusion criteria of the review, including how many reviewers screened each record and each report retrieved, whether they worked independently, and if applicable, details of automation tools used in the process. | Page 9 |
| Data collection process | 9 | Specify the methods used to collect data from reports, including how many reviewers collected data from each report, whether they worked independently, any processes for obtaining or confirming data from study investigators, and if applicable, details of automation tools used in the process. | Page 9 |
| Data items | 10a | List and define all outcomes for which data were sougHTN. Specify whether all results that were compatible with each outcome domain in each study were sougHTN (e.g. for all measures, time points, analyses), and if not, the methods used to decide which results to collect. | Page 9 |
|  | 10b | List and define all other variables for which data were sougHTN (e.g. participant and intervention characteristics, funding sources). Describe any assumptions made about any missing or unclear information. | Page 9, Supplementary material |
| Study risk of bias assessment | 11 | Specify the methods used to assess risk of bias in the included studies, including details of the tool(s) used, how many reviewers assessed each study and whether they worked independently, and if applicable, details of automation tools used in the process. | Page 9 |
| Effect measures | 12 | Specify for each outcome the effect measure(s) (e.g. risk ratio, mean difference) used in the synthesis or presentation of results. | Pages 9, 10 |
| Synthesis methods | 13a | Describe the processes used to decide which studies were eligible for each synthesis (e.g. tabulating the study intervention characteristics and comparing against the planned groups for each synthesis (item #5)). | Pages 9, 10 |
|  | 13b | Describe any methods required to prepare the data for presentation or synthesis, such as handling of missing summary statistics, or data conversions. | Pages 9, 10 |
|  | 13c | Describe any methods used to tabulate or visually display results of individual studies and syntheses. | Page 10 |
|  | 13d | Describe any methods used to synthesize results and provide a rationale for the choice(s). If meta-analysis was performed, describe the model(s), method(s) to identify the presence and extent of statistical heterogeneity, and software package(s) used. | Page 10 |
|  | 13e | Describe any methods used to explore possible causes of heterogeneity among study results (e.g. subgroup analysis, meta-regression). | Page 10 |
|  | 13f | Describe any sensitivity analyses conducted to assess robustness of the synthesized results. | Page 10 |
| Reporting bias assessment | 14 | Describe any methods used to assess risk of bias due to missing results in a synthesis (arising from reporting biases). | Page 10 |
| Certainty assessment | 15 | Describe any methods used to assess certainty (or confidence) in the body of evidence for an outcome. | N/A |
| **RESULTS** | | |  |
| Study selection | 16a | Describe the results of the search and selection process, from the number of records identified in the search to the number of studies included in the review, ideally using a flow diagram. | Page 11, Figure 1 |
|  | 16b | Cite studies that migHTN appear to meet the inclusion criteria, but which were excluded, and explain why they were excluded. | Figure 1 |
| Study characteristics | 17 | Cite each included study and present its characteristics. | Table 1, Supplementary material |
| Risk of bias in studies | 18 | Present assessments of risk of bias for each included study. | Supplementary material |
| Results of individual studies | 19 | For all outcomes, present, for each study: (a) summary statistics for each group (where appropriate) and (b) an effect estimate and its precision (e.g. confidence/credible interval), ideally using structured tables or plots. | Figures 2-6, Supplementary material |
| Results of syntheses | 20a | For each synthesis, briefly summarise the characteristics and risk of bias among contributing studies. | Pages 11, 15, Supplementary material |
|  | 20b | Present results of all statistical syntheses conducted. If meta-analysis was done, present for each the summary estimate and its precision (e.g. confidence/credible interval) and measures of statistical heterogeneity. If comparing groups, describe the direction of the effect. | Pages 11-16 |
|  | 20c | Present results of all investigations of possible causes of heterogeneity among study results. | Pages 12-16 |
|  | 20d | Present results of all sensitivity analyses conducted to assess the robustness of the synthesized results. | Figures S21-S24, S48-S57, S64-S80, S86-S96 |
| Reporting biases | 21 | Present assessments of risk of bias due to missing results (arising from reporting biases) for each synthesis assessed. | N/A |
| Certainty of evidence | 22 | Present assessments of certainty (or confidence) in the body of evidence for each outcome assessed. | N/A |
| **DISCUSSION** | | |  |
| Discussion | 23a | Provide a general interpretation of the results in the context of other evidence. | Pages 17-19 |
|  | 23b | Discuss any limitations of the evidence included in the review. | Pages 20-21 |
|  | 23c | Discuss any limitations of the review processes used. | Page 20-21 |
|  | 23d | Discuss implications of the results for practice, policy, and future research. | Pages 18-20 |
| **OTHER INFORMATION** | | |  |
| Registration and protocol | 24a | Provide registration information for the review, including register name and registration number, or state that the review was not registered. | Page 5 |
|  | 24b | Indicate where the review protocol can be accessed, or state that a protocol was not prepared. | Abstract |
|  | 24c | Describe and explain any amendments to information provided at registration or in the protocol. | Abstract |
| Support | 25 | Describe sources of financial or non-financial support for the review, and the role of the funders or sponsors in the review. | Page 22 |
| Competing interests | 26 | Declare any competing interests of review authors. | Page 22 |
| Availability of data, code and other materials | 27 | Report which of the following are publicly available and where they can be found: template data collection forms; data extracted from included studies; data used for all analyses; analytic code; any other materials used in the review. | Page 23 |

# Table S43: PRISMA Abstract Checklist

| **Section and Topic** | **Item #** | **Checklist item** | **Reported (Yes/No)** |
| --- | --- | --- | --- |
| **TITLE** | | |  |
| Title | 1 | Identify the report as a systematic review. | Yes |
| **BACKGROUND** | | |  |
| Objectives | 2 | Provide an explicit statement of the main objective(s) or question(s) the review addresses. | Yes |
| **METHODS** | | |  |
| Eligibility criteria | 3 | Specify the inclusion and exclusion criteria for the review. | Yes |
| Information sources | 4 | Specify the information sources (e.g. databases, registers) used to identify studies and the date when each was last searched. | Yes |
| Risk of bias | 5 | Specify the methods used to assess risk of bias in the included studies. | N/A (full text) |
| Synthesis of results | 6 | Specify the methods used to present and synthesise results. | Yes |
| **RESULTS** | | |  |
| Included studies | 7 | Give the total number of included studies and participants and summarise relevant characteristics of studies. | Yes |
| Synthesis of results | 8 | Present results for main outcomes, preferably indicating the number of included studies and participants for each. If meta-analysis was done, report the summary estimate and confidence/credible interval. If comparing groups, indicate the direction of the effect (i.e. which group is favoured). | Yes |
| **DISCUSSION** | | |  |
| Limitations of evidence | 9 | Provide a brief summary of the limitations of the evidence included in the review (e.g. study risk of bias, inconsistency and imprecision). | Yes |
| Interpretation | 10 | Provide a general interpretation of the results and important implications. | Yes |
| **OTHER** | | |  |
| Funding | 11 | Specify the primary source of funding for the review. | Yes |
| Registration | 12 | Provide the register name and registration number. | Yes |
